# Solid-Phase Synthesis for Constructing Thiazolotriazinone-Based Compounds Library

**DOI:** 10.3390/molecules30183838

**Published:** 2025-09-22

**Authors:** Shuanghui Hua, Jimin Moon, Youngbeom Kim, Dong Jae Baek, Taeho Lee

**Affiliations:** 1 College of Pharmacy, Research Institute of Pharmaceutical Sciences, Kyungpook National University, BK21 FOUR KNU Community-Based Intelligent Novel Drug Discovery Education Unit, 80 Daehak-ro, Buk-gu, Daegu 41566, Republic of Korea; huashuanghui@naver.com (S.H.); ace_goh@krict.re.kr (J.M.); bonggu351@naver.com (Y.K.); 2 College of Pharmacy and Natural Medicine Research Institute, Mokpo National University, Mokpo 58554, Republic of Korea

**Keywords:** solid-phase synthesis, merrifield resin, thiazolotriazinone-based compounds library, chemical library

## Abstract

We describe the first solid-phase synthesis of thiazolo [4,5-*d*] [1,2,3] triazin-4(3*H*)-one derivatives using Merrifield resin. The modular sequence involves Thorpe–Ziegler cyclization, sulfone oxidation, and disulfonate nucleophilic substitution, with each step monitored by real-time ATR-FTIR spectroscopy. Conducted under mild conditions with broad functional group tolerance, the protocol delivered a library of 40 compounds in average stepwise yields of 68–97%, requiring only simple resin washing for purification. This study demonstrates a solid-phase route to thiazolotriazinones and illustrates its applicability in heterocyclic library construction and SAR studies.

## 1. Introduction

**Figure 1:**
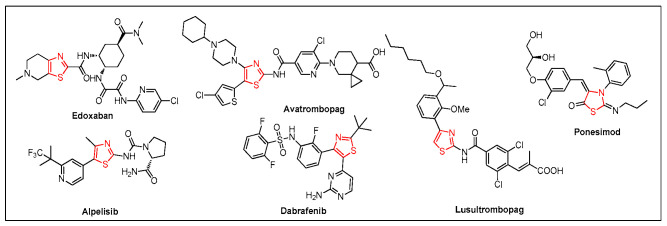
FDA-approved small-molecule thiazole-containing drugs.

**Figure 2:**
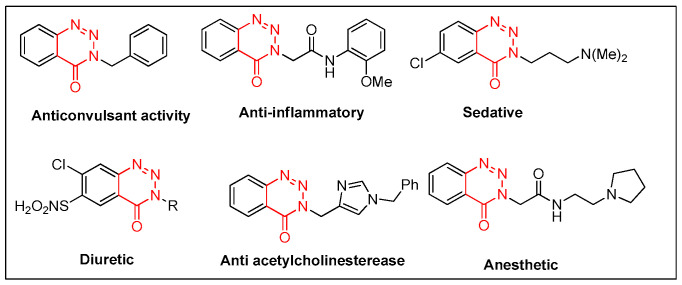
Reported benzotriazinone derivatives as bioactive compounds.

Nitrogen-containing heterocycles are frequently found in FDA-approved small-molecule drugs owing to their structural diversity and broad pharmacological activities [1,2]. Thiazoles [3] and their fused derivatives occur in anticancer, anti-inflammatory, antiviral, antibacterial, and antiparasitic agents. Representative examples include Edoxaban [4], an anticoagulant listed in the WHO’s List of Essential Medicines, avatrombopag [5], and lusutrombopag for thrombocytopenia, and alpelisib [6], and dabrafenib for breast and skin cancer therapy (Figure 1). These examples indicate the significance of the thiazole scaffold in drug discovery and suggest its potential as a core framework for combinatorial library design. Solid-phase organic synthesis (SPOS), with simplified purification and high-throughput capability, has emerged as a powerful approach in combinatorial chemistry. Our group has previously developed SPOS protocols for xanthine, thiazole, and thiophene scaffolds [7,8,9]. Benzothiazine as a nitrogen-rich fused heterocycle, has been reported to exhibit potential pharmacological activities, including anticancer [10], anticonvulsant [11], antiinflammatory [12], and anti-HIV effects [13] (Figure 2). Building on this pharmacological potential, we incorporated a thiazole-fused framework to generate thiazolotriazinone derivatives. thiazolotriazinone scaffold, as a nitrogen-rich fused heterocycle, has been reported to exhibit potential pharmacological activities such as anticancer, antibacterial, and enzyme inhibitory effects. Existing synthetic routes are predominantly based on solution-phase conditions. For example, a representative study from Beijing University disclosed a series of related compounds and their potential applications in central nervous system diseases [14]. However, most existing solution-phase synthetic routes typically involve multiple steps [15,16,17], harsh reaction conditions, and laborious purification processes, which significantly limit their applicability in combinatorial library construction. Consequently, the development of a mild and efficient synthetic strategy is of considerable importance for further studies and applications of this scaffold. To the best of our knowledge, such strategies have not yet been applied to thiazolo [4,5-*d*] [1,2,3] triazin-4(3*H*)-ones. Herein, we report the first solid-phase synthesis of thiazolotriazinone derivatives for library construction. Using Merrifield resin as the support, the modular sequence—comprising Thorpe–Ziegler cyclization, sulfone oxidation, and disulfonate nucleophilic substitution—was monitored by real-time ATR-FTIR spectroscopy. Under mild conditions, a 40-member library was efficiently prepared, providing a practical platform for accessing this scaffold and enabling subsequent structure–activity relationship studies.

## 2. Results

**Scheme 1:**
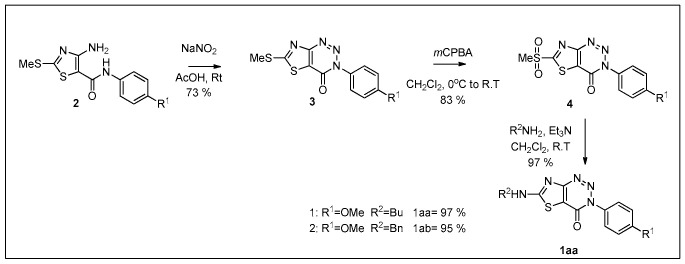
Solution-Phase Synthesis of thiazolo [4,5-*d*] [1,2,3] triazin Derivatives **1aa**.

**Scheme 2:**
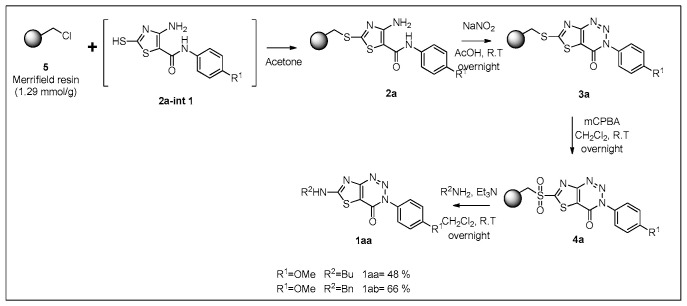
Solid-Phase Synthesis of thiazolo [4,5-*d*] [1,2,3] triazin Derivatives **1aa**.

**Figure 3:**
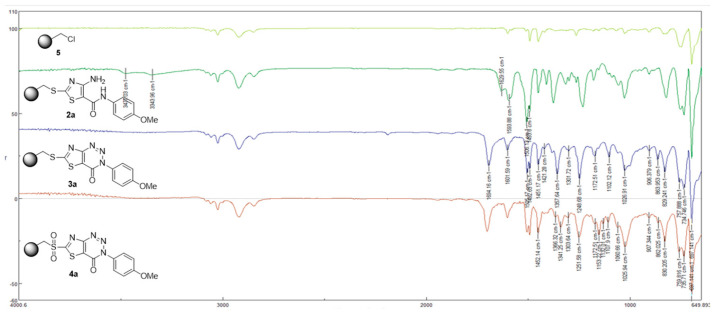
ATR-FTIR spectra of resins.

**Figure 4:**
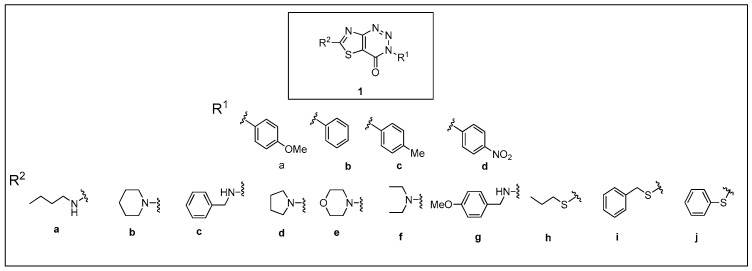
Diversity element of thiazolo [4,5-*d*] [1,2,3] triazin Derivatives **1**.

In our previous work, we systematically explored the substrate scope and optimized conditions of the Torpe–Ziegler reaction. Building upon these findings, we extended the reaction system and designed a cyclization strategy featuring ion condensation to optimize the cyclization conditions for the synthesis of triazolo [4,5-*d*] [1,2,3] triazine-4(3*H*)-one derivatives **3**. Using intermediate **2** as a model substrate, the effects of various acids, solvents, reagents, and nitro-gen sources on the cyclization were systematically investigated (Table 1). With respect to acids, the use of AcOH/H_2_O (2:1, *v/v*) afforded the product in 70% yield, whereas pure AcOH increased the yield to 85%, indicating that the presence of water significantly reduces the efficiency. Replacement with HCOOH gave a moderate yield of 61%, suggesting that stronger acidity is detrimental to cyclization. In terms of solvents, no product formation was observed in MeCN, EtOH, THF, *n*-BuOH, or acetone, and even the addition of HCl under these conditions failed to promote the reaction. These results indicate that mild pro-tic solvents are required for effective cycling. Regarding additional reagents, the use of I_2_ or NIS did not lead to product formation, indicating that these oxidants are not suitable for this transformation. As for nitrogen sources, NaNO_2_ proved to be the most effective. In contrast, NH_4_OAc failed to induce cyclization. *t*-BuONO also promoted the transformation to give the product in 80% yield, indicating that the reaction tolerates alternative nitrogen sources. Taken together, these results establish NaNO_2_ in AcOH at room temperature as the optimal condition for this cyclization. Overall, acetic acid was identified as the most suitable solvent, providing the highest yield and consistent performance, and was thus adopted as the standard condition for subsequent library synthesis. Next, compound **3** underwent oxidation with *m*-CPBA (meta-chloroperoxybenzoic acid) in DCM (dichloromethane), yielding sulfone **4** with an 83% yield. To facilitate the nucleophilic substitution reaction of compound **4** with butylamine, we synthesized **1aa** under Et_3_N/CH_2_Cl_2_ (triethylmine/dichloromethane) conditions, achieving a 98% yield. The final product was characterized using ^1^H and ^13^C NMR analysis, confirming its structure and purity (Scheme 1). This efficient and practical solution-phase synthesis strategy can be further applied to the solid-phase synthesis of thiazolotriazinone derivatives, demonstrating its wide applicability in related chemical syntheses. Next, solid-phase synthesis was performed based on the optimized conditions established in the solution-phase experiments. Merri-field resin **5** reacted with the synthesized intermediate **2** in acetone to afford the thiazole amid resin **2a**. The formation of the amide bond was confirmed by FT-IR spectroscopy, showing a characteristic NH_2_ stretching band at 3400 cm^−1^ and an amide (R-CO-NH_2_) stretching band at 1600 cm^−1^. We carried out the reaction under parallel conditions in solution-phase synthesis to make Thiazolotriazinone resin **3a** (Scheme 2) FT-IR confirmed the disappearance of NH_2_ stretching vibrations at 3477 and 3343 cm^−1^, while newly formed disubstituted (tertiary) amide C=O stretching appeared at 1693 and 1675 cm^−1^, confirming successful ring closure. Further oxidation of 3a with *m*-CPBA in DCM resulted in the sulfone **4a,** as evidenced by FT-IR spectrum showed a peak at 1342 and 1152 cm^−1^, corresponding to S=O (O=S=O) stretching vibrations (Figure 3). Finally, nucleophilic substitution at the sulfone moiety in 4a with an amine (e.g., n-butylamine for **1aa**) resulted in simultaneous desulfurization and resin cleavage, yielding the final thiazolotriazinone derivative **1aa.** Starting from Merrifield resin **5**, the target compound **1aa** was obtained in 48% overall yield via a four-step solid-phase synthesis. The final product was purified by column chromatography, and its ^1^H NMR spectrum was identical to that of the compound obtained via solution-phase synthesis, confirming structural consistency. Using this solid-phase strategy, a small library of structurally diverse thiazolotriazinone derivatives was synthesized, employing Merrifield resin **5**, 2-chloro-N-(4-methoxyphenyl) acetamide (R^1^), and various amines (R^2^) as key building blocks (Figure 4). The electronic properties of the R^1^ substituents significantly influenced the overall yield of the target thiazole [4,5-*d*] [1,2,3] triazin-4(3*H*)-one derivatives. Substrates bearing strong electron-withdrawing groups, such as nitro (R^1^ = 4-NO_2_–Ph), consistently gave lower yields regardless of the nature of the R^2^ substituent (Table 2, entries 31–40, 22–54%). In contrast, when R^1^ was an electron-donating group such as phenyl and p-methoxyphenyl, the reactions proceeded in good yields across a variety of R^2^ groups (entries 1–20, 48–86%). Regarding the R^2^ scope, the reaction exhibited broad compatibility with a range of nucleophilic components. excellent yields were obtained with benzylamines, aliphatic primary and secondary amines, and thiols (entries 1–10), highlighting the synthetic versatility and functional group tolerance of this strategy for library construction.

## 3. Materials and Methods

All chemicals used were of analytical grade and utilized without additional purification. Merrifield resin (loading capacity 1.29 mmol/g, 100–200 mesh) was obtained from Bead Tech (Seoul, Republic of Korea). Reaction progress was routinely followed by thin-layer chromatography (TLC) on silica gel 60 F-254 plates (Merck, Darmstadt, Germany). For purification, flash column chromatography was conducted with silica gel 60 (230–400 mesh, Merck). Crude compounds released from the resin were further separated using an automated Combi Flash chromatography system (Isco, Lincoln, NE, USA). NMR spectra (^1^H and ^13^C) were measured on a Bruker 500 MHz spectrometer (Bruker, Billerica, MA, USA), with chemical shifts referenced to deuterated solvents such as CDCl_3_ or DMSO-*d*6. High-performance liquid chromatography (HPLC) analysis was carried out on an Ultimate 3000 system interfaced with a Q-Exactive Focus quadrupole-Orbitrap mass spectrometer (Thermo Fisher Scientific, Waltham, MA, USA) at the Mass Spectrometry Convergence Research Institute, Kyungpook National University, Daegu, Republic of Korea). Solid-phase reactions were additionally monitored by ATR-FTIR spectroscopy using a JASCO FT-IR 4600 instrument. (JASCO, Tokyo, Japan)

### 3.1. 4-Amino-N-(4-methoxyphenyl)-2-(methylthio) thiazole-5-carboxamide (***2***)

A solution of compound **6** (73.0 mg, 0.38 mmol) in H_2_O (0.5 mL) was treated dropwise with an Acetone (2.5 mL) solution of compound 7 (50 mg, 0.25 mmol) at room temperature. The reaction was stirred for 1 h, after which LiOH (6.10 mg, 0.25 mmol) was introduced, and the mixture was refluxed at 60 °C for 2 h. Upon cooling to ambient temperature, CH_3_I (15.85 µL, 0.25 mmol) in acetone was added slowly, and the resulting solution was stirred for an additional 1 h. The crude product was isolated and purified by recrystallization from cold water to afford compound **2** (55.40 mg, 75%) as white solid: ^1^H NMR (500 MHz, CDCl_3_) δ 7.36 (d, *J* = 9.0 Hz, 2H), 6.88 (d, *J* = 9.0 Hz, 2H), 6.70 (s, 1H), 6.09 (s, 2H), 3.80 (s, 3H), 2.66 (s, 3H). ^13^C NMR (126 MHz, CDCl_3_) δ 169.02, 162.38, 162.08, 156.80, 130.54, 123.17, 114.27, 93.33, 55.54, 16.13. HRMS (ESI) *m*/*z*: [M+H] ^+^Calcd for C_12_H_14_N_3_O_2_S_2_^+^ 296.0522; Found 296.0522.

### 3.2. 3-(4-Methoxyphenyl)-6-(methylthio) thiazolo [4,5-d] [1,2,3] triazin-4(3H)-one (***3***)

Compound **6** (50 mg, 0.17 mmol) was dissolved in acetic acid, and sodium nitrite (236.0 mg, 3.39 mmol) was introduced at 0 °C. The reaction was allowed to warm to room temperature and stir for 30 min. Afterward, the mixture was diluted with CH_2_Cl_2_, washed with brine, and the organic phase was dried over MgSO4. Following solvent evaporation, the crude residue was purified by flash chromatography on silica gel (hexane/EtOAc = 1:1), affording compound **7** as a reddish solid (37.6 mg, 73%).^1^H NMR (500 MHz, CDCl_3_) δ 7.56–7.52 (m, 2H), 7.08–7.03 (m, 2H), 3.88 (s, 3H), 2.90 (s, 3H). ^13^C NMR (126 MHz, CDCl_3_) δ 178.74, 162.51, 160.36, 152.53, 130.86, 127.35, 122.19, 114.38, 55.65, 16.60. HRMS (ESI) *m*/*z*: [M+H]^+^ Calcd for C_12_H_11_N_4_O_2_S_2_^+^ 307.0318; Found 307.0318

### 3.3. 3-(4-Methoxyphenyl)-6-(methylsulfonyl) thiazolo [4,5-d] [1,2,3] triazin-4(3H)-one (***4***)

A solution of compound **3** (300 mg, 0.98 mmol) in CH_2_Cl_2_ (5 mL) was cooled to 0 °C, and *m*-CPBA (877 mg, 3.92 mmol, 77% purity) was added gradually. The mixture was then stirred at room temperature for 24 h. After completion, the reaction was quenched with saturated NaHCO_3_ solution and extracted with CH_2_Cl_2_. The combined organic phases were washed with brine, dried over MgSO_4_, and filtered. The crude material was purified by flash column chromatography on silica gel (DCM/MeOH, 60:1) to afford sulfone **4** as a white solid (261.6 mg, 83%): ^1^H NMR (500 MHz, CDCl_3_) δ 7.57–7.53 (m, 2H), 7.11–7.06 (m, 2H), 3.90 (s, 3H), 3.52 (s, 3H). ^13^C NMR (126 MHz, CDCl_3_) δ 174.78, 161.48, 160.68, 152.26, 130.27, 127.24, 127.07, 126.59, 114.57, 55.66, 42.03. HRMS (ESI) *m*/*z*: [M+H]^+^ Calcd for C_12_H_11_N_4_O_4_S_2_^+^ 339.0216; Found 339.0216

### 3.4. 6-(Butylamino)-3-(4-methoxyphenyl) thiazolo [4,5-d] [1,2,3] triazin-4(3H)-one (***1aa***)

Sulfoxide **4** (50 mg, 0.16 mmol) was dissolved in CH_2_Cl_2_ (3 mL), followed by the addition of *n*-butylamine (77.4 µL, 0.78 mmol) and triethylamine (108.6 µL, 0.78 mmol) at room temperature. The mixture was stirred for 5 min and then treated with saturated NaHCO_3_ solution, followed by extraction with CH_2_Cl_2_. The combined organic extracts were washed with brine, dried over MgSO_4_, and filtered. Purification of the crude residue by silica gel chromatography (hexane/EtOAc, 1:1) afforded compound **1aa** as a white solid (49.8 mg, 97%): ^1^H NMR (500 MHz, CDCl_3_) δ 7.53 (d, *J* = 9.0 Hz, 4H), 7.04 (d, *J* = 9.0 Hz, 4H), 6.14 (s, 2H), 3.87 (s, 6H), 3.48 (d, *J* = 6.6 Hz, 4H), 1.74 (dt, *J* = 14.8, 7.4 Hz, 4H), 1.47 (dq, *J* = 14.7, 7.4 Hz, 5H), 0.99 (t, *J* = 7.4 Hz, 6H). ^13^C NMR (126 MHz, CDCl_3_) δ 162.94, 160.14, 152.89, 131.35, 127.35, 114.25, 55.61, 46.22, 30.79, 29.68, 20.03, 13.67. HRMS (ESI) *m*/*z*: [M+H]^+^ Calcd for C_15_H_18_N_5_O_2_S^+^ 332.1176; Found 332.1176.

### 3.5. Preparation of 4-Amino-N-(substituted) thiazole-5-carboxamide Resin ***11a***

Compound **6** (2.26 g, 11.61 mmol) was dissolved in H_2_O (10 mL), and an acetone solution (50 mL) of compound 7 (1.55 g, 7.74 mmol) was introduced dropwise at room temperature. After complete addition, the reaction mixture was stirred for 1 h at ambient temperature, followed by the addition of LiOH (189.2 mg, 7.74 mmol). The mixture was then refluxed at 60 °C for 1 h. Upon cooling, the solvent was removed under reduced pressure. The residue was redissolved in acetone and subsequently combined with pre-swelled Merrifield resin 5 (2.0 g, 2.58 mmol, 1.29 mmol/g) in acetone. The suspension was agitated for 13 h at room temperature, then filtered, washed sequentially with CH_2_Cl_2_, MeOH, DMF, and H_2_O, and dried under vacuum to yield 4-amino-N-(substituted) thiazole-5-carboxamide resin **2a** (2.82 g). On-bead ATR-FTIR (neat) showed characteristic absorptions at 3480 and 3345 cm^−1^.

### 3.6. Preparation of 3-Substituted-thiazolo [4,5-d] [1,2,3] triazin-4(3H)-one Resin ***3a***

Resin **2a** (2.82 g, theoretically 3.08 mmol) was suspended in acetic acid (40 mL) together with NaNO_2_ (3.60 g, 61.58 mmol), and the mixture was stirred at room temperature for 15 h. After completion, the resin was collected by filtration, thoroughly washed with CH_2_Cl_2_, MeOH, DMF, and H_2_O, and then dried under vacuum to afford 3-substituted-thiazolo [4,5-*d*] [1,2,3] triazin-4(3*H*)-one resin **3a** (2.89 g). The product displayed characteristic on-bead ATR-FTIR absorptions at 1693 and 1675 cm^−1^.

### 3.7. Preparation of Sulfonyl 3-substituted-thiazolo [4,5-d] [1,2,3] triazin-4(3H)-one Resin ***4a***

Resin **3a** (2.87 g, theoretical 2.69 mmol) was suspended in CH_2_Cl_2_ (40 mL), and *m*-CPBA (2.31 g, 10.80 mmol, 77% purity) was added. The mixture was stirred at room temperature for 17 h. The resulting resin was collected by filtration, washed successively with CH_2_Cl_2_, MeOH, DMF, and H_2_O, and then dried under vacuum to afford sulfone resin **4a** (2.70 g). On-bead ATR-FTIR (neat) exhibited characteristic absorption bands at 1342 and 1152 cm^−1^.

### 3.8. Preparation of Thiazolo [4,5-d] [1,2,3] triazin-4(3H)-one ***1aa***

Sulfone resin **4a** (157.4 mg, theoretical 0.20 mmol) was suspended in DCM (4 mL) and reacted with *n*-butylamine (202.7 µL, 2.03 mmol) in the presence of triethylamine (284.4 µL, 2.03 mmol) at room temperature. The mixture was stirred for 18 h, after which the resin was collected by filtration, washed sequentially with DCM and MeOH, and concentrated under reduced pressure using a centrifugal vacuum evaporator. The crude residue was subjected to silica gel column chromatography, affording the target compound 6-(butylamino)-3-(4-methoxyphenyl) thiazolo [4,5-*d*] [1,2,3] triazin-4(3*H*)-one 1aa as a solid (32.2 mg, 48% yield from Merrifield resin 10, 95% purity).

## 4. Conclusions

In conclusion, we have developed a solid-phase synthesis strategy for the efficient construction of thiazolo [4,5-*d*] [1,2,3] triazin-4(3*H*)-one derivatives. By integrating mild reaction conditions, real-time ATR-FTIR monitoring, and broad functional group compatibility, this method addresses the challenges associated with harsh conditions and laborious purification in traditional solution-phase synthesis, enabling the rapid preparation of 40 structurally diverse derivatives. The resulting library not only expands the accessible chemical space of thiazole-fused heterocycles but also provides a pharmacologically relevant scaffold for SAR studies and drug discovery. Importantly, the protocol was successfully extended to other nitrogen-rich fused heterocycles, highlighting its generality and demonstrating its potential as a versatile platform for heterocyclic library construction. Taken together, this strategy underscores a practical and broadly applicable approach for accessing molecular diversity with significance in medicinal chemistry.

## Figures and Tables

**Table 1:** Optimization of reaction conditions for synthesis 3-(4-methoxyphenyl)-6-(methylthio) thiazolo [4,5-*d*] [1,2,3] triazin-4(3*H*)-one **3**.

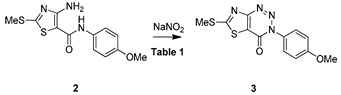						
Entry ^a^	N-Source (3 eq)	Reagent (1 eq)	Acid (3 eq)	Solvent	T °C/t h	Yield (%) ^b^
1	NaNO_2_	-	-	AcOH/H_2_O (2:1)	Rt/2 h	70
2	NaNO_2_	-	-	AcOH	Rt/1.5 h	85
3	NaNO_2_	-	-	HCOOH	Rt/2 h	61
4	NaNO_2_	-	-	MeCN	Rt/2 h	N.R ^c^
5	NaNO_2_	-	HCl	MeCN	Rt/5 h	N.R ^c^
6	NaNO_2_	-	HCl	EtOH	Rt/5 h	N.R ^c^
7	NaNO_2_	-	HCl	MeOH	Rt/5 h	N.R ^c^
8	NaNO_2_	-	HCl	THF	Rt/5 h	N.R ^c^
9	NaNO_2_	-	HCl	*n*-BuOH	Rt/5 h	N.R ^c^
10	NaNO_2_	-	HCl	Acetone	Rt/5 h	N.R ^c^
11	NaNO_2_	NIS	-	MeCN	80 °C/5 h	N.R ^c^
12	NaNO_2_	I_2_	-	MeCN	80 °C/5 h	N.R ^c^
13	*t*-BuONO	-	-	AcOH	Rt/1.5 h	80
14	NH_4_OAc	-	-	AcOH	Rt/2 h	NR ^c^

^a^ All reactions were carried out using intermediate 2 (50 mg) in 3 mL of solvent. ^b^ Isolated yield; ^c^ No reaction.

**Table 2:** Yield of thiazolo [4,5-*d*] [1,2,3] triazin derivatives library.

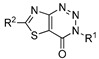							
Entry ^a^	R^1^	R^2^	Yield (%) ^b^	Entry ^a^	R^1^	R^2^	Yield (%) ^b^
**1**	a	a	48%	**21**	c	a	70%
**2**	a	b	66%	**22**	c	b	79%
**3**	a	c	75%	**23**	c	c	87%
**4**	a	d	56%	**24**	c	d	69%
**5**	a	e	61%	**25**	c	e	65%
**6**	a	f	48%	**26**	c	f	55%
**7**	a	g	53%	**27**	c	g	73%
**8**	a	h	55%	**28**	c	h	56%
**9**	a	i	60%	**29**	c	i	45%
**10**	a	g	61%	**30**	c	g	67%
**11**	b	a	75%	**31**	d	a	33%
**12**	b	b	71%	**32**	d	b	42%
**13**	b	c	48%	**33**	d	c	39%
**14**	b	d	79%	**34**	d	d	27%
**15**	b	e	86%	**35**	d	e	22%
**16**	b	f	53%	**36**	d	f	54%
**17**	b	g	74%	**37**	d	g	47%
**18**	b	h	51%	**38**	d	h	34%
**19**	b	i	30% **^c^**	**39**	d	i	38%
**20**	b	g	64%	**40**	d	g	33%

^a^ All reactions were performed on 200 mg scale of resin **4a**. ^b^ Four-step overall isolated yield from Merrifield resin **5** (loading capacity = 1.29 mmol/g). ^c^ The low yield in entry 19 was attributed to improper workup during product isolation.

## Data Availability

The Supporting Information is available free of charge on the MDPI. Publication website: ^1^H NMR, ^13^C NMR and synthetic procedure of all compounds.

## Supplementary Materials

The following supporting information can be downloaded at: https://www.mdpi.com/article/10.3390/molecules30183838/s1, “General information”, “General procedure” and “NMR spectra” are shown in Supplementary Materials.
