# Supplementary material for: Solid-Phase Synthesis for Constructing Thiazolotriazinone-Based Compounds Library
Source: Molecules. 2025 Sep 22;30(18):3838. doi: 10.3390/molecules30183838 (PMC12472383; doi:10.3390/molecules30183838)
Supplement: Supplementary file 1 [file molecules-30-03838-s001.zip › molecules-3883009-supplementary.pdf]

Supporting Information

Solid-Phase Synthesis for Constructing Thiazolotriazinone-Based Compounds Library

Shuanghui Hua [1,†], Jimin Moon [1,†], Youngbeom Kim [1,†], Dong Jae Baek [2,*] and Taeho Lee [1,*]

[1] College of Pharmacy, Research Institute of Pharmaceutical Sciences, Kyungpook National University, BK21 FOUR KNU Community-Based Intelligent Novel Drug Discovery Education Unit, 80 Daehak-ro, Buk-gu, Daegu 41566, Republic of Korea; huashuanghui@naver.com (S.H.); ace_goh@krict.re.kr (J.M.); bonggu351@naver.com (Y.K.)
[2] College of Pharmacy and Natural Medicine Research Institute, Mokpo National University,
Jeonnam 58554, Republic of Korea
* Correspondence: dbaek@mokpo.ac.kr (D.J.B.); tlee@knu.ac.kr (T.L.)
† These authors contributed equally to this work.

1. Experimental

1.1 General information

All chemicals used were of analytical grade and utilized without additional purification. Merrifield resin (loading capacity 1.29 mmol/g, 100–200 mesh) was obtained from Bead Tech (Seoul, Korea). Reaction progress was routinely followed by thin-layer chromatography (TLC) on silica gel 60 F-254 plates (Merck, Darmstadt, Germany). For purification, flash column chromatography was conducted with silica gel 60 (230–400 mesh, Merck). Crude compounds released from the resin were further separated using an automated Combi Flash chromatography system (Isco, Lincoln, NE, USA). NMR spectra (1H and 13C) were measured on a Bruker 500 MHz spectrometer (Bruker, Billerica, MA, USA), with chemical shifts referenced to deuterated solvents such as CDCl3 or DMSO-d6. High-performance liquid chromatography (HPLC) analysis was carried out on an Ulti-mate 3000 system interfaced with a Q-Exactive Focus quadrupole-Orbitrap mass spectrometer (Thermo Fisher Scientific, Mass Spectrometry Convergence Research Institute, Kyungpook National University, Daegu, Korea). Solid-phase reactions were additionally monitored by ATR-FTIR spectroscopy using a JASCO FT-IR 4600 instrument.

1.2 General procedure

4-amino-*N*-(4-methoxyphenyl)-2-(methylthio)thiazole-5-carboxamide (2)

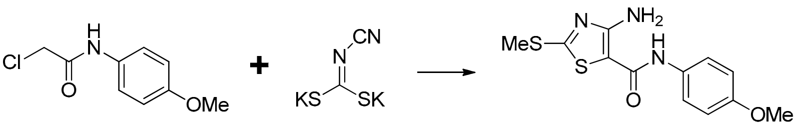

7 **6** **2**

A solution of compound **6** (73.0 mg, 0.38 mmol) in H_2O (0.5 mL) was treated dropwise with an Acetone (2.5 mL) solution of compound 7 (50 mg, 0.25 mmol) at room temperature. The reaction was stirred for 1 h, after which LiOH (6.10 mg, 0.25 mmol) was introduced, and the mixture was refluxed at 60 °C for 2 h. Upon cooling to ambient temperature, CH_3I (15.85 μL, 0.25 mmol) in acetone was added slowly, and the resulting solution was stirred for an additional 1 h. The crude product was isolated and purified by recrystallization from cold water to afford compound **2** (55.40 mg, 75 %) as white solid: ^{1}H NMR (500 MHz, $CDCl_3$) δ 7.36 (d, J = 9.0 Hz, 2H), 6.88 (d, J = 9.0 Hz, 2H), 6.70 (s, 1H), 6.09 (s, 2H), 3.80 (s, 3H), 2.66 (s, 3H). ^{13}C NMR (126 MHz, $CDCl_3$) δ 169.02, 162.38, 162.08, 156.80, 130.54, 123.17, 114.27, 93.33, 55.54, 16.13. HRMS (ESI) *m/z*: $[M+H]^+$Calcd for $C_{12}H_{14}N_3O_2S_2^+$ 296.0522; Found 296.0522

3-(4-methoxyphenyl)-6-(methylthio) thiazolo[4,5-*d*][1,2,3]triazin-4(3*H*)-one (3)

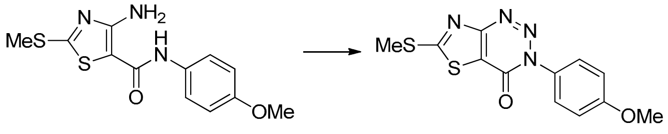

2 **3**

Compound **6** (50 mg, 0.17 mmol) was dissolved in acetic acid, and sodium nitrite (236.0 mg, 3.39 mmol) was introduced at 0 °C. The reaction was allowed to warm to room temperature and stir for 30 min. Afterward, the mixture was diluted with CH_2Cl_2, washed with brine, and the organic phase was dried over MgSO4. Following solvent evaporation, the crude residue was purified by flash chromatography on silica gel (hexane/EtOAc = 1:1), affording compound **7** as a reddish solid (37.6 mg, 73%): ^{1}H NMR (500 MHz, $CDCl_3$) δ 7.56 – 7.52 (m, 2H), 7.08 – 7.03 (m, 2H), 3.88 (s, 3H), 2.90 (s, 3H). ^{13}C NMR (126 MHz, $CDCl_3$) δ 178.74, 162.51, 160.36, 152.53, 130.86, 127.35, 122.19, 114.38, 55.65, 16.60. HRMS (ESI) *m/z*: $[M+H]^+$Calcd for $C_{12}H_{11}N_4O_2S_2^+$ 307.0318; Found 307.0318

3-(4-methoxyphenyl)-6-(methylsulfonyl) thiazolo[4,5-*d*][1,2,3]triazin-4(3*H*)-one (4)

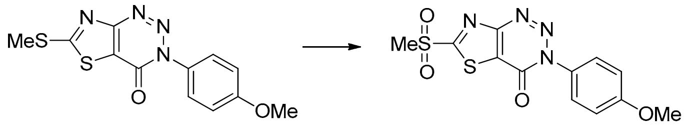

3 4

A solution of compound **3** (300 mg, 0.98 mmol) in CH_2Cl_2 (5 mL) was cooled to 0 °C, and *m*-CPBA (877 mg, 3.92 mmol, 77% purity) was added gradually. The mixture was then stirred at room temperature for 24 h. After completion, the reaction was quenched with saturated $NaHCO_3$ solution and extracted with CH_2Cl_2. The combined organic phases were washed with brine, dried over MgSO4, and filtered. The crude material was purified by flash column chromatography on silica gel (DCM/MeOH, 60:1) to afford sulfone **4** as a white solid (261.6 mg, 83%): ^{1}H NMR (500 MHz, $CDCl_3$) δ 7.57 – 7.53 (m, 2H), 7.11 – 7.06 (m, 2H), 3.90 (s, 3H), 3.52 (s, 3H). ^{13}C NMR (126 MHz, $CDCl_3$) δ 174.78, 161.48, 160.68, 152.26, 130.27, 127.24, 127.07, 126.59, 114.57, 55.66, 42.03. HRMS (ESI) *m/z*: $[M+H]^+$Calcd for $C_{12}H_{11}N_4O_4S_2^+$ 339.0216; Found 339.0216

6-(butylamino)-3-(4-methoxyphenyl) thiazolo[4,5-*d*][1,2,3]triazin-4(3*H*)-one (1aa)

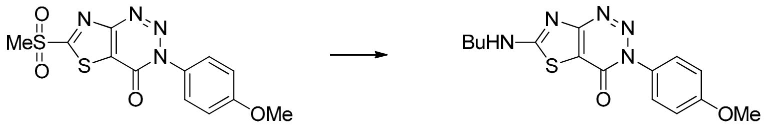

4 1aa

Sulfoxide **4** (50 mg, 0.16 mmol) was dissolved in CH_2Cl_2 (3 mL), followed by the addition of *n*-butylamine (77.4 μL, 0.78 mmol) and triethylamine (108.6 μL, 0.78 mmol) at room temperature. The mixture was stirred for 5 min and then treated with saturated $NaHCO_3$ solution, followed by extraction with CH_2Cl_2. The combined organic extracts were washed with brine, dried over $MgSO_4$, and filtered. Purification of the crude residue by silica gel chromatography (hexane/EtOAc, 1:1) afforded compound **1aa** as a white solid : ^{1}H NMR (500 MHz, $CDCl_3$) δ 7.53 (d, J = 9.0 Hz, 4H), 7.04 (d, J = 9.0 Hz, 4H), 6.14 (s, 2H), 3.87 (s, 6H), 3.48 (d, J = 6.6 Hz, 4H), 1.74 (dt, J = 14.8, 7.4 Hz, 4H), 1.47 (dq, J = 14.7, 7.4 Hz, 5H), 0.99 (t, J = 7.4 Hz, 6H). ^{13}C NMR (126 MHz, $CDCl_3$) δ 162.94, 160.14, 152.89, 131.35, 127.35, 114.25, 55.61, 46.22, 30.79, 29.68, 20.03, 13.67. HRMS (ESI) *m/z*: $[M+H]^+$Calcd for $C_{15}H_{18}N_5O_2S^+$ 332.1176; Found 332.1176

Preparation of 4-amino-*N*-(substituted)thiazole-5-carboxamide resin 11a.

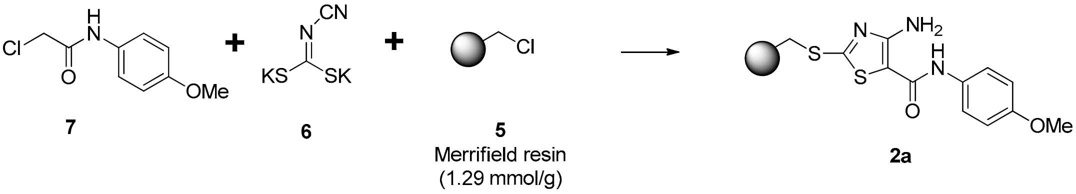

Compound 6 (2.26 g, 11.61 mmol) was dissolved in H_2O (10 mL), and an acetone solution (50 mL) of compound 7 (1.55 g, 7.74 mmol) was introduced dropwise at room temperature. After complete addition, the reaction mixture was stirred for 1 h at ambient temperature, followed by the addition of LiOH (189.2 mg, 7.74 mmol). The mixture was then refluxed at 60 °C for 1 h. Upon cooling, the solvent was removed under reduced pressure. The residue was redissolved in acetone and subsequently combined with pre-swelled Merrifield resin 5 (2.0 g, 2.58 mmol, 1.29 mmol/g) in acetone. The suspension was agitated for 13 h at room temperature, then filtered, washed sequentially with CH_2Cl_2, MeOH, DMF, and H_2O, and dried under vacuum to yield 4-amino-N-(substituted) thiazole-5-carboxamide resin **2a** (2.82 g). On-bead ATR-FTIR (neat) showed characteristic absorptions at 3480 and 3345 cm^{-1}.

Preparation of 3-substituted-thiazolo[4,5-*d*][1,2,3] triazin-4(3*H*)-one resin 3a

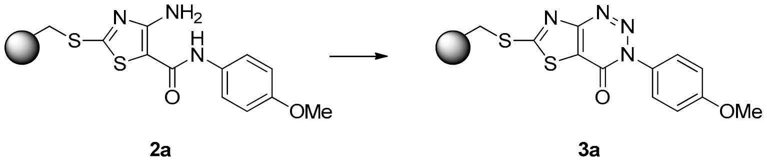

Resin 2a (2.82 g, theoretically 3.08 mmol) was suspended in acetic acid (40 mL) to-gether with $NaNO_2$ (3.60 g, 61.58 mmol), and the mixture was stirred at room temperature for 15 h. After completion, the resin was collected by filtration, thoroughly washed with CH_2Cl_2, MeOH, DMF, and H_2O, and then dried under vacuum to afford the 3-substituted-thiazolo[4,5-d][1,2,3]triazin-4(3*H*)-one resin 3a (2.89 g). The product displayed characteristic on-bead ATR-FTIR absorptions at 1693 and 1675 cm^{-1}.

Preparation of sulfonyl 3-substituted-thiazolo[4,5-*d*][1,2,3] triazin-4(3*H*)-one resin 4a.

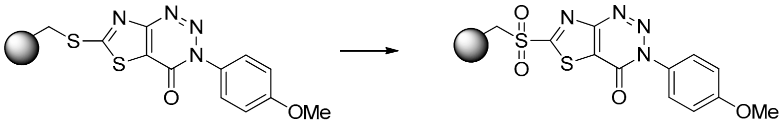

Resin 3a (2.87 g, theoretical 2.69 mmol) was suspended in CH_2Cl_2 (40 mL), and m-CPBA (2.31 g, 10.80 mmol, 77% purity) was added. The mixture was stirred at room temperature for 17 h. The resulting resin was collected by filtration, washed successively with CH_2Cl_2, MeOH, DMF, and H_2O, and then dried under vacuum to afford sulfone resin 4a (2.70 g). On-bead ATR-FTIR (neat) exhibited characteristic absorption bands at 1342 and 1152 cm^{-1}.

Preparation of thiazolo[4,5-*d*][1,2,3] triazin-4(3*H*)-one 1aa.

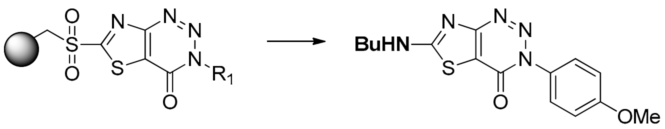

Sulfone resin 4a (157.4 mg, theoretical 0.20 mmol) was suspended in DCM (4 mL) and reacted with n-butylamine (202.7 μL, 2.03 mmol) in the presence of triethylamine (284.4 μL, 2.03 mmol) at room temperature. The mixture was stirred for 18 h, after which the resin was collected by filtration, washed sequentially with DCM and MeOH, and concentrated under reduced pressure using a centrifugal vacuum evaporator. The crude residue was subjected to silica gel column chromatography, affording the target compound 6-(butylamino)-3-(4-methoxyphenyl) thiazolo[4,5-*d*] [1,2,3] triazin-4(3*H*)-one 1aa as a solid (32.2 mg, 48% yield from Merrifield resin 10, 95% purity).

3-(4-methoxyphenyl)-6-(piperidin-1-yl)thiazolo[4,5-*d*][1,2,3]triazin-4(3*H*)-one (1ab).

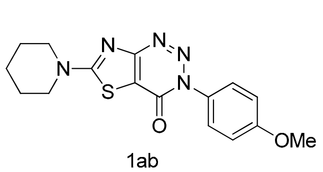

1ab

Isolated yield: 66 %; 95 % purity. Red ash solid. ^1H NMR (500 MHz, $CDCl_3$) δ 7.55 – 7.50 (m, 2H), 7.05 – 7.00 (m, 2H), 3.87 (s, 3H), 3.72 (s, 4H), 1.76 (s, 6H).

^{13}C NMR (126 MHz, $CDCl_3$) δ 171.68, 161.17, 157.69, 152.13, 131.12, 124.78, 114.21, 51.00, 23.95, 18.32. HRMS (ESI) *m/z*: [M+H] $^+$Calcd for $C_{16}H_{18}N_5O_2S^+$ 344.1176; Found 344.1176

6-(benzylamino)-3-(4-methoxyphenyl)thiazolo[4,5-*d*][1,2,3]triazin-4(3*H*)-one (1ac).

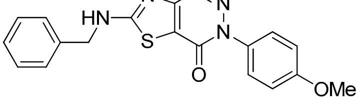

1ac

Isolated yield: 75 %; 95 % purity. Orange solid ^1H NMR (500 MHz, $CDCl_3$) δ 7.54 – 7.49 (m, 2H), 7.42 – 7.32 (m, 5H), 7.05 – 7.01 (m, 2H), 6.76 (s, 1H), 4.70 (d, J = 5.4 Hz, 2H), 3.87 (s, 3H). ^{13}C NMR (126 MHz, $CDCl_3$) δ 162.66, 160.17, 152.94, 135.72, 131.26, 128.94, 128.25, 127.97, 127.34, 114.27, 113.81, 55.62, 49.60. HRMS (ESI) *m/z*: [M+H] $^+$Calcd for $C_{18}H_{16}N_5O_2S^+$ 366.1019; Found 366.1019

3-(4-methoxyphenyl)-6-(pyrrolidin-1-yl)thiazolo[4,5-*d*][1,2,3]triazin-4(3*H*)-one (1ad).

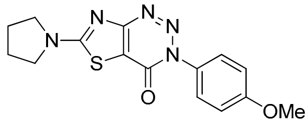

1ad

Isolated yield: 55 %; 95 % purity. Purple solid. ^1H NMR (500 MHz, $CDCl_3$) δ 7.56 – 7.50 (m, 2H), 7.05 – 6.99 (m, 2H), 3.87 (s, 3H), 3.45 (d, J = 6.6 Hz, 4H), 2.16 (s, 4H). ^{13}C NMR (126 MHz, $CDCl_3$) δ 169.63, 163.50, 160.06, 152.98, 131.47, 127.39, 114.20, 55.61, 50.29, 25.70. HRMS (ESI) *m/z*: [M+H] $^+$Calcd for $C_{15}H_{16}N_5O_2S^+$ 330.1019; Found 330.1019

3-(4-methoxyphenyl)-6-morpholinothiazolo[4,5-*d*][1,2,3] triazin-4(3*H*)-one (1ae).

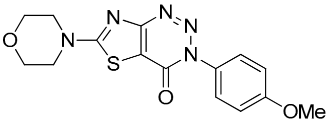

1ae

Isolated yield: 61 %; 95 % purity. Yellow solid. ^1H NMR (500 MHz, $CDCl_3$) δ 7.53 (d, J = 9.0 Hz, 2H), 7.03 (d, J = 9.0 Hz, 2H), 3.86 (dd, J = 12.7, 7.3 Hz, 7H), 3.76

(d, J = 16.0 Hz, 4H). ^{13}C NMR (126 MHz, $CDCl_3$) δ 173.40, 163.08, 160.16, 152.79, 131.31, 127.35, 114.25, 114.17, 65.98, 55.62, 48.65. HRMS (ESI) *m/z*: $[M+H]^+$Calcd for $C_{15}H_{16}N_5O_3S^+$346.0968; Found 346.0969

6-(diethylamino)-3-(4-methoxyphenyl)thiazolo[4,5-*d*][1,2,3]triazin-4(3*H*)-one (1af).

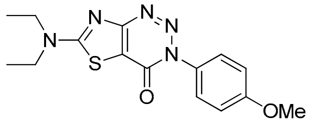

1af

Isolated yield: 48 %; 95 % purity. Yellow solid. ^1H NMR (500 MHz, $CDCl_3$) δ 7.53 – 7.49 (m, 2H), 7.04 – 6.99 (m, 2H), 3.86 (s, 3H), 3.65 (s, 4H), 1.33 (t, J = 7.2 Hz, 6H). ^{13}C NMR (126 MHz, $CDCl_3$) δ 172.01, 163.55, 160.07, 152.88, 131.47, 127.39, 114.21, 113.51, 55.61, 12.39. HRMS (ESI) *m/z*: $[M+H]^+$Calcd for $C_{15}H_{18}N_5O_2S^+$ 332.1176; Found 332.1175

6-((4-methoxybenzyl)amino)-3-(4-methoxyphenyl)thiazolo[4,5-*d*][1,2,3]triazin-4(3*H*)-one (1ag).

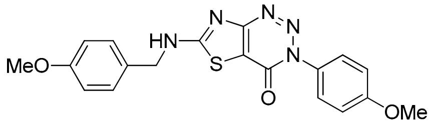

1ag

Isolated yield: 54 %; 95 % purity. Orange solid. ^1H NMR (500 MHz, $CDCl_3$) δ 7.53 – 7.48 (m, 2H), 7.33 (t, J = 5.8 Hz, 2H), 7.05 – 7.00 (m, 2H), 6.92 – 6.88 (m, 2H), 4.61 (d, J = 5.3 Hz, 2H), 3.87 (s, 3H), 3.81 (s, 3H). ^{13}C NMR (126 MHz, $CDCl_3$) δ 161.71, 157.67, 148.19, 129.48, 127.35, 114.44, 114.26, 55.50. HRMS (ESI) *m/z*: $[M+H]^+$Calcd for $C_{19}H_{18}N_5O_3S^+$396.1125; Found 396.1128

3-(4-methoxyphenyl)-6-(propylthio)thiazolo[4,5-*d*][1,2,3]triazin-4(3*H*)-one (1ah).

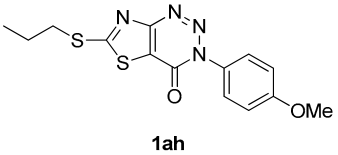

1ah

Isolated yield: 56 %; 95 % purity. Ivory solid. ^1H NMR (500 MHz, $CDCl_3$) δ 7.55 –

7.50 (m, 2H), 7.06 – 7.01 (m, 2H), 3.91 – 3.82 (m, 3H), 3.44 (t, J = 7.2 Hz, 2H), 1.94 – 1.85 (m, 2H), 1.10 (t, J = 7.4 Hz, 3H). ^{13}C NMR (126 MHz, $CDCl_3$) δ 178.28, 162.52, 160.33, 152.46, 130.91, 127.34, 122.11, 114.35, 55.64, 36.22, 22.37, 13.33. HRMS (ESI) *m/z*: [M+H] $^+$Calcd for $C_{14}H_{15}N_4O_2S_2^+$335.0631; Found 335.0630

6-(benzylthio)-3-(4-methoxyphenyl)thiazolo[4,5-*d*][1,2,3]triazin-4(3*H*)-one (1ai).

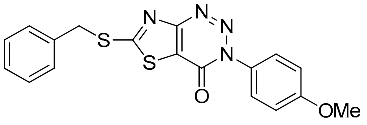

1ai

Isolated yield: 60 %; 95 % purity. Brown solid. ^{1}H NMR (500 MHz, $CDCl_3$) δ 7.55 – 7.51 (m, 2H), 7.49 – 7.46 (m, 2H), 7.38 – 7.30 (m, 3H), 7.07 – 7.03 (m, 2H), 4.70 (s, 2H), 3.88 (s, 3H). ^{13}C NMR (126 MHz, $CDCl_3$) δ 177.21, 162.36, 160.36, 152.48, 134.86, 130.88, 129.29, 128.96, 128.29, 127.34, 122.40, 114.38, 55.64, 38.34. HRMS (ESI) *m/z*: [M+H] $^+$Calcd for $C_{18}H_{15}N_4O_2S_2^+$383.0631; Found 383.0632

3-(4-methoxyphenyl)-6-(phenylthio)thiazolo[4,5-*d*][1,2,3]triazin-4(3*H*)-one (1aj).

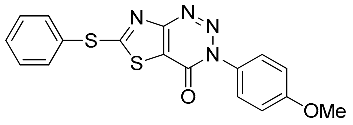

1aj

Isolated yield: 61 %; 95 % purity. Ivory solid. ^{1}H NMR (500 MHz, $CDCl_3$) δ 7.76 (dd, J = 5.2, 3.2 Hz, 2H), 7.64 – 7.55 (m, 3H), 7.51 – 7.47 (m, 2H), 7.04 – 7.00 (m, 2H), 3.86 (s, 3H). ^{13}C NMR (126 MHz, $CDCl_3$) δ 182.17, 163.22, 160.30, 152.22, 135.82, 131.99, 130.90, 127.94, 127.29, 122.39, 114.35, 55.63. HRMS (ESI) *m/z*: [M+H] $^+$Calcd for $C_{17}H_{13}N_4O_2S_2^+$ 369.0474; Found 369.0474

6-(butylamino)-3-phenylthiazolo[4,5-*d*][1,2,3]triazin-4(3*H*)-one (1ba).

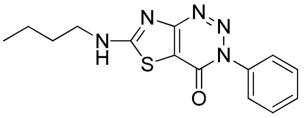

1ba

Isolated yield: 76 %; 95 % purity. White, pink solid. ^{1}H NMR (500 MHz, $CDCl_3$) δ 8.30 (s, 1H), 7.64 – 7.58 (m, 2H), 7.57 – 7.46 (m, 3H), 3.46 (dd, J = 12.8, 6.7 Hz, 2H), 1.86 – 1.75 (m, 2H), 1.51 – 1.41 (m, 2H), 0.97 (t, J = 7.4 Hz, 3H). ^{13}C NMR (126 MHz, $CDCl_3$) δ 162.96, 152.79, 138.50, 129.35, 129.07, 126.17, 46.37, 30.70, 20.07, 13.69. HRMS (ESI) *m/z*: [M+H] $^+$Calcd for $C_{14}H_{16}N_5OS^+$ 302.1070; Found 302.1070

3-phenyl-6-(piperidin-1-yl)thiazolo[4,5-*d*][1,2,3]triazin-4(3*H*)-one (1bb).

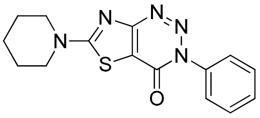

1bb

Isolated yield: 71 %; 95 % purity. Purple solid. ^{1}H NMR (500 MHz, $CDCl_3$) δ 7.65 – 7.57 (m, 2H), 7.56 – 7.43 (m, 3H), 3.71 (s, 4H), 1.74 (s, 6H). ^{13}C NMR (126 MHz, $CDCl_3$) δ 172.96, 163.51, 152.69, 138.60, 129.20, 128.99, 126.16, 113.63, 50.13, 25.23, 23.85. HRMS (ESI) *m/z*: [M+H] $^+$Calcd for $C_{15}H_{16}N_5OS^+$ 314.1070; Found 314.1070

6-(benzylamino)-3-phenylthiazolo[4,5-*d*][1,2,3]triazin-4(3*H*)-one (1bc).

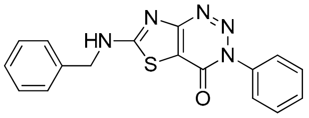

1bc

Isolated yield: 48 %; 95 % purity. Pink solid. ^{1}H NMR (500 MHz, $CDCl_3$) δ 8.03 (s, 1H), 7.63 – 7.56 (m, 2H), 7.56 – 7.46 (m, 3H), 7.41 (d, J = 7.1 Hz, 2H), 7.39 – 7.30 (m, 3H), 4.69 (d, J = 5.4 Hz, 2H). ^{13}C NMR (126 MHz, $CDCl_3$) δ 162.64, 152.74, 138.43, 129.37, 129.08, 129.04, 128.40, 128.00, 126.13, 49.73. HRMS (ESI) *m/z*: [M+H] $^+$Calcd for $C_{17}H_{14}N_5OS^+$ 336.0914; Found 336.0914

3-phenyl-6-(pyrrolidin-1-yl)thiazolo[4,5-*d*][1,2,3]triazin-4(3*H*)-one (1bd).

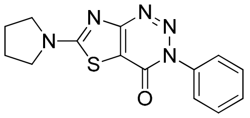

1bd

Isolated yield: 79 %; 95 % purity. Kaki solid. ^{1}H NMR (500 MHz, $CDCl_3$) δ 7.54

(d, J = 8.2 Hz, 2H), 7.42 (ddd, J = 14.5, 11.7, 4.1 Hz, 3H), 3.56 (d, J = 195.1 Hz, 4H), 2.07 (s, 4H). ^{13}C NMR (126 MHz, $CDCl_3$) δ 169.65, 163.46, 152.78, 138.60, 129.18, 128.98, 126.17, 113.73, 50.31, 25.68. HRMS (ESI) *m/z*: $[M+H]^+$Calcd for $C_{14}H_{14}N_5OS^+$300.0914; Found 300.0914

6-morpholino-3-phenylthiazolo[4,5-*d*][1,2,3]triazin-4(3*H*)-one (1be).

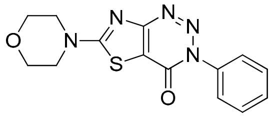

1be

Isolated yield: 86 %; 95 % purity. White solid. ^{1}H NMR (500 MHz, $CDCl_3$) δ 7.67 – 7.57 (m, 2H), 7.51 (ddd, J = 14.5, 8.3, 4.6 Hz, 3H), 3.98 – 3.79 (m, 4H), 3.73 (s, 4H). ^{13}C NMR (126 MHz, $CDCl_3$) δ 173.43, 163.06, 152.62, 138.43, 130.95, 129.35, 129.05, 128.84, 126.14, 114.14, 65.95, 48.65. HRMS (ESI) *m/z*: $[M+H]^+$Calcd for $C_{14}H_{14}N_5O_2S^+$ 316.0863; Found 316.0862

6-(diethylamino)-3-phenylthiazolo[4,5-*d*][1,2,3]triazin-4(3*H*)-one (1bf).

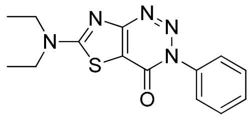

1bf

Isolated yield: 53 %; 95 % purity. Brown solid. ^{1}H NMR (500 MHz, $CDCl_3$) δ 7.64 – 7.57 (m, 2H), 7.55 – 7.43 (m, 3H), 3.66 (s, 4H), 1.33 (t, J = 7.2 Hz, 6H). ^{13}C NMR (126 MHz, $CDCl_3$) δ 172.05, 163.55, 152.73, 138.58, 129.22, 129.02, 126.19, 113.55, 12.39. HRMS (ESI) *m/z*: $[M+H]^+$Calcd for $C_{14}H_{16}N_5OS^+$302.1070; Found 302.1070

6-((4-methoxybenzyl)amino)-3-phenylthiazolo[4,5-*d*][1,2,3]triazin-4(3*H*)-one (1bg).

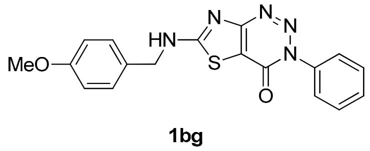

1bg

Isolated yield: 74 %; 95 % purity. Ivory solid. ^{1}H NMR (500 MHz, $CDCl_3$) δ 8.24 (s, 1H), 7.65 – 7.56 (m, 2H), 7.56 – 7.45 (m, 3H), 7.33 (d, J = 8.6 Hz, 2H), 6.88 (d, J = 8.7 Hz, 2H), 4.60 (d, J = 5.3 Hz, 2H), 3.79 (s, 3H). ^{13}C NMR (126 MHz, $CDCl_3$) δ 162.61, 159.65, 152.76, 138.43, 129.52, 129.38, 129.09, 126.14, 114.36,

55.35, 49.26. HRMS (ESI) *m/z*: [M+H] $^+$Calcd for $C_{18}H_{16}N_5O_2S^+$ 366.1019; Found 366.1020

3-phenyl-6-(propylthio)thiazolo[4,5-*d*][1,2,3]triazin-4(3*H*)-one (1bh).

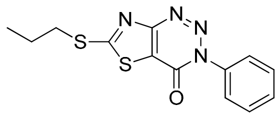

1bh

Isolated yield: 51 %; 95 % purity. Pink solid. ^{1}H NMR (500 MHz, $CDCl_3$) δ 7.66 – 7.58 (m, 2H), 7.58 – 7.48 (m, 3H), 3.44 (t, J = 7.2 Hz, 2H), 1.96 – 1.85 (m, 2H), 1.10 (t, J = 7.4 Hz, 3H). ^{13}C NMR (126 MHz, $CDCl_3$) δ 178.46, 162.51, 152.33, 138.08, 129.60, 129.18, 126.13, 122.21, 36.23, 22.37, 13.35. HRMS (ESI) *m/z*: [M+H] $^+$Calcd for $C_{13}H_{13}N_4OS_2^+$ 305.0525; Found 305.0525

6-(benzylthio)-3-phenylthiazolo[4,5-*d*][1,2,3]triazin-4(3*H*)-one (1bi).

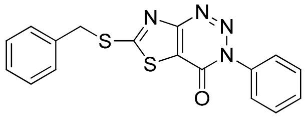

1bi

Isolated yield: 30 %; 95 % purity. White-brown solid. ^{1}H NMR (500 MHz, $CDCl_3$) δ 7.62 (dt, J = 3.7, 2.1 Hz, 2H), 7.54 (dddd, J = 11.2, 5.6, 3.0, 1.4 Hz, 3H), 7.51 – 7.44 (m, 2H), 7.41 – 7.29 (m, 3H), 4.71 (s, 2H). ^{13}C NMR (126 MHz, $CDCl_3$) δ 177.39, 162.37, 152.38, 138.06, 134.82, 129.64, 129.30, 129.21, 128.97, 128.32, 126.12, 122.55, 38.35. HRMS (ESI) *m/z*: [M+H] $^+$Calcd for $C_{17}H_{13}N_4OS_2^+$ 353.0525; Found 353.0525

3-phenyl-6-(phenylthio)thiazolo[4,5-*d*][1,2,3]triazin-4(3*H*)-one (1bj).

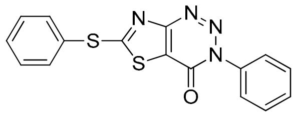

1bj

Isolated yield: 64 %; 95 % purity. Brown solid. 1H NMR (500 MHz, CDCl3) δ 7.77 (dt, J = 3.5, 2.5 Hz, 2H), 7.66 – 7.61 (m, 1H), 7.60 – 7.46 (m, 7H). 13C NMR (126 MHz, CDCl3) δ 182.41, 163.21, 152.09, 138.06, 135.84, 132.05, 130.95, 129.58, 129.17, 127.89, 126.07, 122.48. HRMS (ESI) m/z: [M+H] +Calcd for $C_{16}H_{11}N_4OS_2^+$339.0369 ; Found 339.0369

6-(butylamino)-3-(p-tolyl)thiazolo[4,5-*d*][1,2,3]triazin-4(3*H*)-one (1ca).

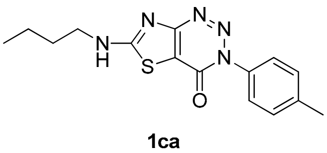

1ca

Isolated yield: 70 %; 95 % purity. White solid. ^{1}H NMR (500 MHz, $CDCl_3$) δ 7.97 (s, 1H), 7.48 (d, J = 6.8 Hz, 2H), 7.34 (d, J = 6.8 Hz, 2H), 3.46 (s, 2H), 2.44 (s, 3H), 1.79 (s, 2H), 1.46 (d, J = 6.4 Hz, 2H), 0.97 (s, 3H). ^{13}C NMR (126 MHz, $CDCl_3$) δ 157.22, 145.65, 137.02, 132.45, 132.45, 129.69, 125.91, 105.20, 40.83, 30.73, 21.32, 20.07, 13.72. HRMS (ESI) *m/z*: [M+H] $^+$Calcd for $C_{15}H_{18}N_5OS^+$ 316.1227; Found 316.1226

6-(piperidin-1-yl)-3-(p-tolyl)thiazolo[4,5-*d*][1,2,3]triazin-4(3*H*)-one (1cb).

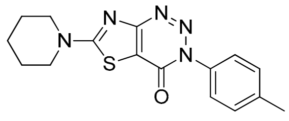

1cb

Isolated yield: 79 %; 95 % purity. Pink solid. ^{1}H NMR (500 MHz, $CDCl_3$) δ 7.47 (d, J = 8.4 Hz, 2H), 7.31 (d, J = 8.2 Hz, 2H), 3.70 (s, 4H), 2.41 (s, 3H), 1.73 (s, 6H). ^{13}C NMR (126 MHz, $CDCl_3$) δ 172.90, 163.53, 152.78, 139.35, 136.04, 129.61, 125.92, 113.59, 50.14, 25.24, 23.85, 21.32. HRMS (ESI) *m/z*: [M+H] $^+$Calcd for $C_{16}H_{18}N_5OS^+$ 328.1227; Found 328.1225

6-(benzylamino)-3-(p-tolyl)thiazolo[4,5-*d*][1,2,3]triazin-4(3*H*)-one (1cc).

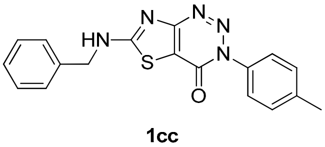

1cc

Isolated yield: 87 %; 95 % purity. Brown solid. ^{1}H NMR (500 MHz, $CDCl_3$) δ 8.84 (s, 1H), 7.44 (d, J = 8.3 Hz, 2H), 7.39 (d, J = 7.2 Hz, 2H), 7.31 (tt, J = 7.2, 5.4 Hz, 5H), 4.66 (d, J = 5.6 Hz, 2H), 2.42 (s, 3H). ^{13}C NMR (126 MHz, $CDCl_3$) δ 162.65, 152.90, 139.61, 135.86, 129.72, 128.94, 128.24, 128.00, 125.90, 49.57, 21.33. HRMS (ESI) *m/z*: [M+H] $^+$Calcd for $C_{18}H_{16}N_5OS^+$ 350.1070; Found 350.1069

6-(pyrrolidin-1-yl)-3-(p-tolyl)thiazolo[4,5-*d*][1,2,3]triazin-4(3*H*)-one (1cd).

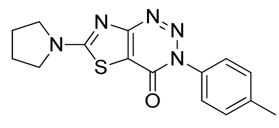

1cd

Isolated yield: 69 %; 95 % purity. Pink solid. ^{1}H NMR (500 MHz, $CDCl_3$) δ 7.47 (t, J = 5.2 Hz, 2H), 7.31 (d, J = 8.3 Hz, 2H), 3.63 (d, J = 213.7 Hz, 4H), 2.42 (s, 3H), 2.14 (s, 4H). ^{13}C NMR (126 MHz, $CDCl_3$) δ 169.60, 163.49, 152.90, 139.34, 136.03, 129.62, 125.94, 113.72, 50.31, 25.71, 21.32. HRMS (ESI) *m/z*: [M+H]$^+$Calcd for $C_{15}H_{16}N_5OS^+$314.1070; Found 314.1069

6-morpholino-3-(p-tolyl)thiazolo[4,5-*d*][1,2,3]triazin-4(3*H*)-one (1ce).

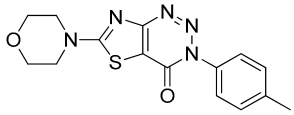

1ce

Isolated yield: 65 %; 95 % purity. White solid. ^{1}H NMR (500 MHz, $CDCl_3$) δ 7.48 (d, J = 8.4 Hz, 2H), 7.33 (d, J = 8.1 Hz, 2H), 3.90 – 3.82 (m, 4H), 3.74 (s, 4H), 2.43 (s, 3H). ^{13}C NMR (126 MHz, $CDCl_3$) δ 173.40, 163.08, 152.73, 139.53, 135.90, 129.67, 125.90, 114.14, 65.98, 48.63, 21.32. HRMS (ESI) *m/z*: [M+H]$^+$Calcd for $C_{15}H_{16}N_5O_2S_2^+$ 330.1019; Found 330.1020

6-(diethylamino)-3-(p-tolyl)thiazolo[4,5-*d*][1,2,3]triazin-4(3*H*)-one (1cf).

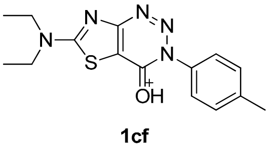

1cf

Isolated yield: 55 %; 95 % purity. Yellow solid. ^{1}H NMR (500 MHz, $CDCl_3$) δ 7.50 – 7.44 (m, 2H), 7.32 (d, J = 8.1 Hz, 2H), 3.64 (s, 4H), 2.42 (s, 3H), 1.32 (t, J = 7.2 Hz, 6H). ^{13}C NMR (126 MHz, $CDCl_3$) δ 171.99, 163.56, 152.82, 139.36, 136.04,

129.63, 125.95, 113.52, 21.31, 12.41. HRMS (ESI) *m/z*: [M+H] $^+$Calcd for $C_{15}H_{18}N_5OS^+$316.1227; Found 316.1227

6-((4-methoxybenzyl)amino)-3-(p-tolyl)thiazolo[4,5-*d*][1,2,3]triazin-4(3*H*)-one (1cg).

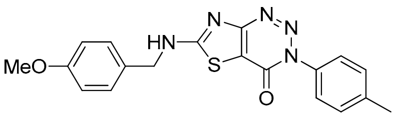

1cg

Isolated yield: 73 %; 95 % purity. White solid. ^{1}H NMR (500 MHz, $CDCl_3$) δ 8.38 (s, 1H), 7.46 (d, J = 8.3 Hz, 2H), 7.36 – 7.29 (m, 4H), 6.90 – 6.83 (m, 2H), 4.60 (d, J = 5.5 Hz, 2H), 3.79 (s, 3H), 2.43 (s, 3H). ^{13}C NMR (126 MHz, $CDCl_3$) δ 162.62, 159.60, 152.85, 139.56, 135.90, 129.70, 129.53, 125.89, 114.32, 55.35, 49.21, 21.33. HRMS (ESI) *m/z*: [M+H] $^+$Calcd for $C_{19}H_{18}N_5O_2S^+$ 380.1176; Found 380.1176

6-(propylthio)-3-(p-tolyl)thiazolo[4,5-*d*][1,2,3]triazin-4(3*H*)-one (1ch).

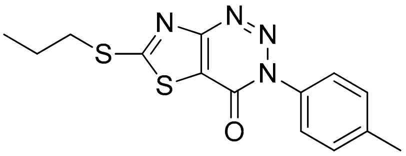

1ch

Isolated yield: 56 %, 95 % purity. Pink solid. ^{1}H NMR (500 MHz, $CDCl_3$) δ 7.52 – 7.45 (m, 2H), 7.35 (d, J = 8.1 Hz, 2H), 3.46 – 3.40 (m, 2H), 2.44 (s, 3H), 1.94 – 1.85 (m, 2H), 1.10 (t, J = 7.4 Hz, 3H). ^{13}C NMR (126 MHz, $CDCl_3$) δ 178.35, 162.53, 152.43, 139.84, 135.54, 129.80, 125.88, 122.15, 36.20, 22.37, 21.35, 13.38. HRMS (ESI) *m/z*: [M+H] $^+$Calcd for $C_{14}H_{15}N_4OS_2^+$ 319.0682; Found 319.0683

6-(benzylthio)-3-(p-tolyl)thiazolo[4,5-*d*][1,2,3]triazin-4(3*H*)-one (1ci).

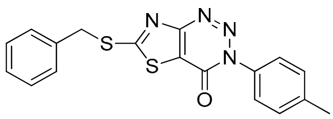

1ci

Isolated yield: 46 %, 95 % purity. White-brown solid. ^{1}H NMR (500 MHz, $CDCl_3$) δ 7.51 – 7.46 (m, 4H), 7.39 – 7.30 (m, 5H), 4.70 (s, 2H), 2.45 (s, 3H). ^{13}C NMR (126 MHz, $CDCl_3$) δ 177.25, 162.35, 152.43, 139.88, 135.50, 134.83, 129.81, 129.30, 128.96, 128.30, 125.87, 122.44, 38.31, 21.36. HRMS (ESI) *m/z*: $[M+H]^+$Calcd for $C_{18}H_{15}N_4OS_2^+$ 367.0682; Found 367.0683

6-(phenylthio)-3-(p-tolyl)thiazolo[4,5-*d*][1,2,3]triazin-4(3*H*)-one (1cj)

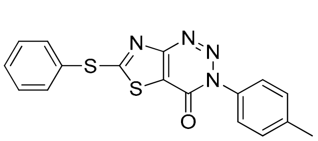

1cj

Isolated yield: 67 %; 95 % purity. Gray solid. ^{1}H NMR (500 MHz, $CDCl_3$) δ 7.76 (dt, J = 3.6, 2.5 Hz, 2H), 7.65 – 7.60 (m, 1H), 7.60 – 7.54 (m, 2H), 7.47 – 7.43 (m, 2H), 7.35 – 7.30 (m, 2H), 2.42 (s, 3H). ^{13}C NMR (126 MHz, $CDCl_3$) δ 182.34, 163.23, 152.17, 139.80, 135.86, 135.52, 132.05, 130.95, 129.78, 127.87, 125.84, 122.41, 21.35. HRMS (ESI) *m/z*: $[M+H]^+$Calcd for $C_{17}H_{13}N_4OS_2^+$353.0525; Found 353.0525

6-(butylamino)-3-(4-nitrophenyl)thiazolo[4,5-*d*][1,2,3]triazin-4(3*H*)-one (1da).

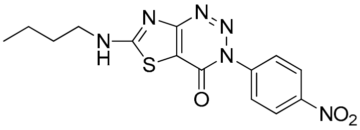

1da

Isolated yield: 34 %; 95 % purity. White solid. ^{1}H NMR (500 MHz, DMSO) δ 9.22 (s, 1H), 8.48 – 8.38 (m, 2H), 8.01 – 7.90 (m, 2H), 1.64 – 1.54 (m, 2H), 1.42 – 1.33 (m, 2H), 0.92 (t, J = 7.4 Hz, 3H). ^{13}C NMR (126 MHz, DMSO) δ 162.76, 152.03,

147.37, 143.65, 128.73, 127.86, 127.69, 124.31, 30.37, 19.56, 13.68. HRMS (ESI) *m/z*: [M+H] $^+$Calcd for $C_{14}H_{15}N_6O_3S^+$ 347.0921; Found 349.0921

3-(4-nitrophenyl)-6-(piperidin-1-yl)thiazolo[4,5-*d*][1,2,3]triazin-4(3*H*)-one (1db).

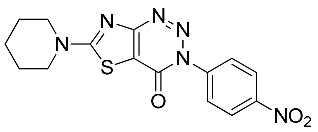

1db

Isolated yield: 42 %; 95 % purity. White yellow solid. ^{1}H NMR (500 MHz, $CDCl_3$) δ 8.39 (d, J = 9.0 Hz, 2H), 7.92 (d, J = 9.0 Hz, 2H), 3.73 (s, 4H), 1.76 (s, 6H). ^{13}C NMR (126 MHz, $CDCl_3$) δ 173.09, 163.27, 152.28, 147.50, 143.56, 126.75, 124.31, 113.45, 25.26, 23.80. HRMS (ESI) *m/z*: [M+H] $^+$Calcd for $C_{15}H_{15}N_6O_3S^+$ 359.0921; Found 359.0920

6-(benzylamino)-3-(4-nitrophenyl)thiazolo[4,5-*d*][1,2,3]triazin-4(3*H*)-one (1dc).

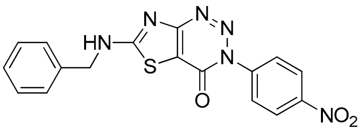

1dc

Isolated yield: 39 %; 95 % purity. Ivory solid. ^{1}H NMR (500 MHz, DMSO) δ 9.69 (s, 1H), 8.47 – 8.36 (m, 2H), 8.00 – 7.87 (m, 2H), 7.46 – 7.35 (m, 4H), 7.35 – 7.27 (m, 1H), 4.70 (s, 2H). ^{13}C NMR (126 MHz, DMSO) δ 162.90, 152.47, 147.77, 144.02, 129.09, 128.28, 128.12, 128.02, 124.71. HRMS (ESI) *m/z*: [M+H] $^+$Calcd for $C_{17}H_{13}N_6O_3S^+$ 381.0764; Found 381.0764

3-(4-nitrophenyl)-6-(pyrrolidin-1-yl)thiazolo[4,5-*d*][1,2,3]triazin-4(3*H*)-one (1dd).

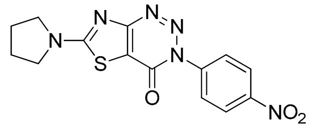

1dd

Isolated yield: 27 %; 95 % purity. Brown solid. ^{1}H NMR (500 MHz, $CDCl_3$) δ 8.40 (d, J = 9.1 Hz, 2H), 7.97 – 7.89 (m, 2H), 3.67 (d, J = 216.0 Hz, 4H), 2.18 (d, J = 5.8 Hz, 4H). ^{13}C NMR (126 MHz, $CDCl_3$) δ 169.88, 163.19, 152.38, 147.55, 143.55, 126.76, 124.31, 49.96, 25.86. HRMS (ESI) *m/z*: [M+H] $^+$Calcd for $C_{14}H_{13}N_6O_3S^+$ 345.0764; Found 345.0764

6-morpholino-3-(4-nitrophenyl)thiazolo[4,5-*d*][1,2,3]triazin-4(3*H*)-one (1de).

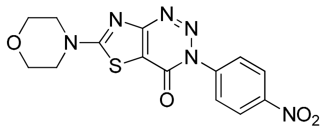

1de

Isolated yield: 22 %; 95 % purity. Ivory solid. ^{1}H NMR (500 MHz, $CDCl_3$) δ 8.44 – 8.37 (m, 2H), 7.96 – 7.89 (m, 2H), 3.94 – 3.84 (m, 4H), 3.78 (s, 4H). ^{13}C NMR (126 MHz, $CDCl_3$) δ 173.62, 162.87, 152.25, 147.62, 143.37, 126.68, 124.33, 114.06, 65.98, 53.35, 48.77, 29.68. HRMS (ESI) *m/z*: [M+H] $^+$Calcd for $C_{14}H_{13}N_6O_4S^+$361.0714 Found 361.0712

6-(diethylamino)-3-(4-nitrophenyl)thiazolo[4,5-*d*][1,2,3]triazin-4(3*H*)-one (1df).

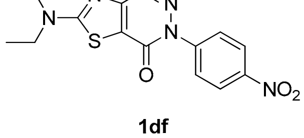

1df

Isolated yield: 54 %; 95 % purity. Yellow solid. ^{1}H NMR (500 MHz, $CDCl_3$) δ 8.43 – 8.35 (m, 2H), 7.95 – 7.88 (m, 2H), 3.66 (s, 4H), 1.39 – 1.29 (m, 6H). ^{13}C NMR (126 MHz, $CDCl_3$) δ 172.26, 163.27, 152.30, 147.49, 143.57, 126.78, 124.32, 113.39. HRMS (ESI) *m/z*: [M+H] $^+$Calcd for $C_{14}H_{15}N_6O_3S^+$ 347.0921; Found 347.0921

6-((4-methoxybenzyl)amino)-3-(4-nitrophenyl)thiazolo[4,5-*d*][1,2,3]triazin-4(3*H*)-one (1dg).

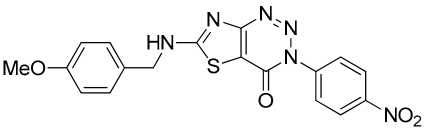

1dg

Isolated yield: 47 %; 95 % purity. Ivory solid. ^{1}H NMR (500 MHz, DMSO) δ 9.59 (s, 1H), 8.45 – 8.38 (m, 2H), 7.98 – 7.90 (m, 2H), 7.33 (d, J = 8.7 Hz, 2H), 6.97 – 6.90 (m, 2H), 4.60 (s, 2H), 3.74 (s, 3H). ^{13}C NMR (126 MHz, DMSO) δ 162.93, 159.19, 152.45, 147.75, 144.02, 129.65, 128.26, 124.70, 114.41, 55.57. HRMS (ESI) *m/z*: [M+H] $^+$Calcd for $C_{18}H_{15}N_6O_4S^+$ 411.0870; Found 411.0866

3-(4-nitrophenyl)-6-(propylthio)thiazolo[4,5-*d*][1,2,3]triazin-4(3*H*)-one (1dh).

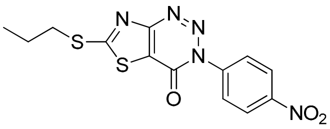

1dh

Isolated yield: 34 %; 95 % purity. White solid. ^{1}H NMR (500 MHz, $CDCl_3$) δ 8.45 – 8.39 (m, 2H), 7.95 – 7.88 (m, 2H), 3.46 (t, J = 7.2 Hz, 2H), 1.91 (h, J = 7.3 Hz, 2H), 1.11 (t, J = 7.4 Hz, 3H). ^{13}C NMR (126 MHz, $CDCl_3$) δ 179.32, 162.20, 151.96, 147.79, 142.99, 126.83, 124.49, 122.28, 36.37, 22.33, 13.35. HRMS (ESI) *m/z*: [M+H] $^+$Calcd for $C_{13}H_{12}N_5O_3S_2^+$ 350.0376; Found 350.0376

6-(benzylthio)-3-(4-nitrophenyl)thiazolo[4,5-*d*][1,2,3]triazin-4(3*H*)-one (1di).

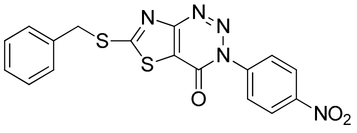

1di

Isolated yield: 38 %; 95 % purity. White solid. ^{1}H NMR (500 MHz, $CDCl_3$) δ 8.43 (dd, J = 9.1, 2.1 Hz, 2H), 7.92 (dd, J = 9.1, 2.1 Hz, 2H), 7.55 – 7.31 (m, 5H), 4.72 (s, 2H). ^{13}C NMR (126 MHz, $CDCl_3$) δ 178.16, 162.06, 151.99, 147.89, 143.00, 134.64, 129.29, 129.01, 128.38, 126.81, 124.50, 122.60, 38.46. HRMS (ESI) *m/z*: [M+H] $^{+}$Calcd for $C_{17}H_{12}N_5O_3S_2^{+}$ 398.0376; Found 398.0374

3-(4-nitrophenyl)-6-(phenylthio)thiazolo[4,5-*d*][1,2,3]triazin-4(3*H*)-one (1dj).

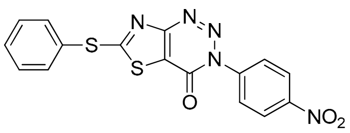

1dj

Isolated yield: 33 %; 95 % purity. Ivory solid. ^{1}H NMR (500 MHz, $CDCl_3$) δ 8.42 – 8.37 (m, 2H), 7.91 – 7.86 (m, 2H), 7.79 – 7.74 (m, 2H), 7.68 – 7.63 (m, 1H), 7.63 – 7.56 (m, 2H). ^{13}C NMR (126 MHz, $CDCl_3$) δ 183.43, 162.89, 151.71, 147.75, 142.96, 135.82, 132.24, 131.08, 127.67, 126.77, 124.47, 122.49. HRMS (ESI) *m/z*: [M+H] $^{+}$Calcd for $C_{16}H_{10}N_5O_3S_2^{+}$ 383.0220; Found 383.0218

1.3 NMR spectra

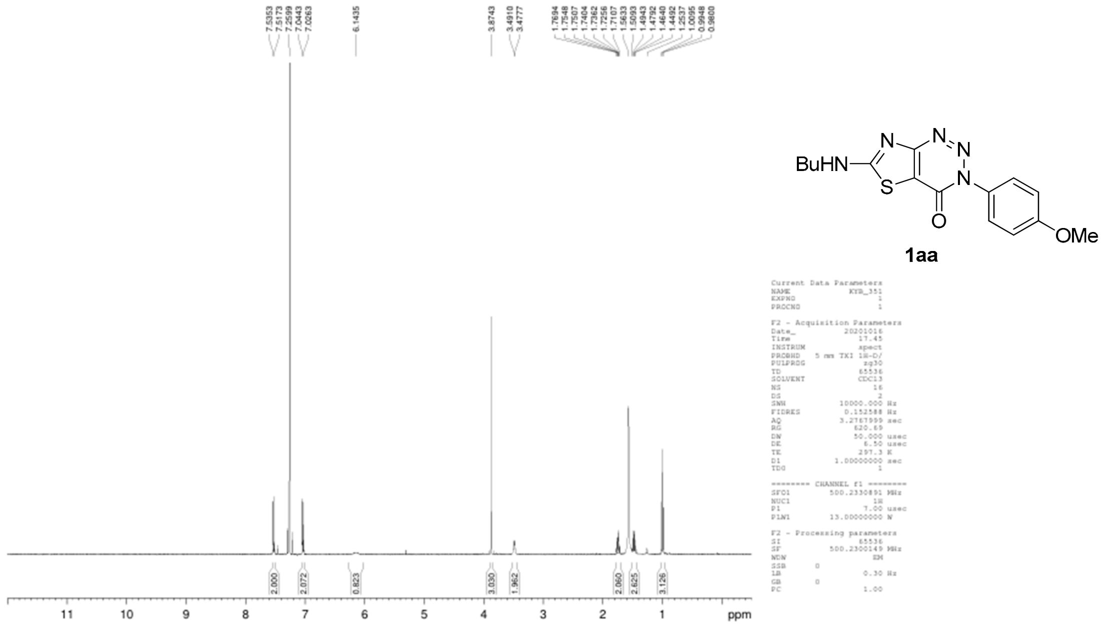

1aa

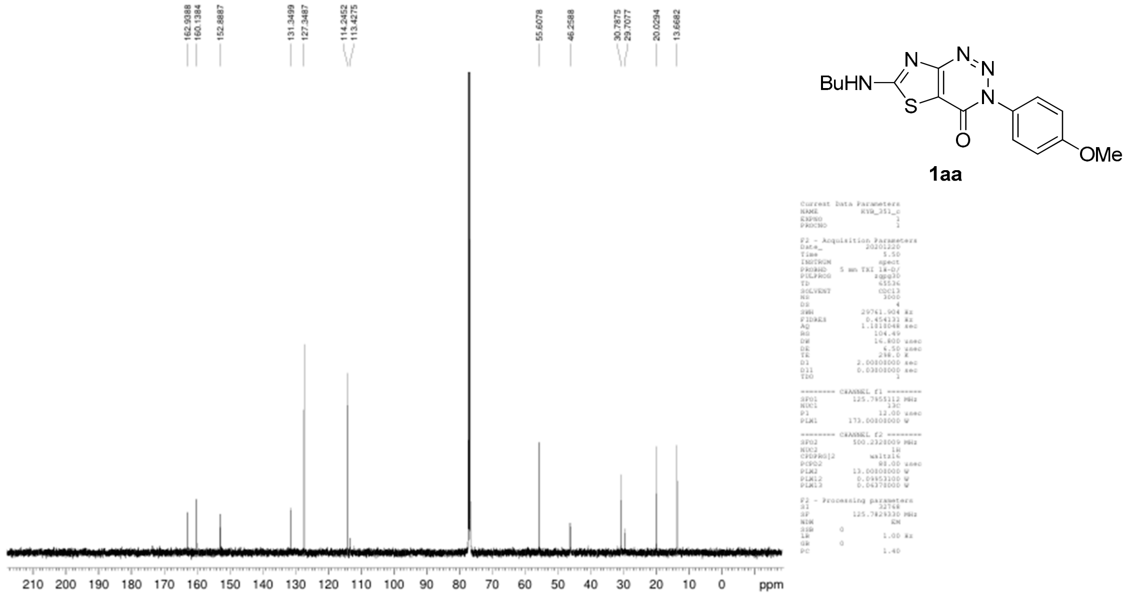

1aa

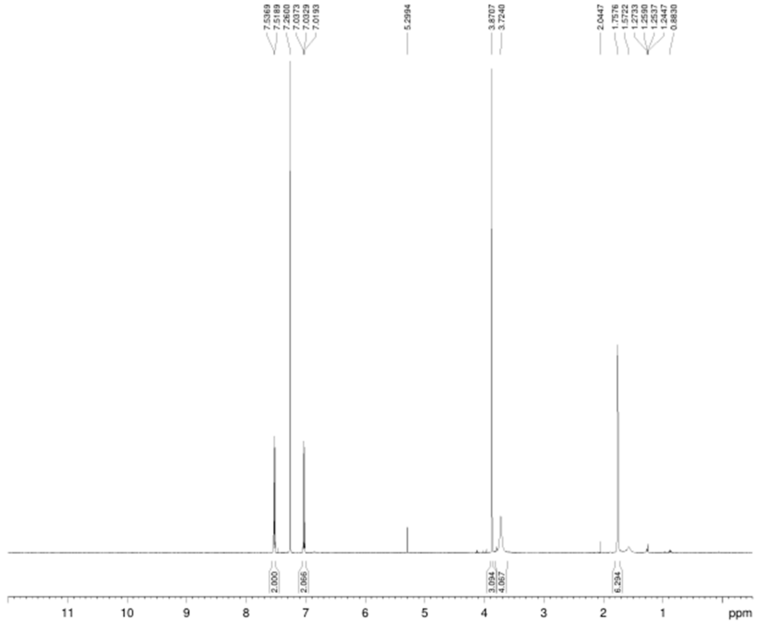

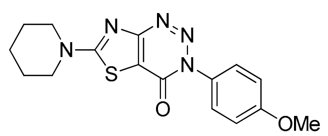

1ab

```
Current Data Parameters
NAME              KYB_356
EXPNO                   1
PROCNO                  1

F2 - Acquisition Parameters
Date_            20201023
Time                17.31
INSTRUM             spect
PROBHD   5 mm TXI 1H-D/
PULPROG              zg30
TD                  65536
SOLVENT             CDC13
NS                     16
DS                      2
SWH          10000.000 Hz
FIDRES        0.152588 Hz
AQ           3.2767999 sec
RG                 549.91
DW              50.000 usec
DE               6.50 usec
TE                297.7 K
D1          1.00000000 sec
TD0                     1

======== CHANNEL f1 ========
SFO1       500.2330891 MHz
NUC1                   1H
P1               7.00 usec
PLW1       13.00000000 W

F2 - Processing parameters
SI                  65536
SF         500.2300149 MHz
WDW                    EM
SSB       0
LB                0.30 Hz
GB        0
PC                   1.00
```

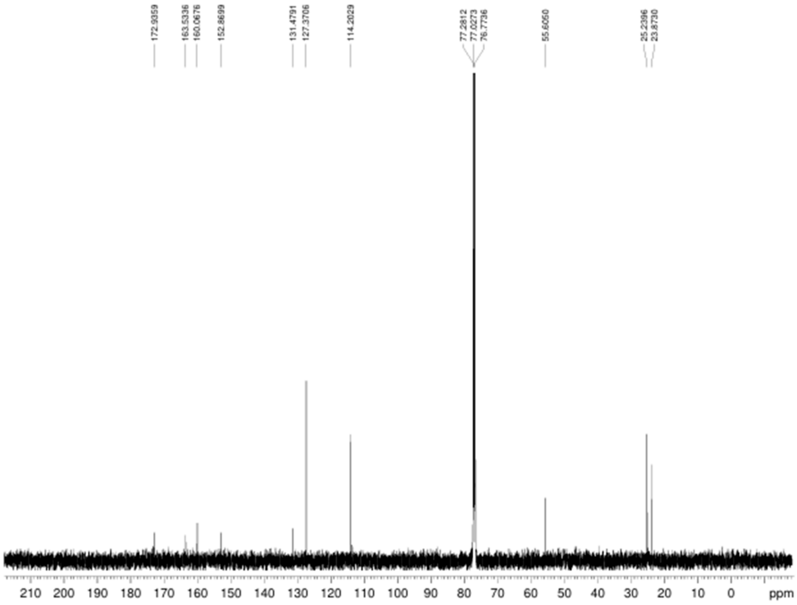

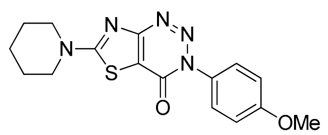

1ab

```
Current Data Parameters
NAME              KYB_356
EXPNO                   2
PROCNO                  1

F2 - Acquisition Parameters
Date_            20201025
Time                 5.13
INSTRUM             spect
PROBHD   5 mm TXI 1H-D/
PULPROG           zgpg30
TD                  65536
SOLVENT             CDC13
NS                   6000
DS                      4
SWH          29761.904 Hz
FIDRES        0.454131 Hz
AQ           1.1010048 sec
RG                 128.14
DW              16.800 usec
DE               6.50 usec
TE                297.5 K
D1          2.00000000 sec
D11         0.03000000 sec
TD0                     1

======== CHANNEL f1 ========
SFO1       125.7955112 MHz
NUC1                  13C
P1              12.00 usec
PLW1      173.00000000 W

======== CHANNEL f2 ========
SFO2       500.2320009 MHz
NUC2                   1H
CPDPRG[2          waltz16
PCPD2           80.00 usec
PLW2       13.00000000 W
PLW12       0.09953100 W
PLW13       0.06378000 W

F2 - Processing parameters
SI                  32768
SF         125.7829330 MHz
WDW                    EM
SSB       0
LB                1.00 Hz
GB        0
PC                   1.40
```

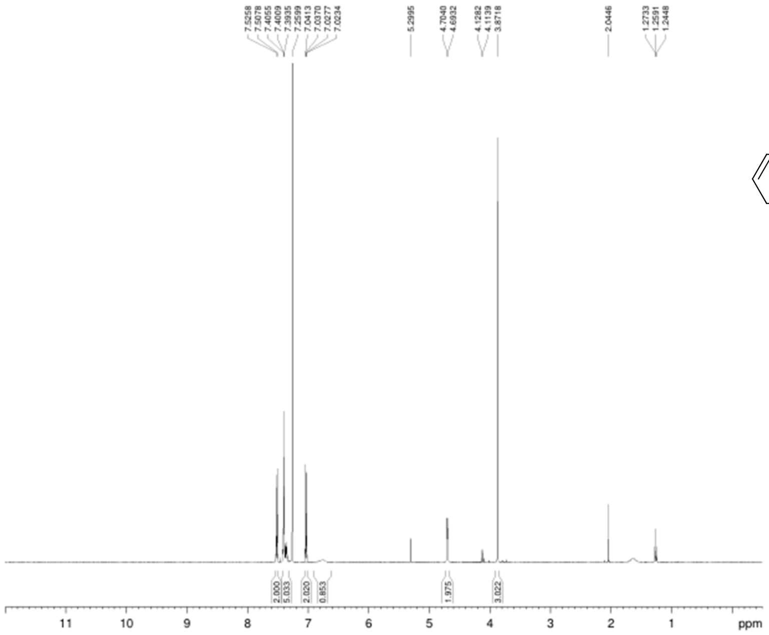
7.5258
7.5078
7.4055
7.4009
7.3935
7.2599
7.0413
7.0370
7.0277
7.0234
5.2995
4.7040
4.6932
4.1282
4.1139
3.8718
2.0446
1.2733
1.2591
1.2448
2.000
5.033
2.020
0.853
1.975
3.022
11 10 9 8 7 6 5 4 3 2 1 ppm

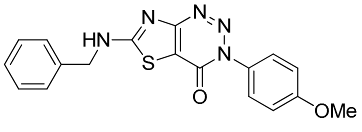
N
N
N
HN
S
N
O
OMe

1ac

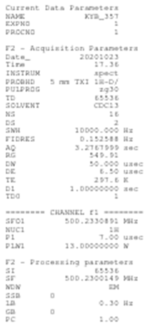

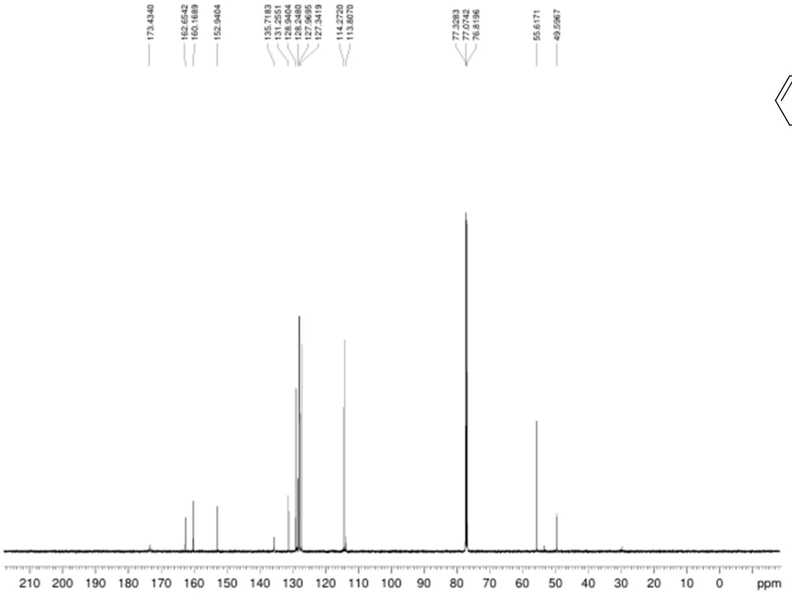
173.4340
162.6542
160.1689
152.9404
135.7183
131.2551
128.9404
128.2480
127.9695
127.3419
114.2720
113.8070
77.3283
77.0742
76.8196
55.6171
49.5967
210 200 190 180 170 160 150 140 130 120 110 100 90 80 70 60 50 40 30 20 10 0 ppm

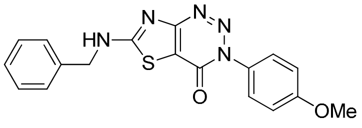
N
N
N
HN
S
N
O
OMe

1ac

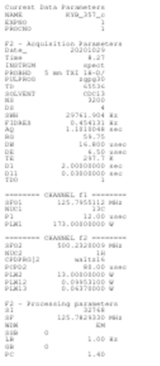

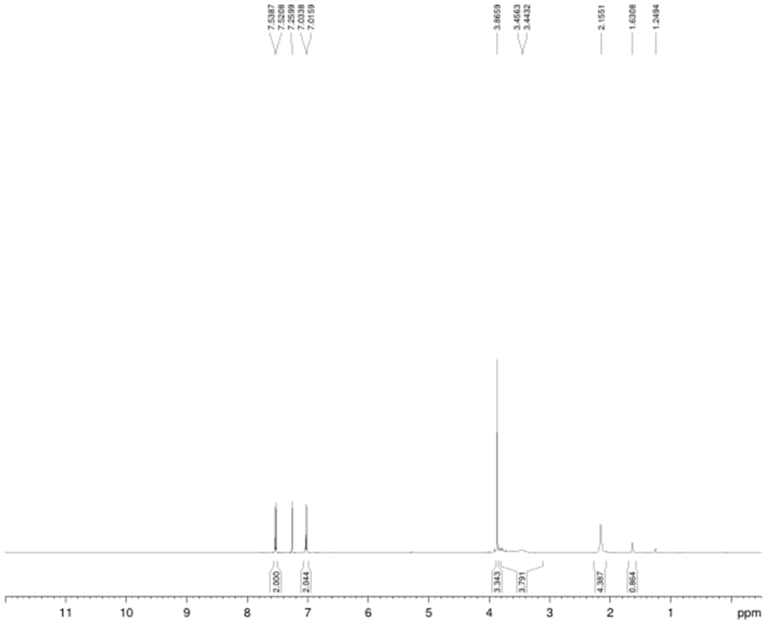
7.5387
7.5208
7.2599
7.0338
7.0159
3.8659
3.4563
3.4432
2.1551
1.6308
1.2494
2.000
2.044
3.343
3.791
4.387
0.864
11 10 9 8 7 6 5 4 3 2 1 ppm

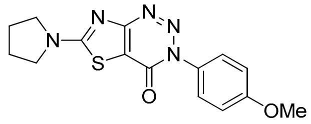
N
N
N
N
S
O
OMe

1ad

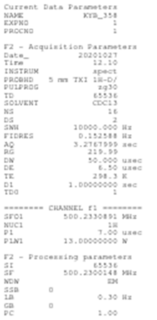

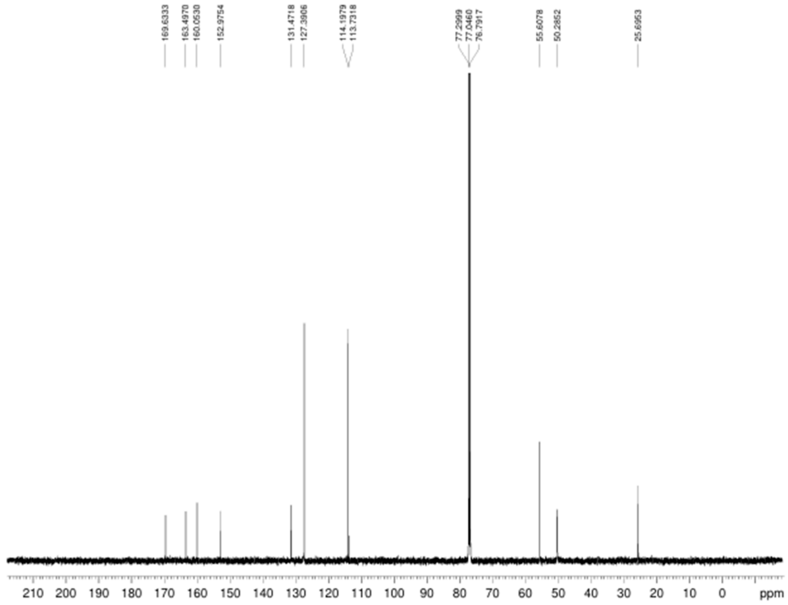
169.6333
163.4970
160.0530
152.9754
131.4718
127.3906
114.1979
113.7318
77.2999
77.0460
76.7917
55.6078
50.2852
25.6953
210 200 190 180 170 160 150 140 130 120 110 100 90 80 70 60 50 40 30 20 10 0 ppm

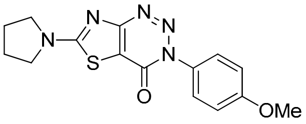
N
N
N
N
S
O
OMe

1ad

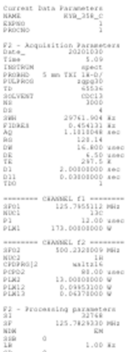

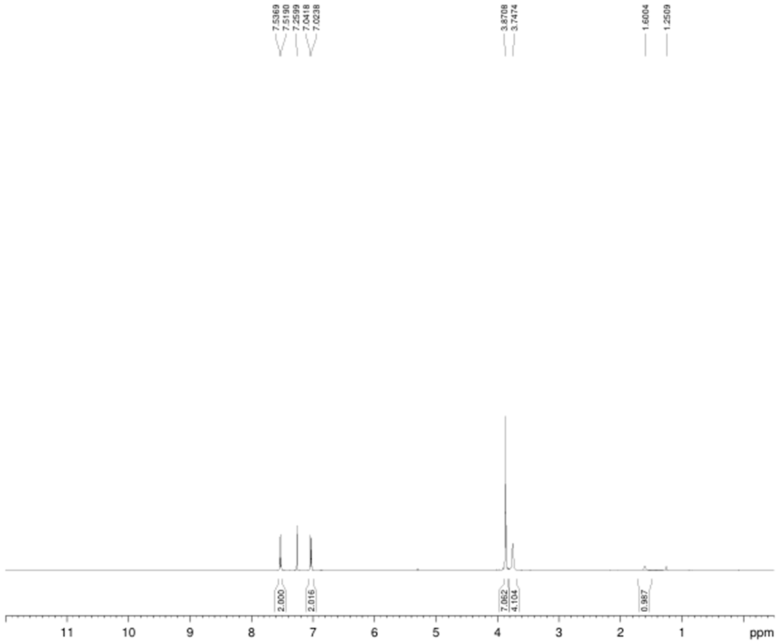

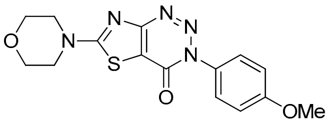

1ae

Current Data Parameters
NAME KYR_359
EXPNO 1
PROCNO 1

F2 - Acquisition Parameters
Date_ 20201027
Time 12.15
INSTRUM spect
PROBHD 5 mm TXI 1H-D/
PULPROG zg30
TD 65536
SOLVENT CDCl3
NS 16
DS 2
SWH 10000.000 Hz
FIDRES 0.152588 Hz
AQ 3.2767999 sec
RG 192.71
DW 50.000 usec
DE 6.50 usec
TE 298.3 K
D1 1.00000000 sec
TD0 1

======== CHANNEL f1 ========
SFO1 500.2330891 MHz
NUC1 1H
P1 7.00 usec
PLW1 13.00000000 W

F2 - Processing parameters
SI 65536
SF 500.2300147 MHz
WDW EM
SSB 0
LB 0.30 Hz
GB 0
PC 1.00

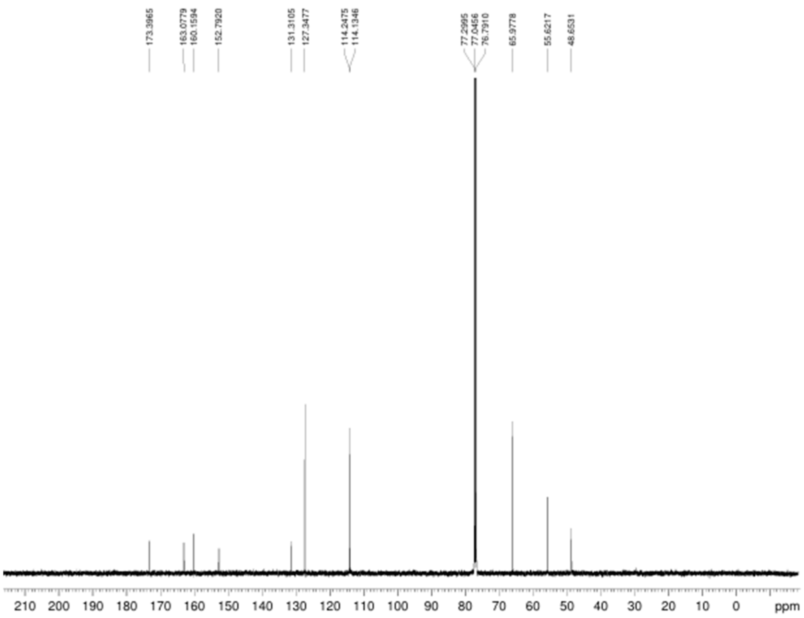

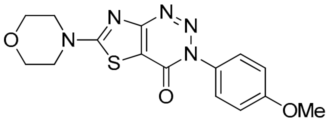

1ae

Current Data Parameters
NAME KYR_359_C
EXPNO 1
PROCNO 1

F2 - Acquisition Parameters
Date_ 20201030
Time 8.02
INSTRUM spect
PROBHD 5 mm TXI 1H-D/
PULPROG zgpg30
TD 65536
SOLVENT CDCl3
NS 3200
DS 4
SWH 29761.904 Hz
FIDRES 0.454131 Hz
AQ 1.1010048 sec
RG 59.75
DW 16.800 usec
DE 6.50 usec
TE 297.5 K
D1 2.00000000 sec
D11 0.03000000 sec
TD0 1

======== CHANNEL f1 ========
SFO1 125.7955112 MHz
NUC1 13C
P1 12.00 usec
PLW1 173.00000000 W

======== CHANNEL f2 ========
SFO2 500.2320009 MHz
NUC2 1H
CPDPRG[2 waltz16
PCPD2 80.00 usec
PLW2 13.00000000 W
PLW12 0.09953100 W
PLW13 0.06378000 W

F2 - Processing parameters
SI 32768
SF 125.7829330 MHz
WDW EM
SSB 0
LB 1.00 Hz
GB 0
PC 1.40

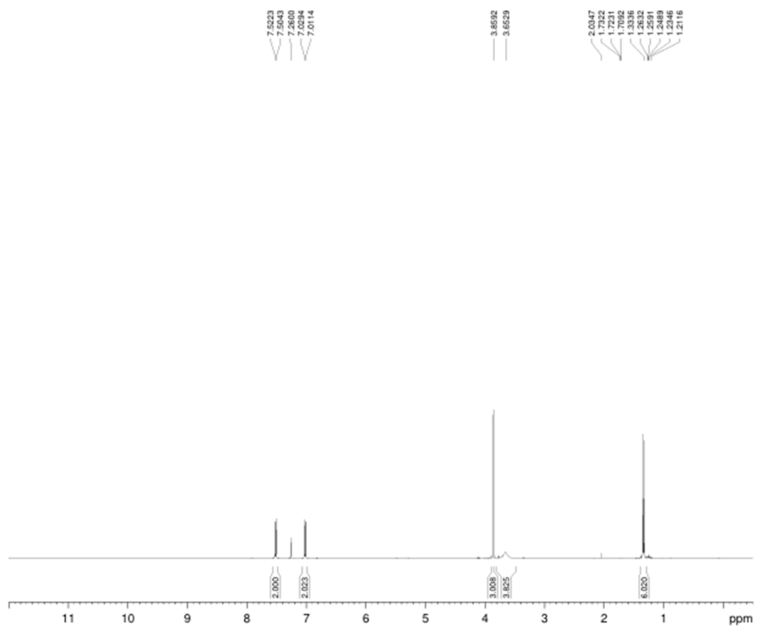
7.5223
7.5043
7.2600
7.0294
7.0114
3.8592
3.6529
2.0347
1.7322
1.7231
1.7092
1.3336
1.2632
1.2591
1.2489
1.2346
1.2116
2.000
2.023
3.008
3.825
6.020
11
10
9
8
7
6
5
4
3
2
1
ppm

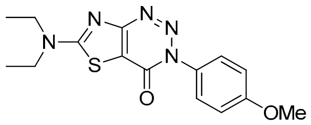
N
N
N
N
S
O
OMe

1af

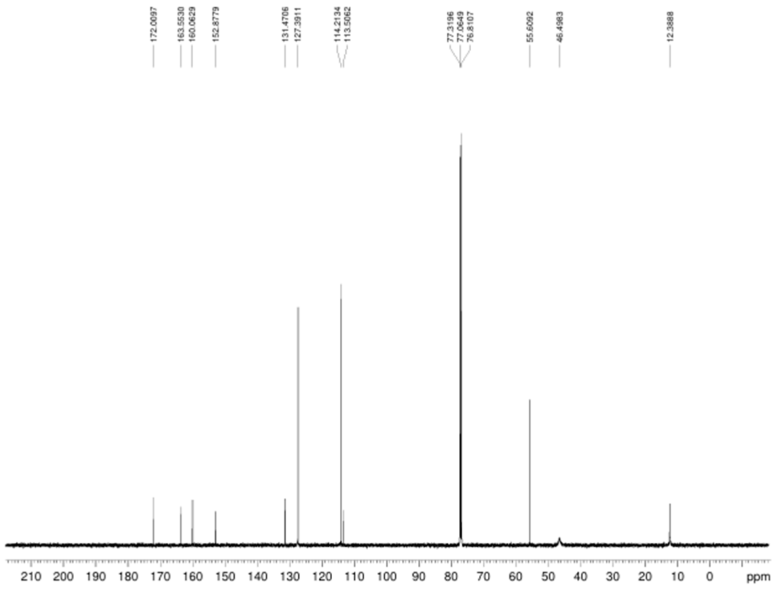
172.0097
163.5530
160.0629
152.8779
131.4706
127.3911
114.2134
113.5062
77.3196
77.0649
76.8107
55.6092
46.4983
12.3888
210
200
190
180
170
160
150
140
130
120
110
100
90
80
70
60
50
40
30
20
10
0
ppm

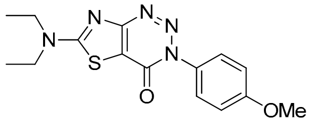
N
N
N
N
S
O
OMe

1af

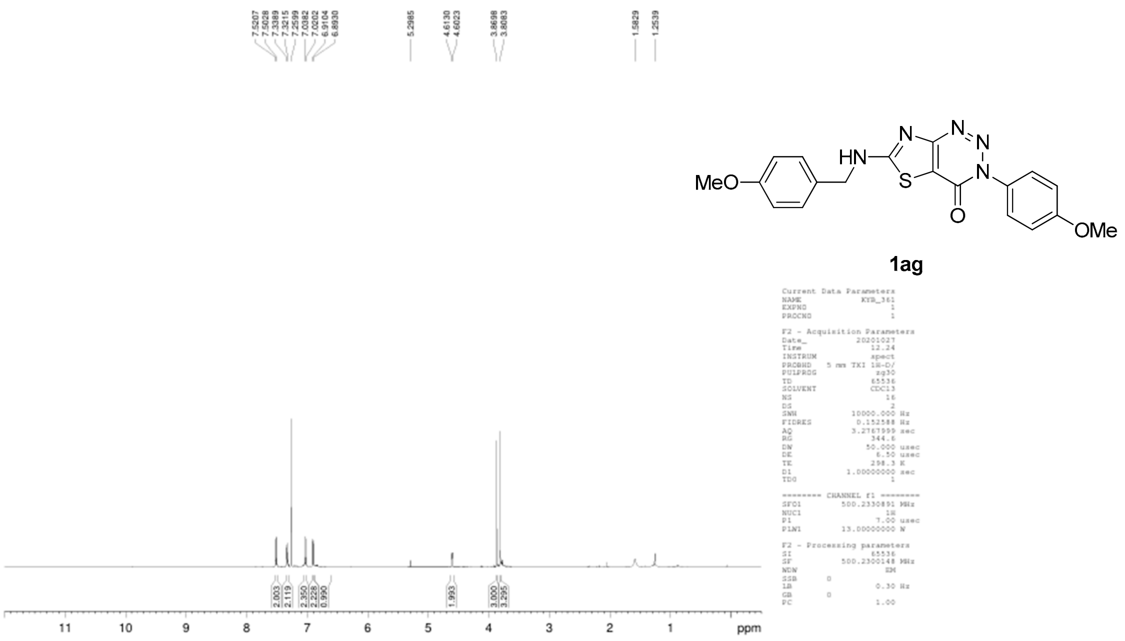

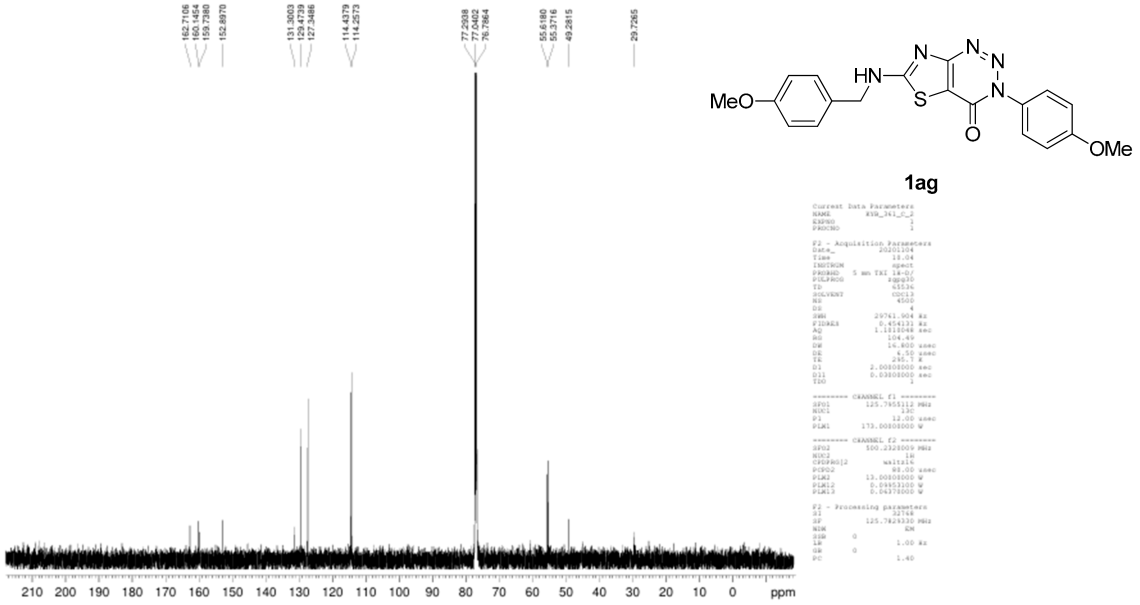

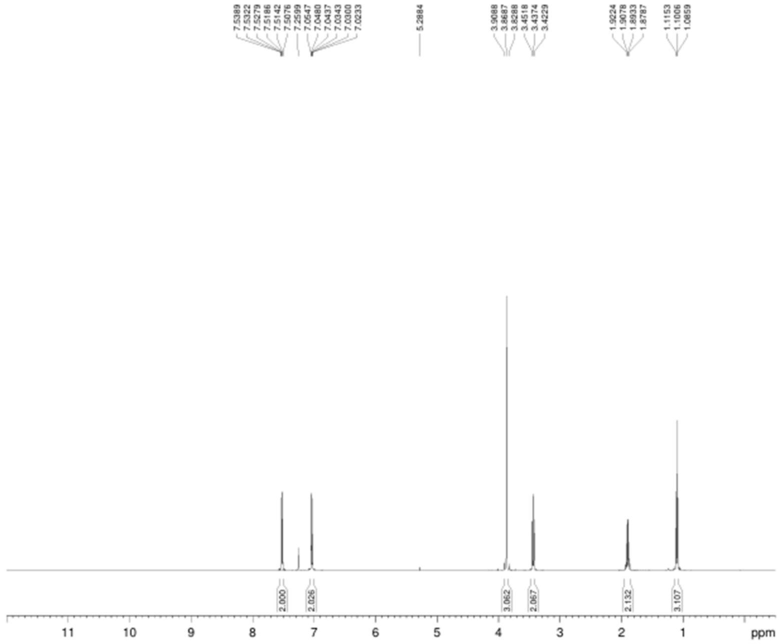

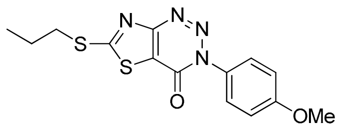

1ah

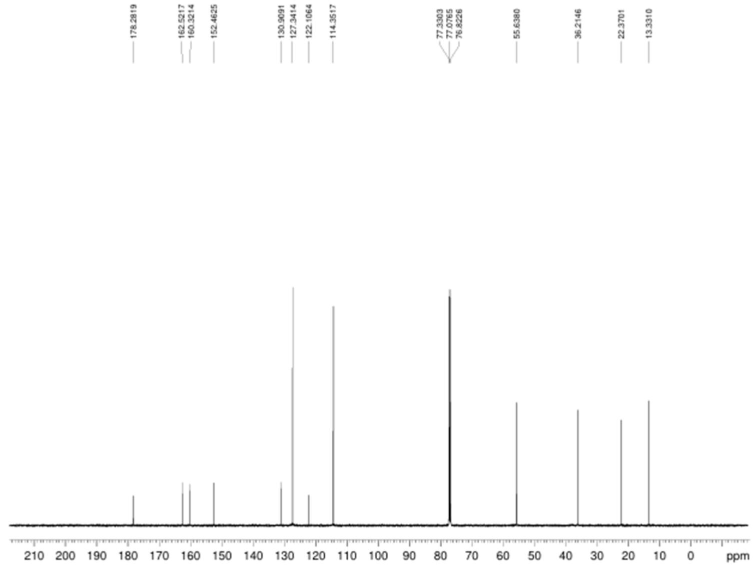

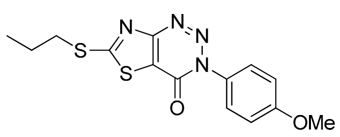

1ah

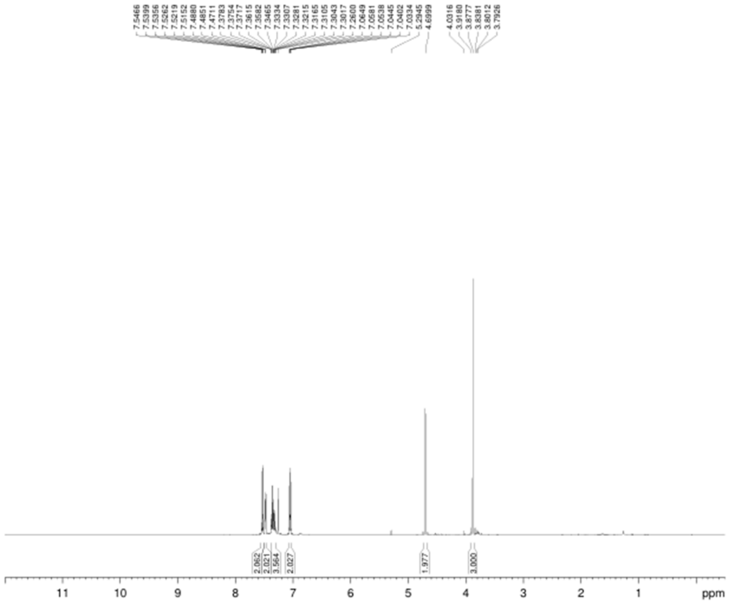

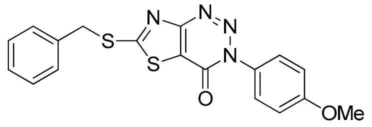

1ai

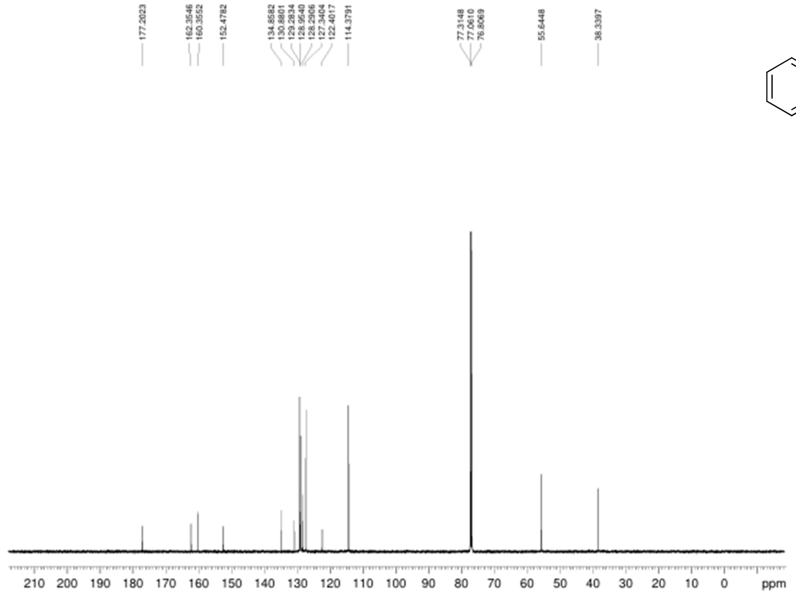

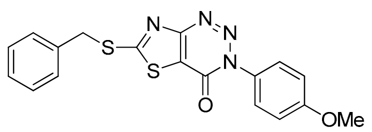

1ai

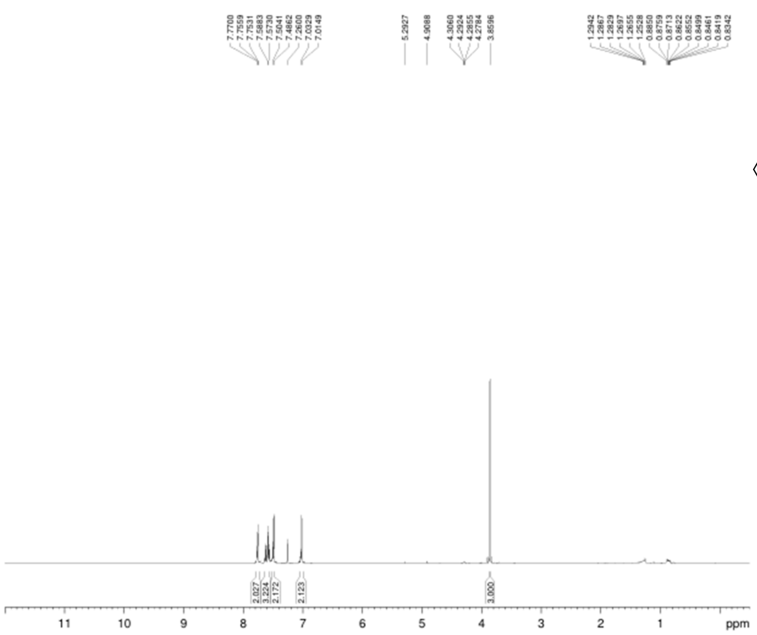
7.7700
7.7559
7.7531
7.5883
7.5730
7.5041
7.4862
7.2600
7.0329
7.0149
5.2927
4.9088
4.3060
4.2924
4.2855
4.2784
3.8596
2.027
3.224
2.172
2.123
3.000
11 10 9 8 7 6 5 4 3 2 1 ppm

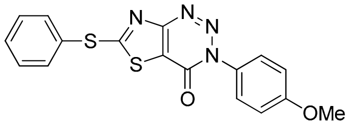
N
N
N
S
S
N
O
OMe

1aj

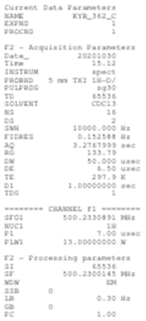

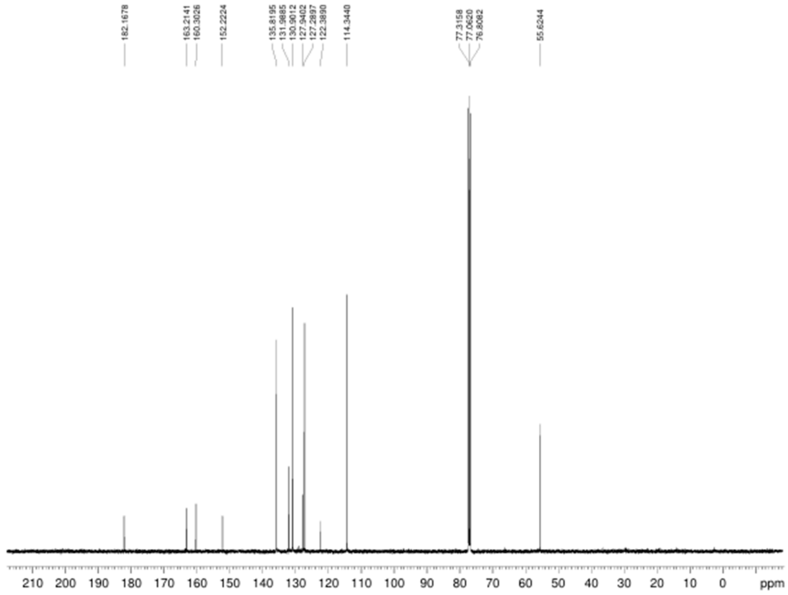
182.1678
163.2141
160.3026
152.2224
135.8195
131.9885
130.9012
127.9402
127.2897
122.3890
114.3440
77.3158
77.0620
76.8082
55.6244
210 200 190 180 170 160 150 140 130 120 110 100 90 80 70 60 50 40 30 20 10 0 ppm

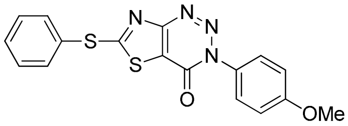
N
N
N
S
S
N
O
OMe

1aj

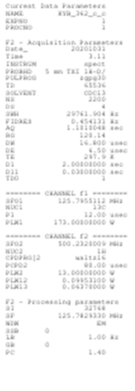

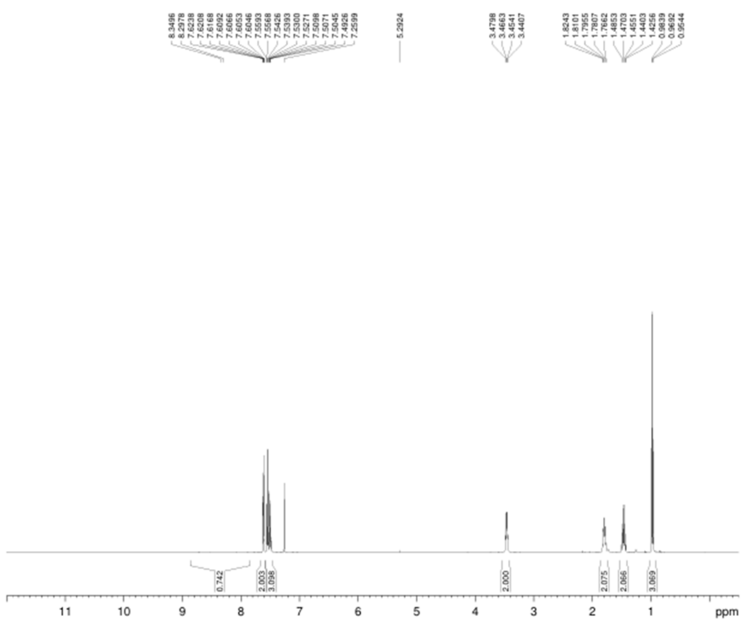

8.3496
8.2978
7.6238
7.6208
7.6168
7.6092
7.6066
7.6053
7.6046
7.5593
7.5568
7.5426
7.5393
7.5300
7.5271
7.5098
7.5071
7.5045
7.4926
7.2599
5.2924
3.4798
3.4663
3.4541
3.4407
1.8243
1.8101
1.7955
1.7807
1.7662
1.4853
1.4703
1.4551
1.4403
1.4256
0.9839
0.9692
0.9544
0.742
2.003
3.098
2.000
2.075
2.066
3.069
11
10
9
8
7
6
5
4
3
2
1
ppm

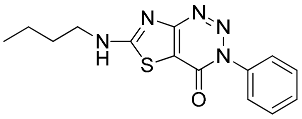

N
N
N
N
H
S
O

1ba

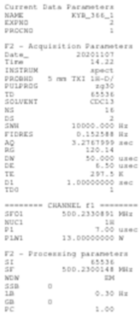

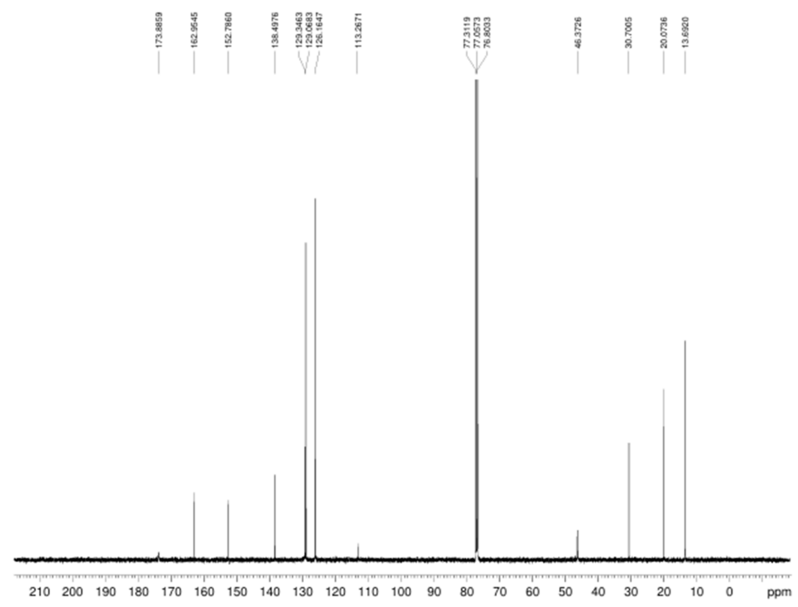

173.8859
162.9545
152.7860
138.4976
129.3463
129.0683
126.1647
113.2671
77.3119
77.0573
76.8033
46.3726
30.7005
20.0736
13.6920
210
200
190
180
170
160
150
140
130
120
110
100
90
80
70
60
50
40
30
20
10
0
ppm

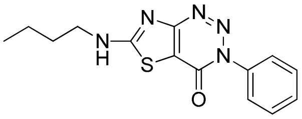

N
N
N
N
H
S
O

1ba

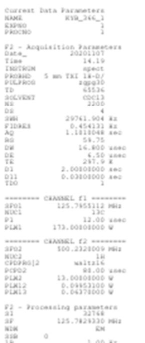

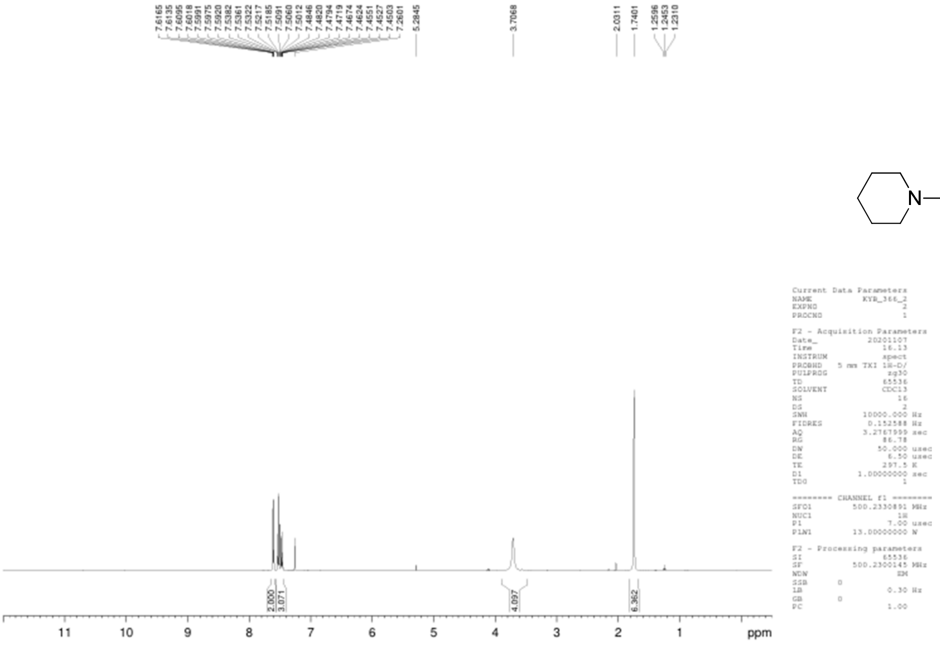
11 10 9 8 7 6 5 4 3 2 1 ppm

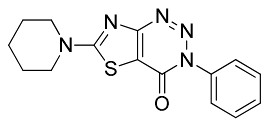

1bb

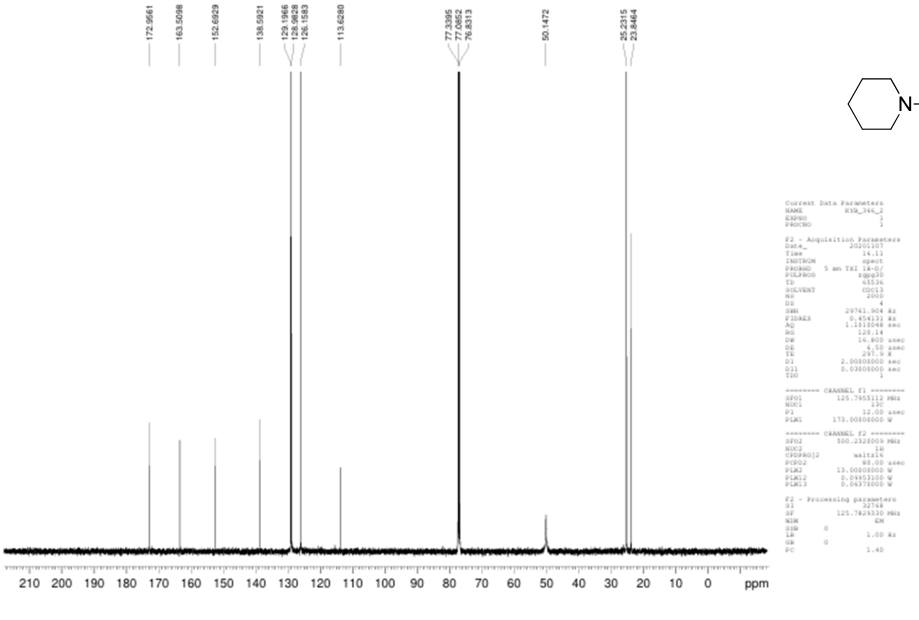
210 200 190 180 170 160 150 140 130 120 110 100 90 80 70 60 50 40 30 20 10 0 ppm

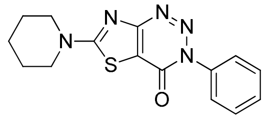

1bb

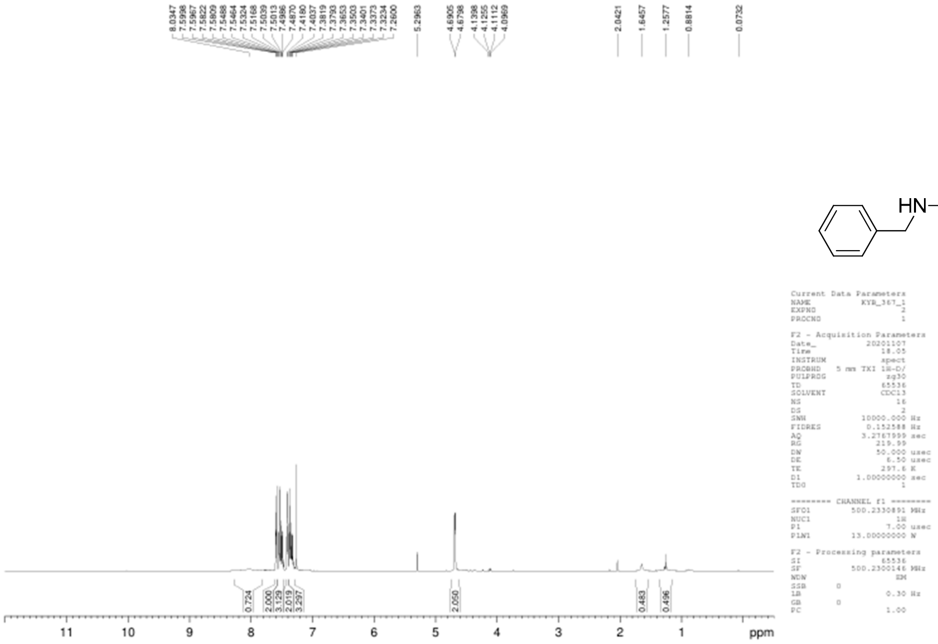

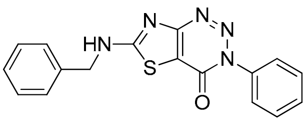

1bc

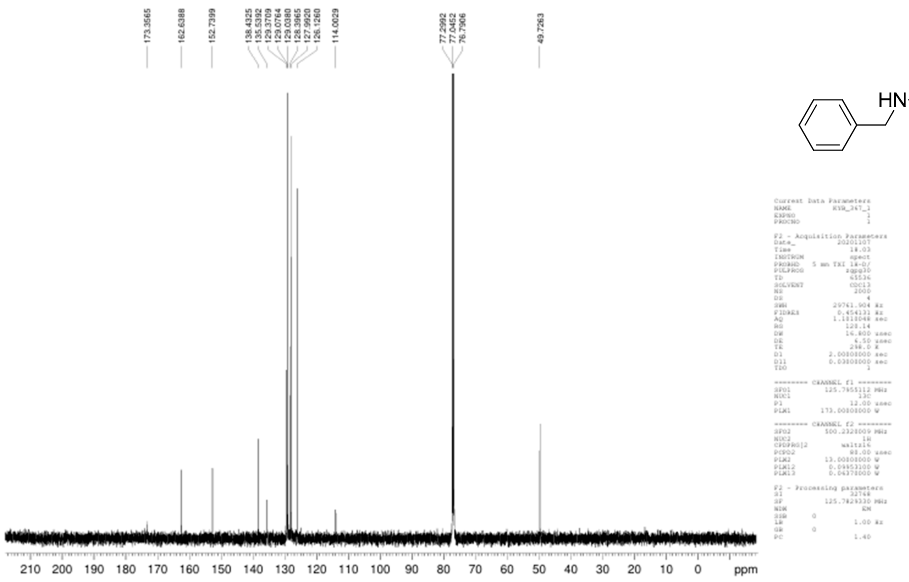

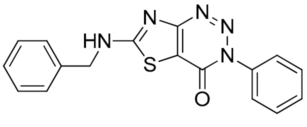

1bc

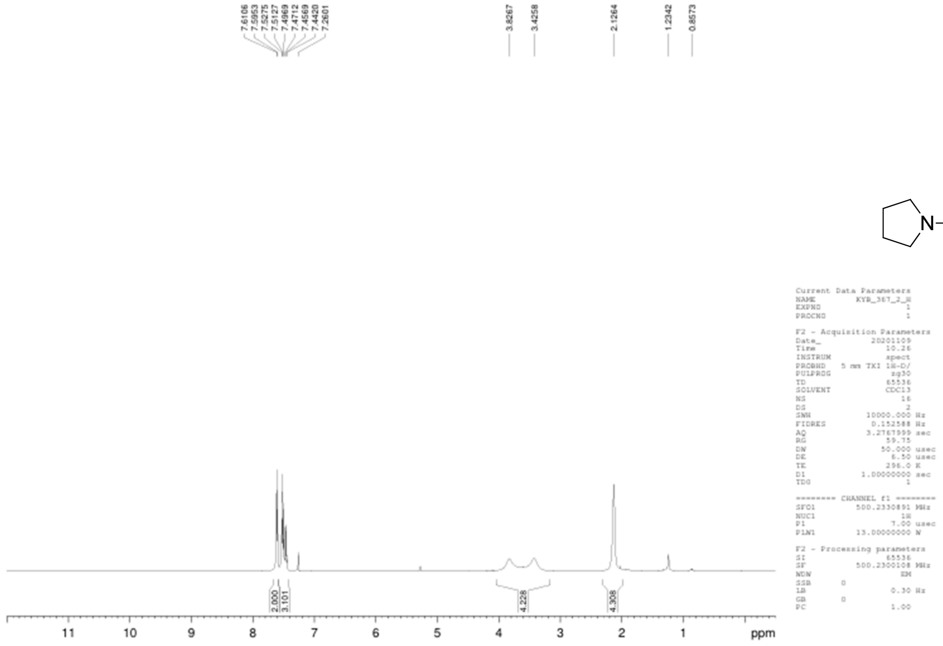
7.6106
7.5953
7.5275
7.5127
7.4969
7.4712
7.4569
7.4420
7.2601
3.8267
3.4258
2.1264
1.2342
0.8573
2.000
3.101
4.228
4.308
11 10 9 8 7 6 5 4 3 2 1 ppm
Current Data Parameters
NAME KYB_367_2_H
EXPNO 1
PROCNO 1
F2 - Acquisition Parameters
Date_ 20201109
Time 10.26
INSTRUM spect
PROBHD 5 mm TXI 1H-D/
PULPROG zg30
TD 65536
SOLVENT CDCl3
NS 16
DS 2
SWH 10000.000 Hz
FIDRES 0.152588 Hz
AQ 3.2767999 sec
RG 59.75
DW 50.000 usec
DE 6.50 usec
TE 296.0 K
D1 1.00000000 sec
TD0 1
======== CHANNEL f1 ========
SFO1 500.2330891 MHz
NUC1 1H
P1 7.00 usec
PLW1 13.00000000 W
F2 - Processing parameters
SI 65536
SF 500.2300108 MHz
WDW EM
SSB 0
LB 0.30 Hz
GB 0
PC 1.00

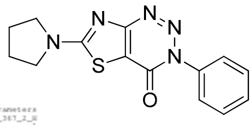
N
N
N
N
S
O

1bd

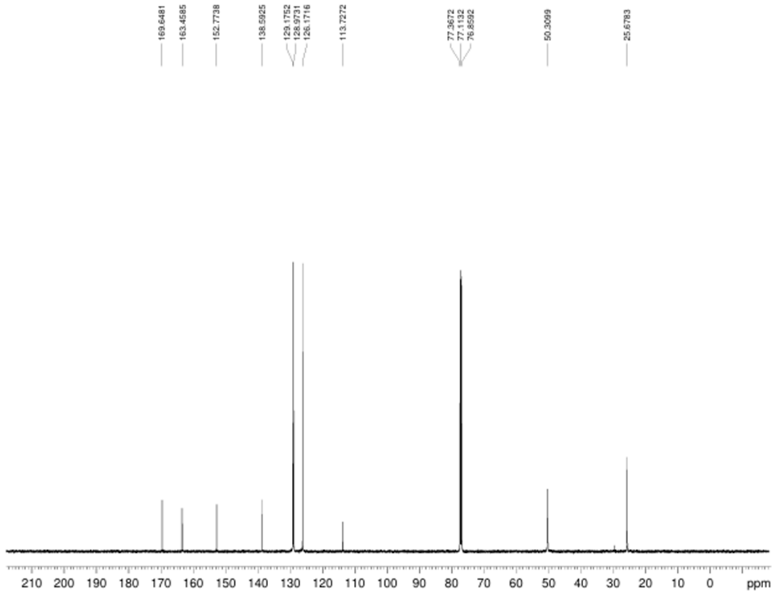
169.6481
163.4585
152.7738
138.5925
129.1752
128.9731
126.1716
113.7272
77.3672
77.1132
76.8592
50.3099
25.6783
210 200 190 180 170 160 150 140 130 120 110 100 90 80 70 60 50 40 30 20 10 0 ppm

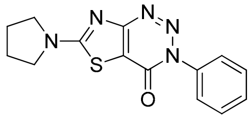
N
N
N
N
S
O

1bd

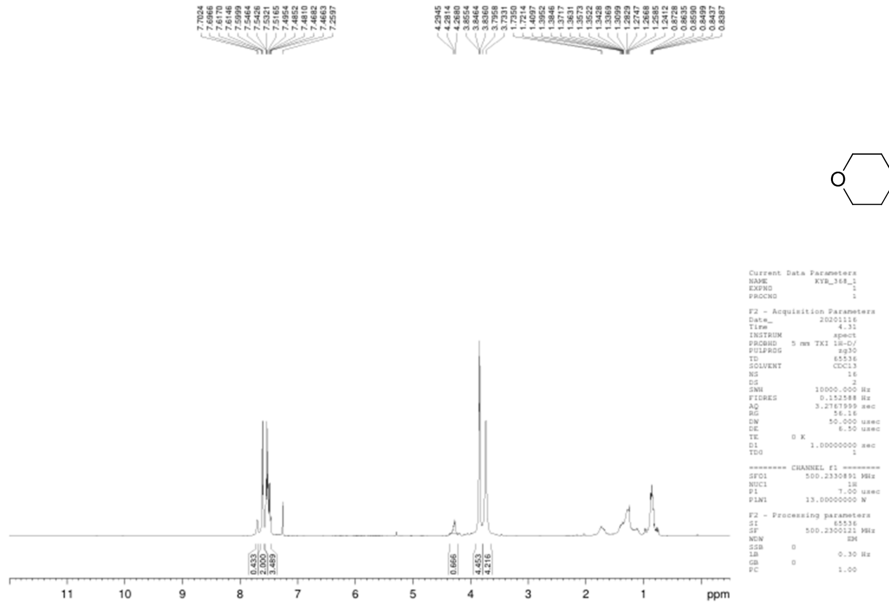

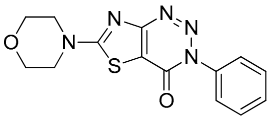

1be

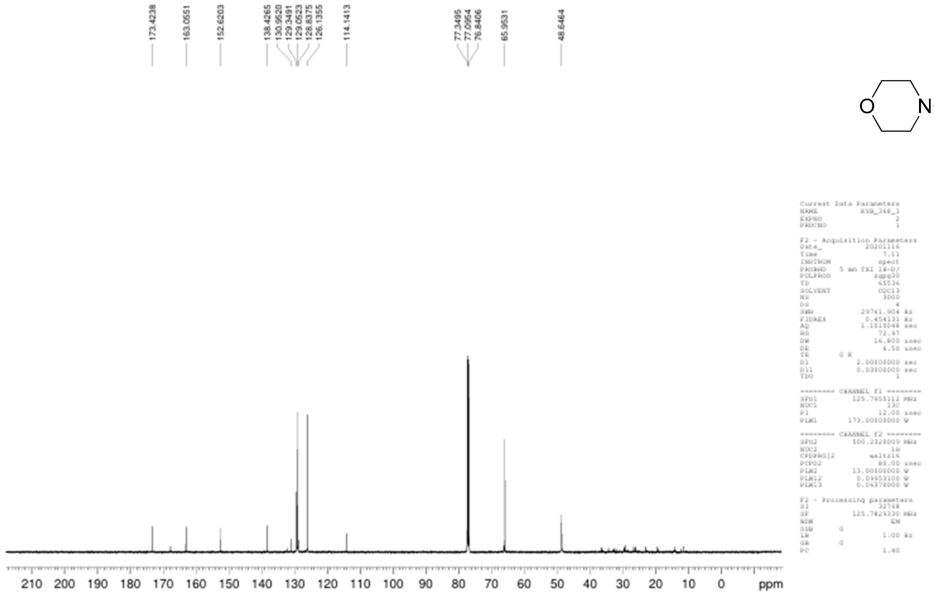
173.4238
163.0551
152.6203
138.4265
130.9520
129.3491
129.0523
128.8375
126.1355
114.1413
77.3495
77.0954
76.8406
65.9531
48.6464
ppm

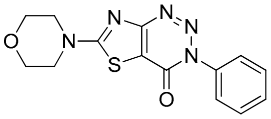

1be

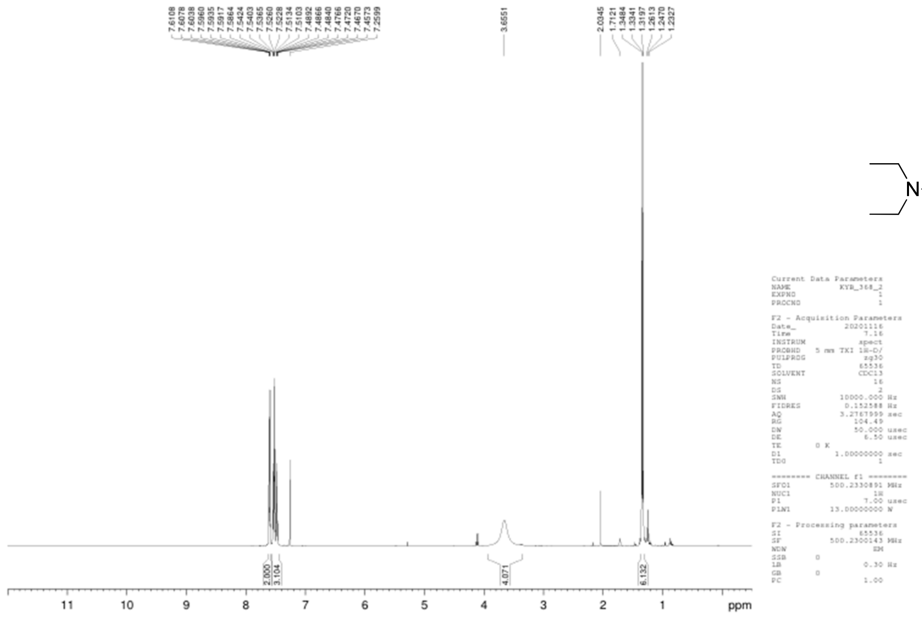

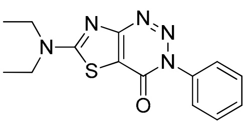

1bf

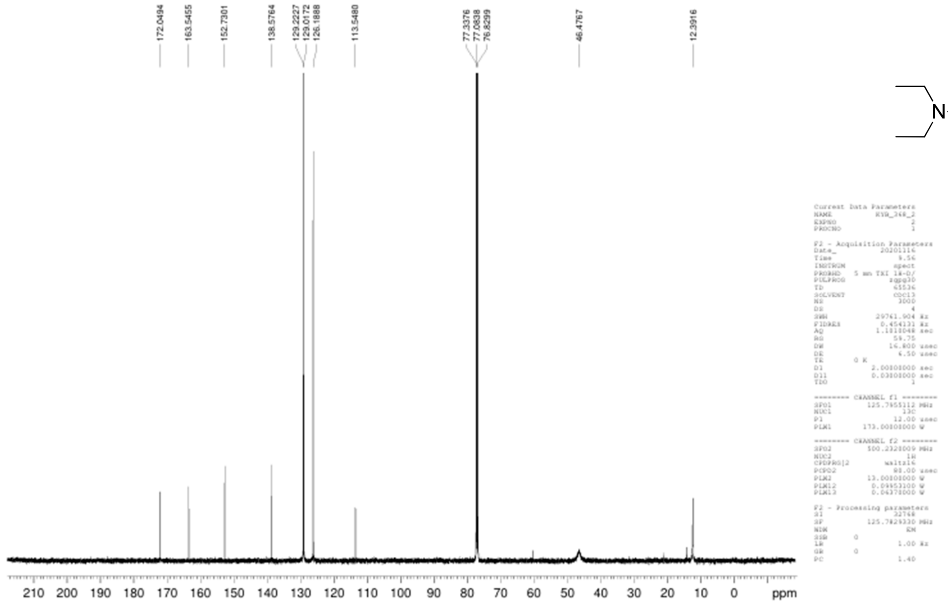

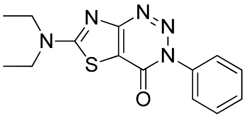

1bf

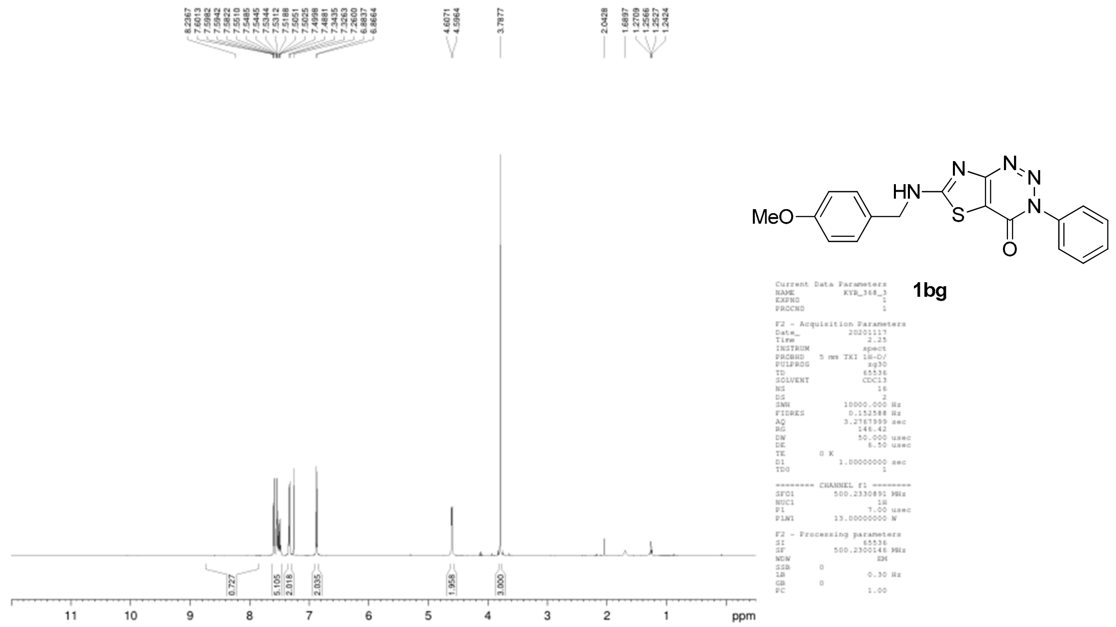

1bg

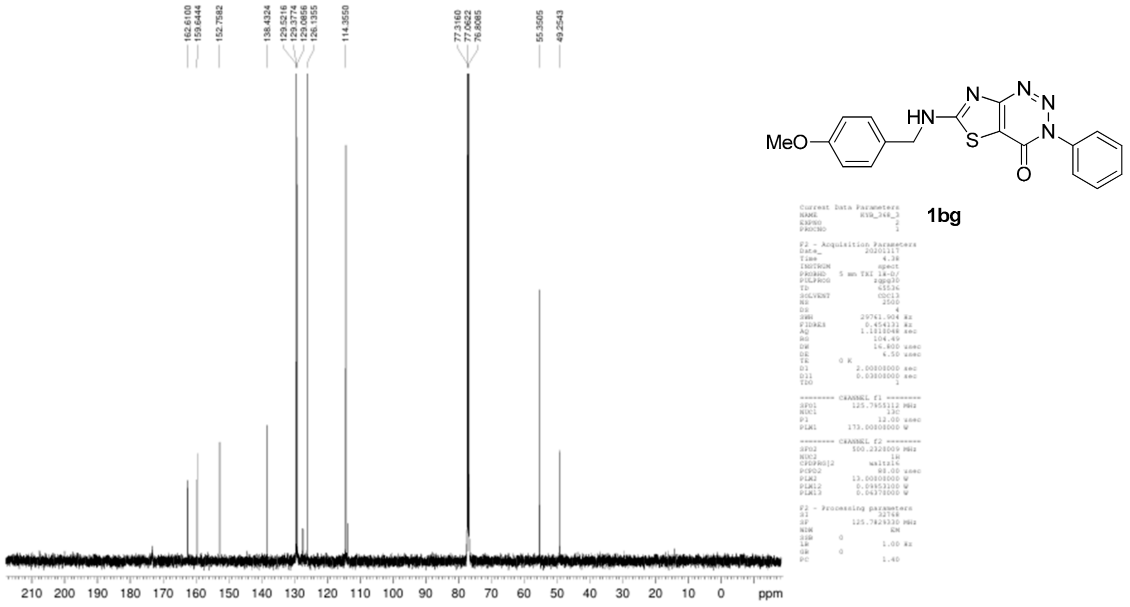

1bg

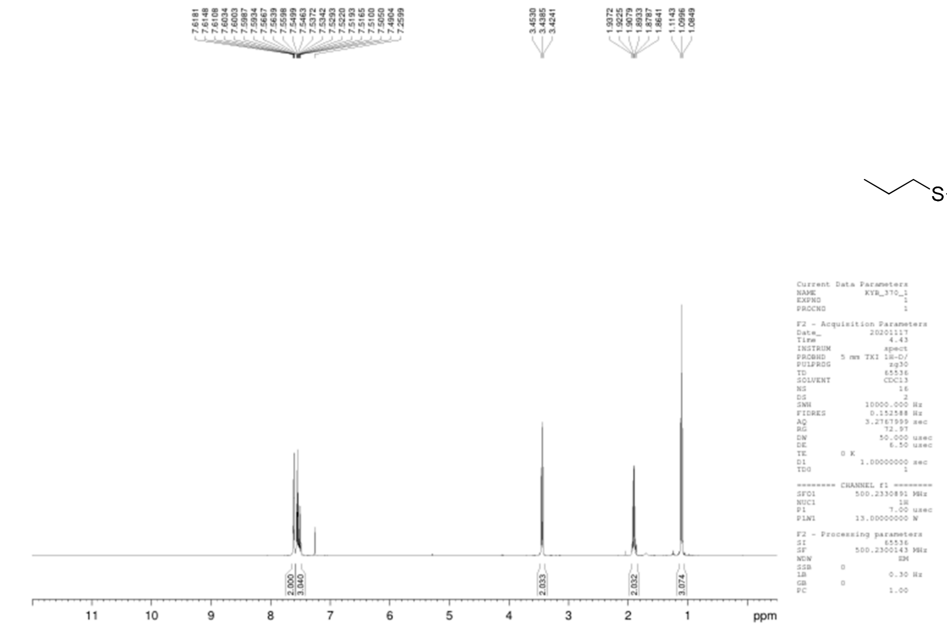

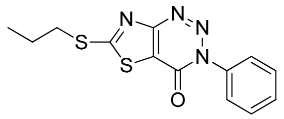

1bh

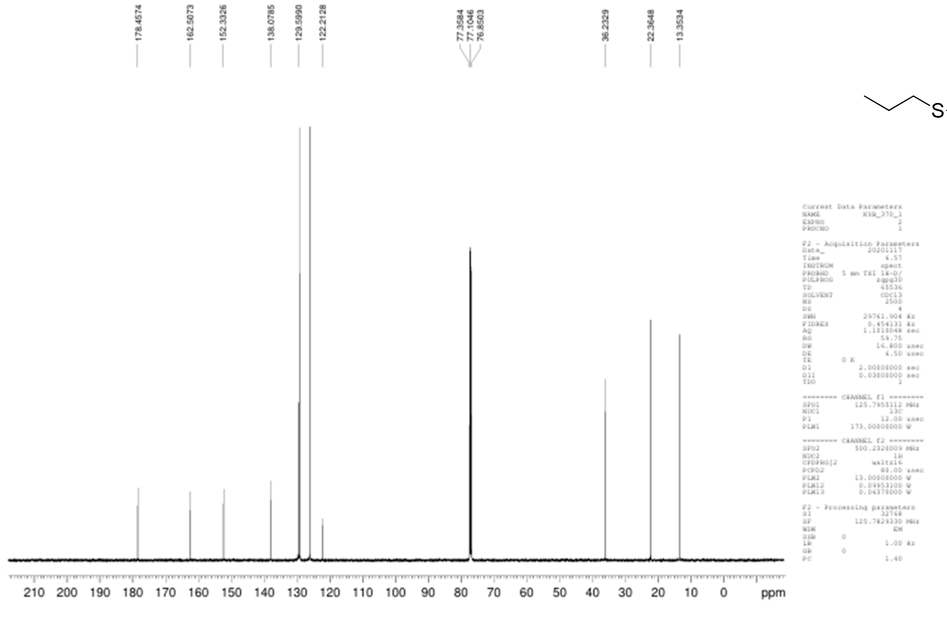

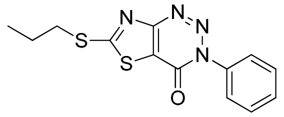

1bh

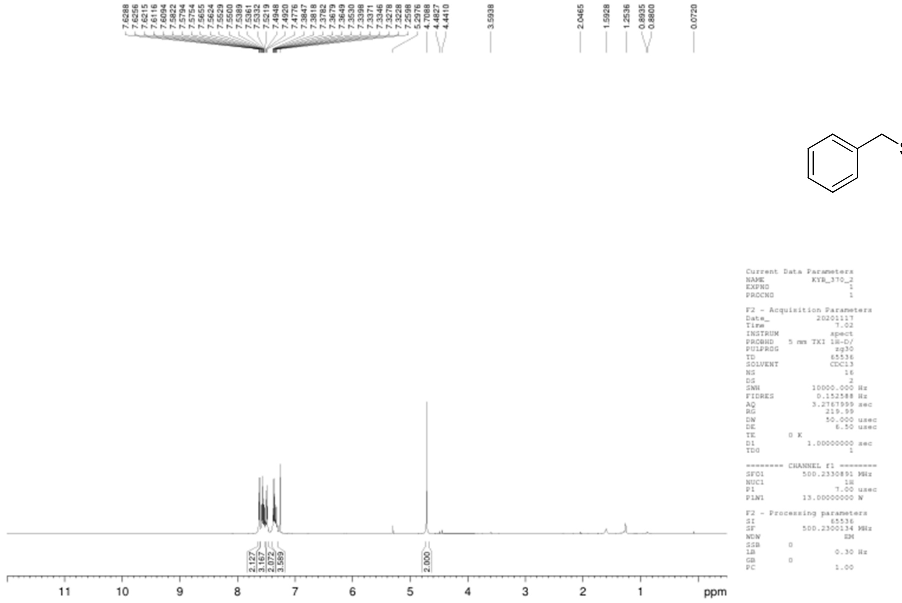
7.6288
7.6256
7.6215
7.6116
7.6094
7.5822
7.5794
7.5754
7.5655
7.5624
7.5529
7.5500
7.5389
7.5361
7.5332
7.5219
7.4948
7.4920
7.4776
7.3847
7.3818
7.3782
7.3679
7.3649
7.3530
7.3398
7.3371
7.3346
7.3278
7.3228
7.2598
5.2976
4.7088
4.4827
4.4410
3.5938
2.0465
1.5928
1.2536
0.8935
0.8800
0.0720
2.127
3.167
2.072
3.589
2.000
11
10
9
8
7
6
5
4
3
2
1
ppm

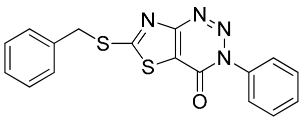
N
N
N
N
S
S
O

1bi

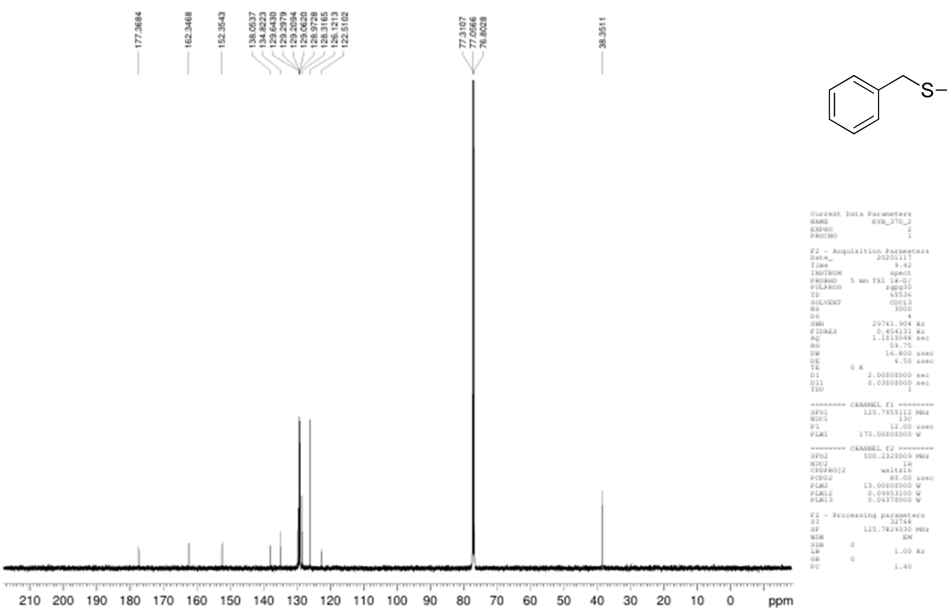
177.3684
162.3468
152.3543
138.0537
134.8223
129.6430
129.2979
129.2094
129.0620
128.9728
128.3165
126.1213
122.5102
77.3107
77.0566
76.8028
38.3511
210
200
190
180
170
160
150
140
130
120
110
100
90
80
70
60
50
40
30
20
10
0
ppm

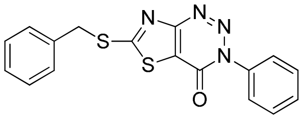
N
N
N
N
S
S
O

1bi

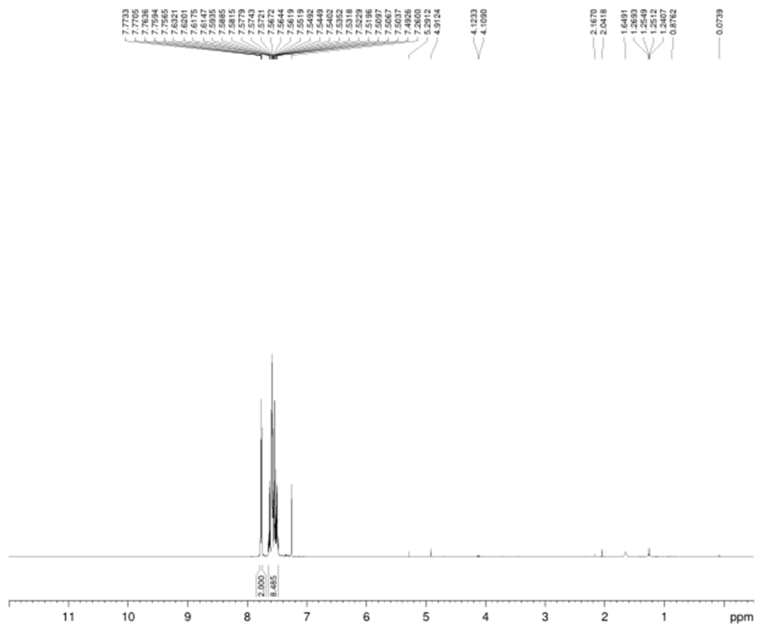
7.7733
7.7705
7.7636
7.7594
7.7565
7.6321
7.6201
7.6175
7.6147
7.5935
7.5885
7.5815
7.5779
7.5743
7.5721
7.5672
7.5644
7.5619
7.5519
7.5492
7.5449
7.5402
7.5352
7.5318
7.5229
7.5196
7.5097
7.5067
7.5037
7.4926
7.2600
5.2912
4.9124
4.1233
4.1090
2.1670
2.0418
1.6491
1.2693
1.2549
1.2512
1.2407
0.8762
0.0739
2.000
8.485
11
10
9
8
7
6
5
4
3
2
1
ppm
Current Data Parameters
NAME KYR_370_3
EXPNO 1
PROCNO 1
F2 - Acquisition Parameters
Date_ 20201117
Time 23.05
INSTRUM spect
PROBHD 5 mm TXI 1H-D/
PULPROG zg30
TD 65536
SOLVENT CDCl3
NS 16
DS 2
SWH 10000.000 Hz
FIDRES 0.152588 Hz
AQ 3.2767999 sec
RG 120.14
DW 50.000 usec
DE 6.50 usec
TE 0 K
D1 1.00000000 sec
TD0 1
======== CHANNEL f1 ========
SFO1 500.2330891 MHz
NUC1 1H
P1 7.00 usec
PLW1 13.00000000 W
F2 - Processing parameters
SI 65536
SF 500.2300146 MHz
WDW EM
SSB 0
LB 0.30 Hz
GB 0
PC 1.00

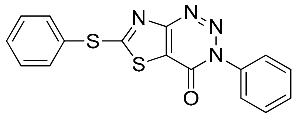
N
N
N
S
S
N
O

1bj

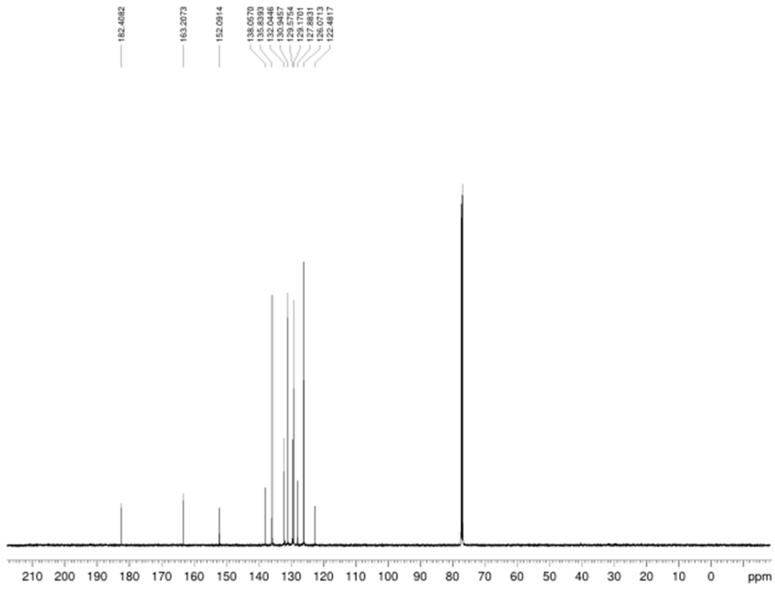
182.4082
163.2073
152.0914
138.0570
135.8393
132.0446
130.9457
129.5754
129.1701
127.8831
126.0713
122.4817
210
200
190
180
170
160
150
140
130
120
110
100
90
80
70
60
50
40
30
20
10
0
ppm
Current Data Parameters
NAME KYR_370_3
EXPNO 2
PROCNO 1
F2 - Acquisition Parameters
Date_ 20201118
Time 2.11
INSTRUM spect
PROBHD 5 mm TXI 1H-D/
PULPROG zgpg30
TD 65536
SOLVENT CDCl3
NS 3500
DS 4
SWH 29761.904 Hz
FIDRES 0.454131 Hz
AQ 1.1010048 sec
RG 59.75
DW 16.800 usec
DE 6.50 usec
TE 0 K
D1 2.00000000 sec
D11 0.03000000 sec
TD0 1
======== CHANNEL f1 ========
SFO1 125.7955112 MHz
NUC1 13C
P1 12.00 usec
PLW1 173.00000000 W
======== CHANNEL f2 ========
SFO2 500.2320009 MHz
NUC2 1H
CPDPRG[2 waltz16
PCPD2 80.00 usec
PLW2 13.00000000 W
PLW12 0.09953100 W
PLW13 0.06378000 W
F2 - Processing parameters
SI 32768
SF 125.7829330 MHz
WDW EM
SSB 0
LB 1.00 Hz
GB 0
PC 1.40

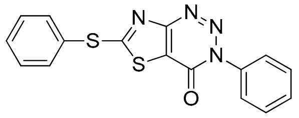
N
N
N
S
S
N
O

1bj

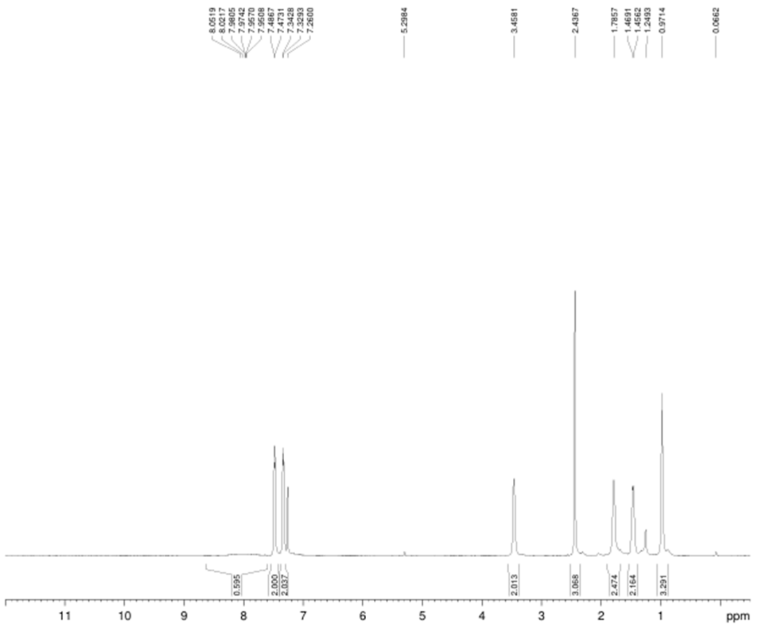

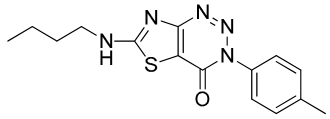

1ca

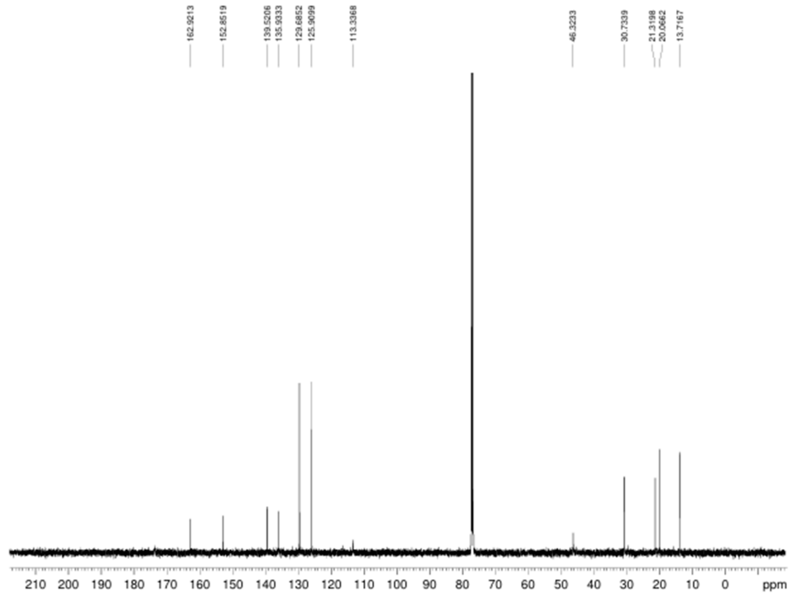

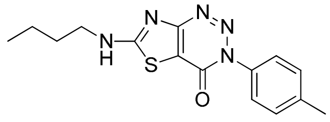

1ca

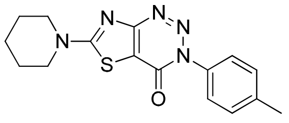

1cb

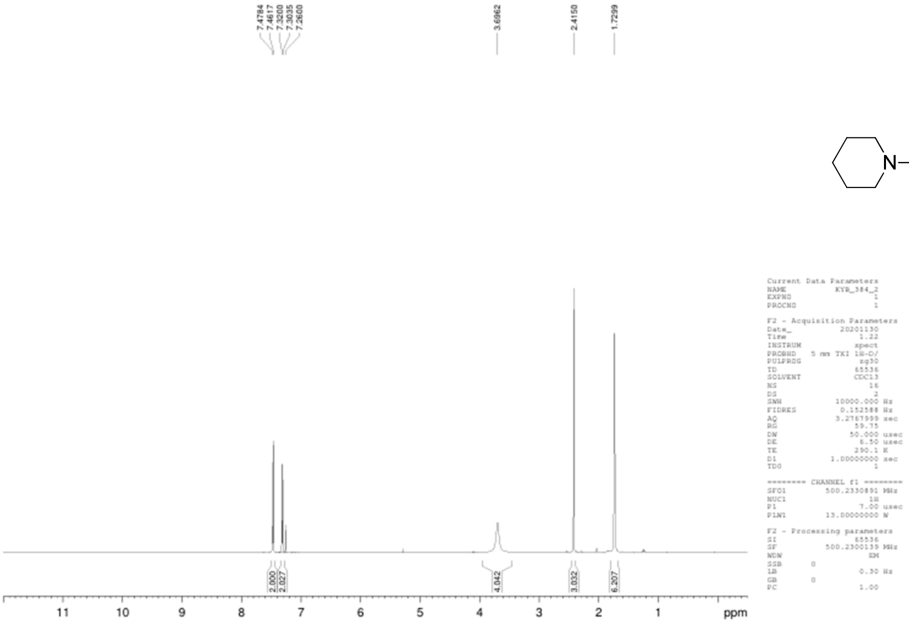
7.4784
7.4617
7.3200
7.3035
7.2600
3.6962
2.4150
1.7299
2.000
2.027
4.042
3.032
6.207
ppm

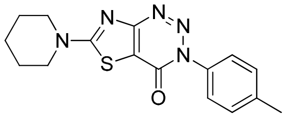

1cb

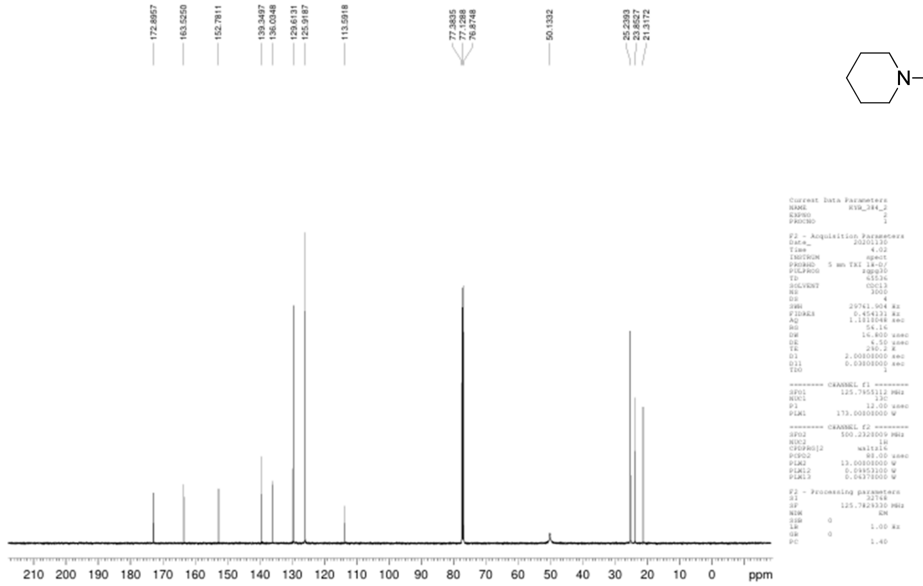
172.8957
163.5250
152.7811
139.3497
136.0348
129.6131
125.9187
113.5918
77.3835
77.1288
76.8748
50.1332
25.2393
23.8527
21.3172
ppm

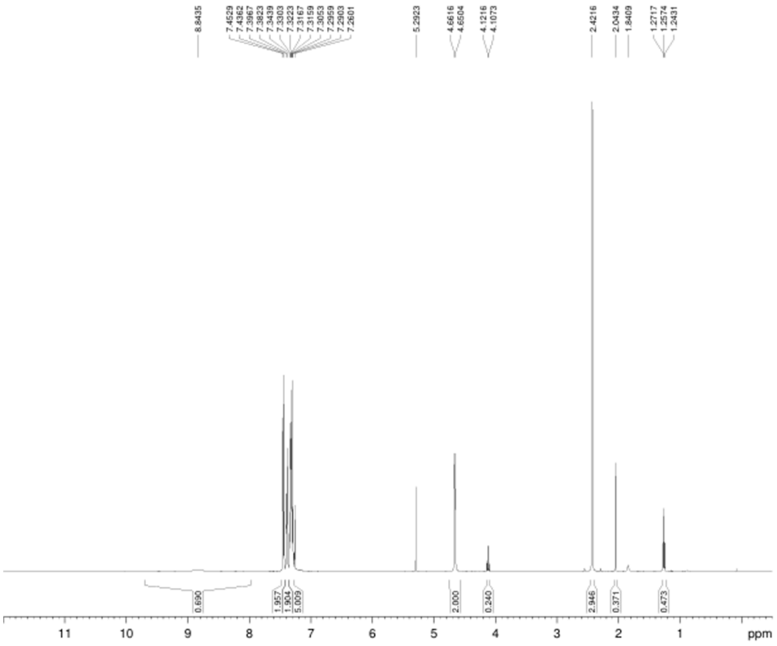
8.8435
7.4529
7.4362
7.3967
7.3823
7.3439
7.3303
7.3223
7.3167
7.3159
7.3053
7.2959
7.2903
7.2601
5.2923
4.6616
4.6504
4.1216
4.1073
2.4216
2.0434
1.8409
1.2717
1.2574
1.2431
0.690
1.957
1.904
5.009
2.000
0.240
2.946
0.371
0.473
11 10 9 8 7 6 5 4 3 2 1 ppm
Current Data Parameters
NAME KYR_384_3
EXPNO 1
PROCNO 1
F2 - Acquisition Parameters
Date_ 20201130
Time 4.07
INSTRUM spect
PROBHD 5 mm TXI 1H-D/
PULPROG zg30
TD 65536
SOLVENT CDCl3
NS 16
DS 2
SWH 10000.000 Hz
FIDRES 0.152588 Hz
AQ 3.2767999 sec
RG 86.78
DW 50.000 usec
DE 6.50 usec
TE 289.6 K
D1 1.00000000 sec
TD0 1
======== CHANNEL f1 ========
SFO1 500.2330891 MHz
NUC1 1H
P1 7.00 usec
PLW1 13.00000000 W
F2 - Processing parameters
SI 65536
SF 500.2300134 MHz
WDW EM
SSB 0
LB 0.30 Hz
GB 0
PC 1.00

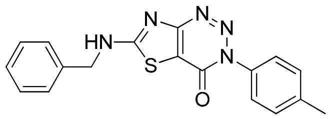
HN
N
N
N
N
S
O

1cc

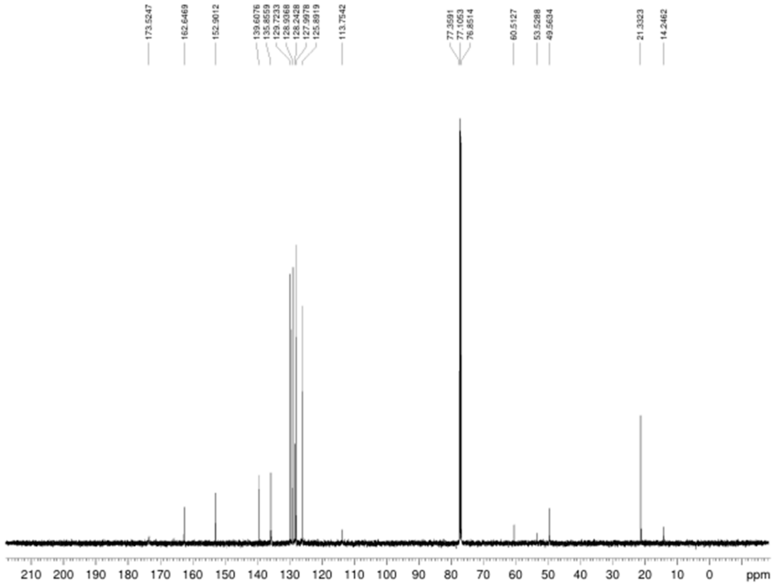
173.5247
162.6469
152.9012
139.6076
135.8559
129.7233
128.9368
128.2428
127.9978
125.8919
113.7542
77.3591
77.1053
76.8514
60.5127
53.5288
49.5634
21.3323
14.2462
210 200 190 180 170 160 150 140 130 120 110 100 90 80 70 60 50 40 30 20 10 0 ppm
Current Data Parameters
NAME KYR_384_3
EXPNO 2
PROCNO 1
F2 - Acquisition Parameters
Date_ 20201130
Time 6.46
INSTRUM spect
PROBHD 5 mm TXI 1H-D/
PULPROG zgpg30
TD 65536
SOLVENT CDCl3
NS 3000
DS 4
SWH 29761.904 Hz
FIDRES 0.454131 Hz
AQ 1.1010048 sec
RG 59.75
DW 16.800 usec
DE 6.50 usec
TE 290.0 K
D1 2.00000000 sec
D11 0.03000000 sec
TD0 1
======== CHANNEL f1 ========
SFO1 125.7955112 MHz
NUC1 13C
P1 12.00 usec
PLW1 173.00000000 W
======== CHANNEL f2 ========
SFO2 500.2320009 MHz
NUC2 1H
CPDPRG[2 waltz16
PCPD2 90.00 usec
PLW2 13.00000000 W
PLW12 0.09953100 W
PLW13 0.06378000 W
F2 - Processing parameters
SI 32768
SF 125.7829330 MHz
WDW EM
SSB 0
LB 1.00 Hz
GB 0
PC 1.40

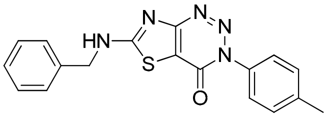
HN
N
N
N
N
S
O

1cc

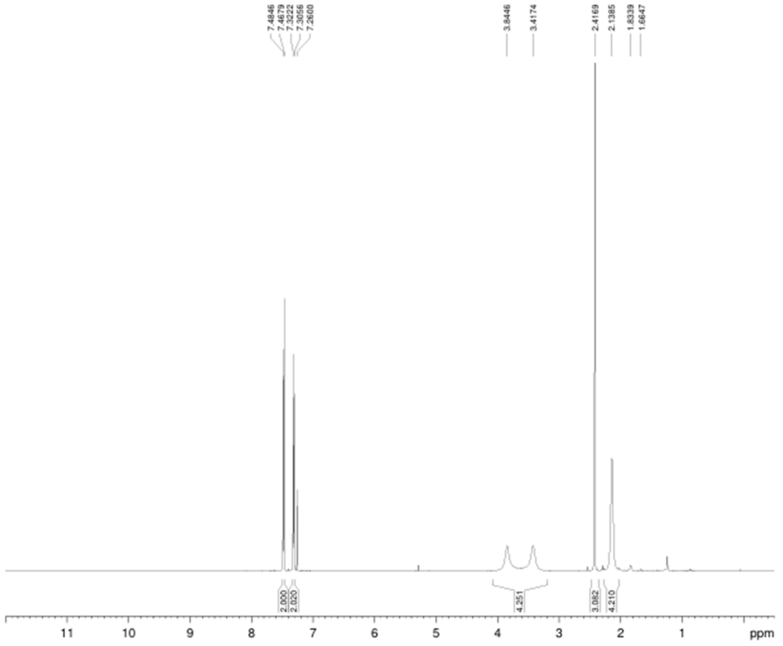

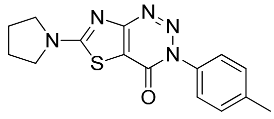

1cd

```
Current Data Parameters
NAME          KYR_385_1
EXPNO                 1
PROCNO                1

F2 - Acquisition Parameters
Date_          20201130
Time               6.51
INSTRUM           spect
PROBHD   5 mm TXI 1H-D/
PULPROG            zg30
TD                65536
SOLVENT           CDCl3
NS                   16
DS                    2
SWH          10000.000 Hz
FIDRES        0.152588 Hz
AQ           3.2767999 sec
RG                72.97
DW              50.000 usec
DE                6.50 usec
TE               289.5 K
D1          1.00000000 sec
TD0                   1

======== CHANNEL f1 ========
SFO1       500.2330891 MHz
NUC1                 1H
P1                 7.00 usec
PLW1       13.00000000 W

F2 - Processing parameters
SI                65536
SF         500.2300139 MHz
WDW                  EM
SSB      0
LB                 0.30 Hz
GB       0
PC                 1.00
```

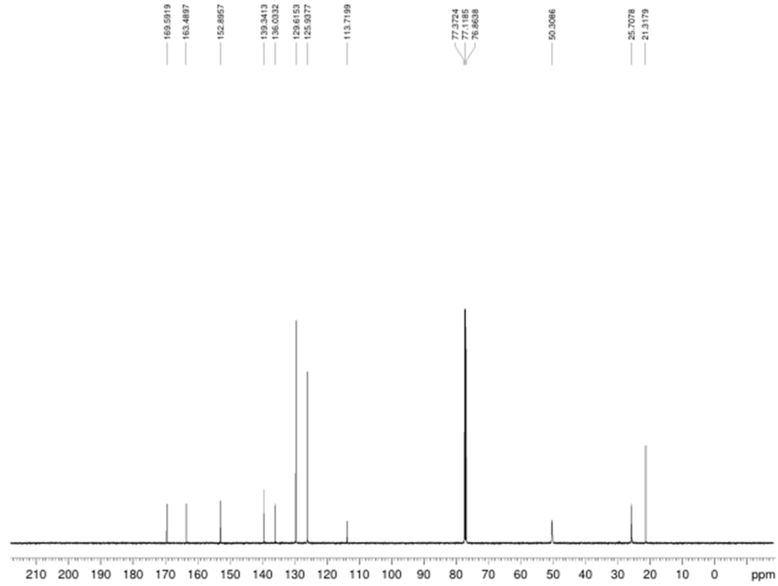

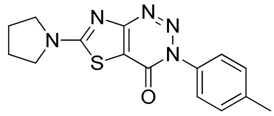

1cd

```
Current Data Parameters
NAME          KYR_385_1
EXPNO                 2
PROCNO                1

F2 - Acquisition Parameters
Date_          20201130
Time               [illegible]
INSTRUM           spect
PROBHD   5 mm TXI 1H-D/
PULPROG          zgpg30
TD                65536
SOLVENT           CDCl3
NS                 2900
DS                    4
SWH          29761.904 Hz
FIDRES        0.454131 Hz
AQ           1.1010048 sec
RG                [illegible]
DW              16.800 usec
DE                6.50 usec
TE               [illegible] K
D1          2.00000000 sec
D11         0.03000000 sec
TD0                   1

======== CHANNEL f1 ========
SFO1       125.7955112 MHz
NUC1                13C
P1                12.00 usec
PLW1      173.00000000 W

======== CHANNEL f2 ========
SFO2       500.2328009 MHz
NUC2                 1H
CPDPRG[2         waltz16
PCPD2             80.00 usec
PLW2       13.00000000 W
PLW12       0.09953100 W
PLW13       0.06378000 W

F2 - Processing parameters
SI                32768
SF         125.7829330 MHz
WDW                  EM
SSB      0
LB                 1.00 Hz
GB       0
PC                 1.40
```

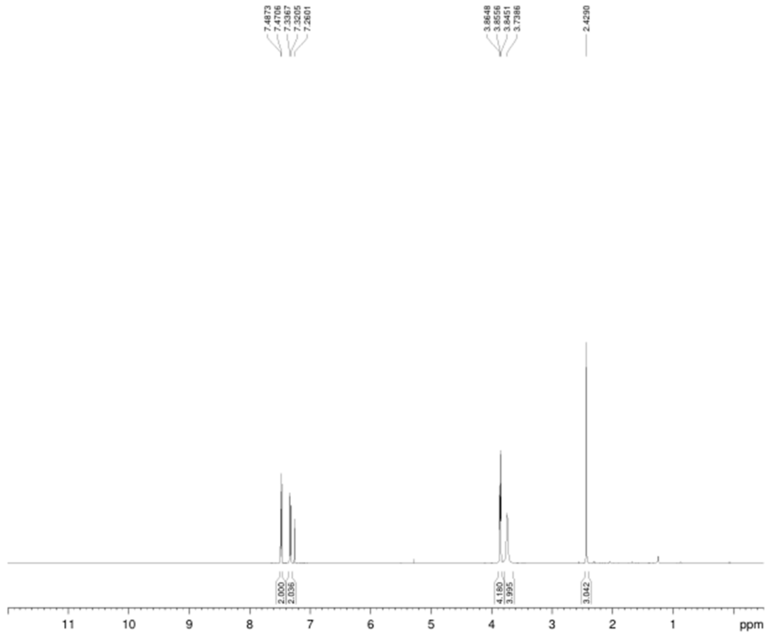
7.4873
7.4706
7.3367
7.3205
7.2601
3.8648
3.8556
3.8451
3.7386
2.4290
2.000
2.036
4.180
3.995
3.042
11 10 9 8 7 6 5 4 3 2 1 ppm
Current Data Parameters
NAME KYR_385_2
EXPNO 1
PROCNO 1
F2 - Acquisition Parameters
Date_ 20201130
Time 9.31
INSTRUM spect
PROBHD 5 mm TXI 1H-D/
PULPROG zg30
TD 65536
SOLVENT CDCl3
NS 16
DS 2
SWH 10000.000 Hz
FIDRES 0.152588 Hz
AQ 3.2767999 sec
RG 104.49
DW 50.000 usec
DE 6.50 usec
TE 290.2 K
D1 1.00000000 sec
TD0 1
======== CHANNEL f1 ========
SFO1 500.2330891 MHz
NUC1 1H
P1 7.00 usec
PLW1 13.00000000 W
F2 - Processing parameters
SI 65536
SF 500.2300140 MHz
WDW EM
SSB 0
LB 0.30 Hz
GB 0
PC 1.00

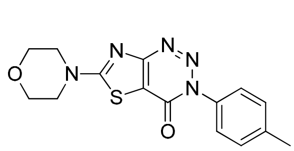

1ce

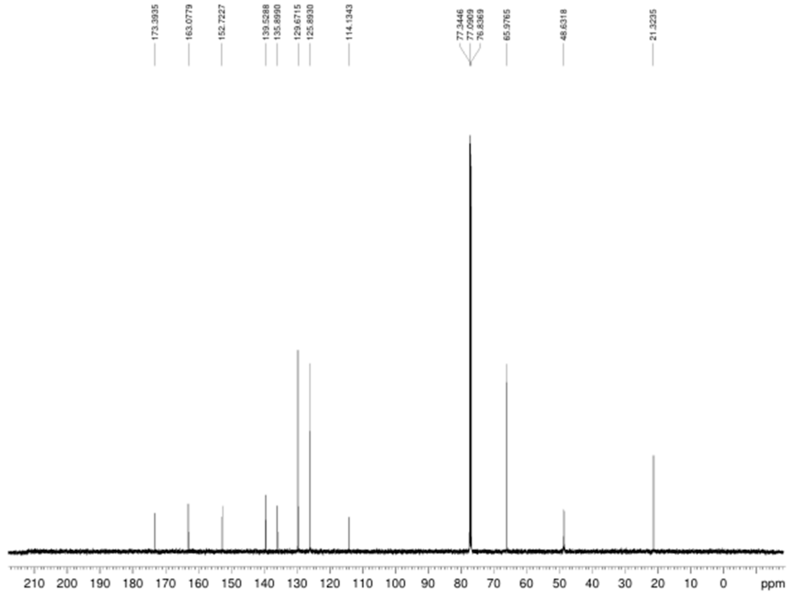
173.3935
163.0779
152.7227
139.5288
135.8990
129.6715
125.8930
114.1343
77.3446
77.0909
76.8369
65.9765
48.6318
21.3235
210 200 190 180 170 160 150 140 130 120 110 100 90 80 70 60 50 40 30 20 10 0 ppm
Current Data Parameters
NAME KYR_385_2
EXPNO 2
PROCNO 1
F2 - Acquisition Parameters
Date_ 20201130
Time 10.26
INSTRUM spect
PROBHD 5 mm TXI 1H-D/
PULPROG zgpg30
TD 65536
SOLVENT CDCl3
NS 1024
DS 4
SWH 29761.904 Hz
FIDRES 0.454131 Hz
AQ 1.1010048 sec
RG 59.75
DW 16.800 usec
DE 6.50 usec
TE 291.9 K
D1 2.00000000 sec
D11 0.03000000 sec
TD0 1
======== CHANNEL f1 ========
SFO1 125.7955112 MHz
NUC1 13C
P1 12.00 usec
PLW1 173.00000000 W
======== CHANNEL f2 ========
SFO2 500.2320009 MHz
NUC2 1H
CPDPRG[2 waltz16
PCPD2 80.00 usec
PLW2 13.00000000 W
PLW12 0.09953100 W
PLW13 0.06378000 W
F2 - Processing parameters
SI 32768
SF 125.7829330 MHz
WDW EM
SSB 0
LB 1.00 Hz
GB 0
PC 1.40

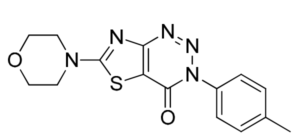

1ce

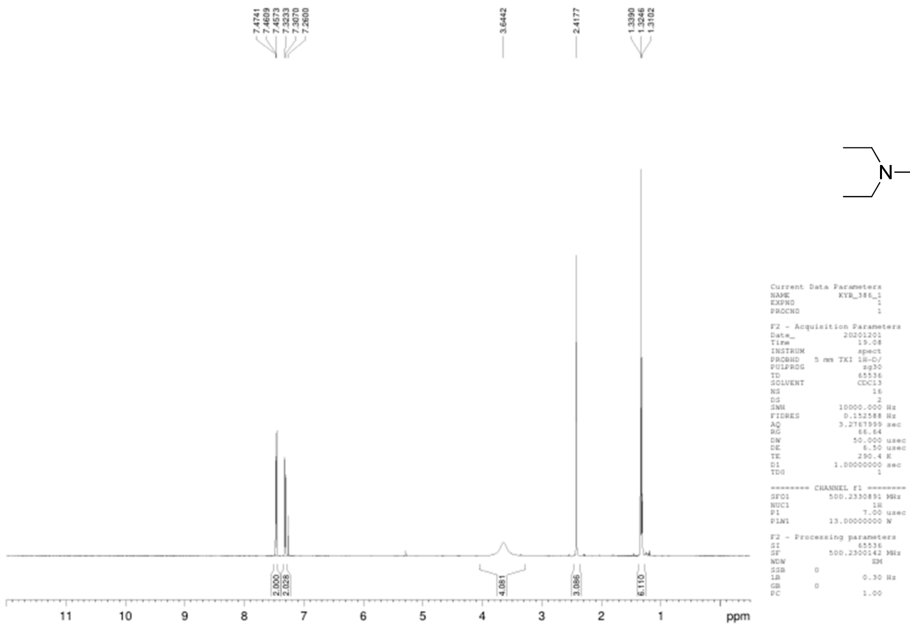
7.4741
7.4609
7.4573
7.3233
7.3070
7.2600
3.6442
2.4177
1.3390
1.3246
1.3102
2.000
2.028
4.081
3.086
6.110
ppm

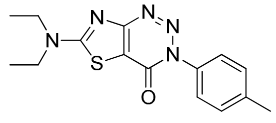

1cf

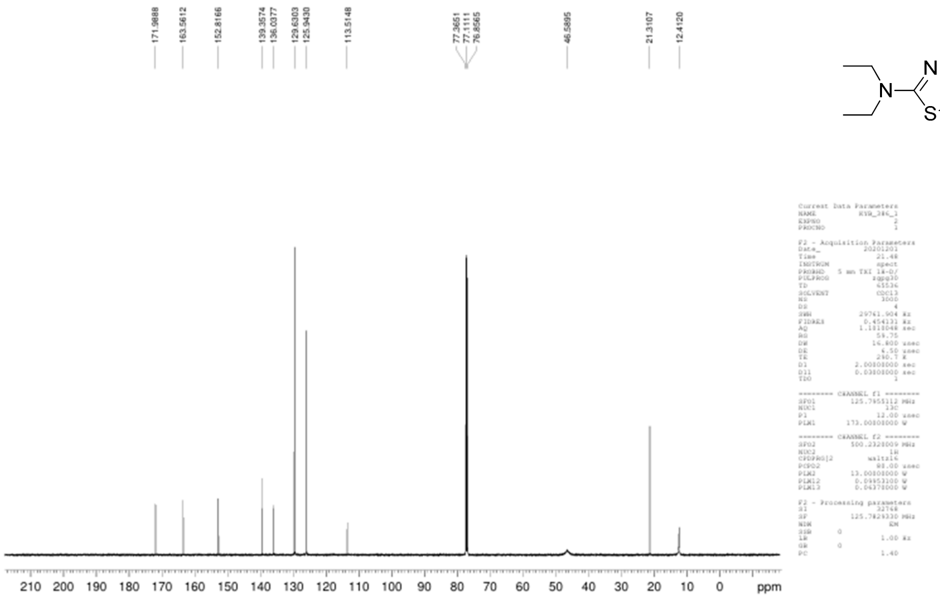
171.9888
163.5612
152.8166
139.3574
136.0377
129.6303
125.9430
113.5148
77.3651
77.1111
76.8565
46.5895
21.3107
12.4120
ppm

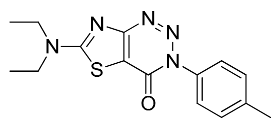

1cf

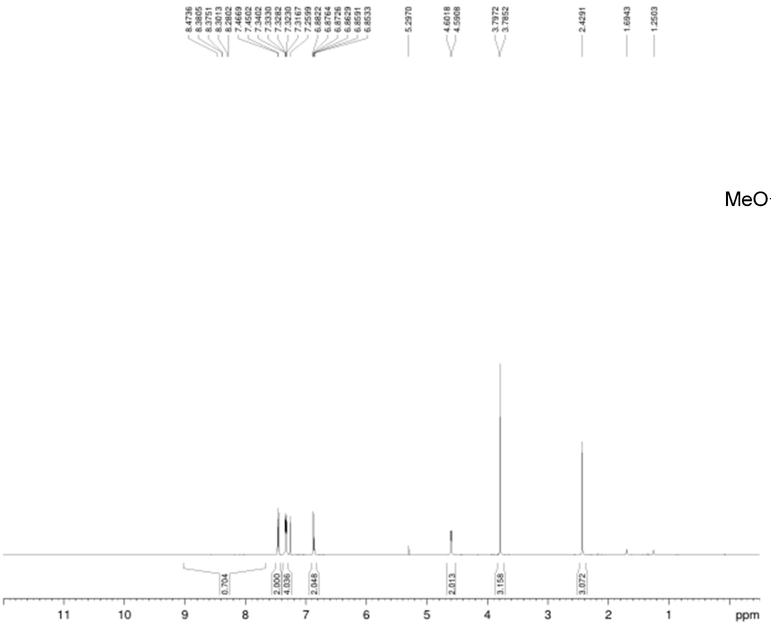
8.4736
8.3865
8.3751
8.3013
8.2802
7.4669
7.4502
7.3402
7.3330
7.3282
7.3230
7.3167
7.2599
6.8822
6.8764
6.8726
6.8629
6.8591
6.8533
5.2970
4.6018
4.5908
3.7972
3.7852
2.4291
1.6943
1.2503
0.704
2.000
4.036
2.048
2.013
3.158
3.072
11 10 9 8 7 6 5 4 3 2 1 ppm

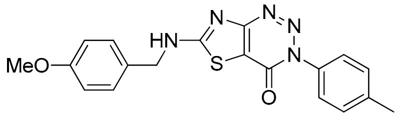
MeO
HN
N
S
N
N
N
O

1cg

Current Data Parameters
NAME KYR_386_2
EXPNO 1
PROCNO 1
F2 - Acquisition Parameters
Date_ 20201201
Time 21.53
INSTRUM spect
PROBHD 5 mm TXI 1H-D/
PULPROG zg30
TD 65536
SOLVENT CDCl3
NS 16
DS 2
SWH 10000.000 Hz
FIDRES 0.152588 Hz
AQ 3.2767999 sec
RG 146.42
DW 50.000 usec
DE 6.50 usec
TE 290.1 K
D1 1.00000000 sec
TD0 1
======== CHANNEL f1 ========
SFO1 500.2330891 MHz
NUC1 1H
P1 7.00 usec
PLW1 13.00000000 W
F2 - Processing parameters
SI 65536
SF 500.2300142 MHz
WDW EM
SSB 0
LB 0.30 Hz
GB 0
PC 1.00

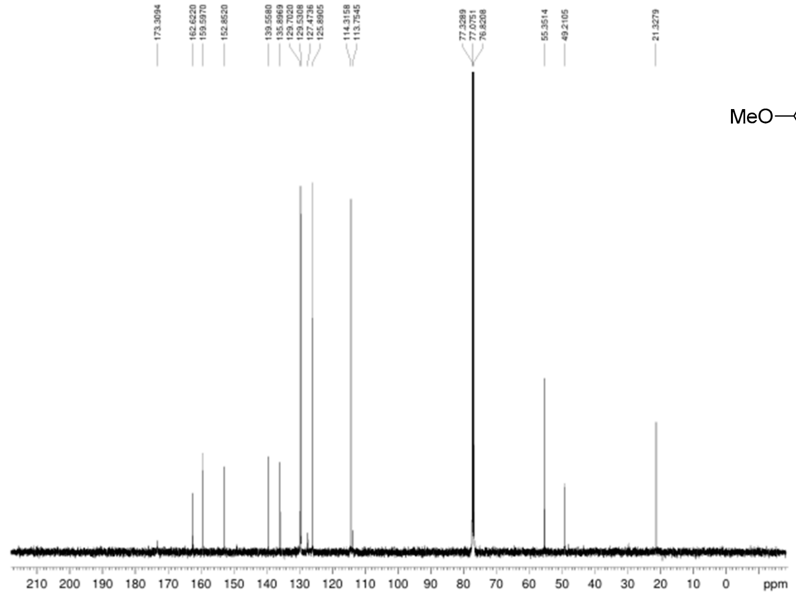
173.3094
162.6220
159.5970
152.8520
139.5580
135.8969
129.7020
129.5308
127.4736
125.8905
114.3158
113.7545
77.3289
77.0751
76.8208
55.3514
49.2105
21.3279
210 200 190 180 170 160 150 140 130 120 110 100 90 80 70 60 50 40 30 20 10 0 ppm

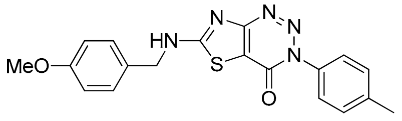
MeO
HN
N
S
N
N
N
O

1cg

Current Data Parameters
EXPNO 2
PROCNO 1
F2 - Acquisition Parameters
Date_ 20201202
INSTRUM spect
TD 65536
SOLVENT CDCl3
NS 3000
DS 4
SWH 29761.904 Hz
RG 56.16
DE 6.50 usec
TE 290.1 K
D1 2.00000000 sec
TD0 1
======== CHANNEL f1 ========
SFO1 125.7955112 MHz
NUC1 13C
P1 12.00 usec
======== CHANNEL f2 ========
NUC2 1H
CPDPRG[2 waltz16
F2 - Processing parameters
SI 32768
SF 125.7829330 MHz
WDW EM
SSB 0
LB 1.00 Hz
GB 0
PC 1.40

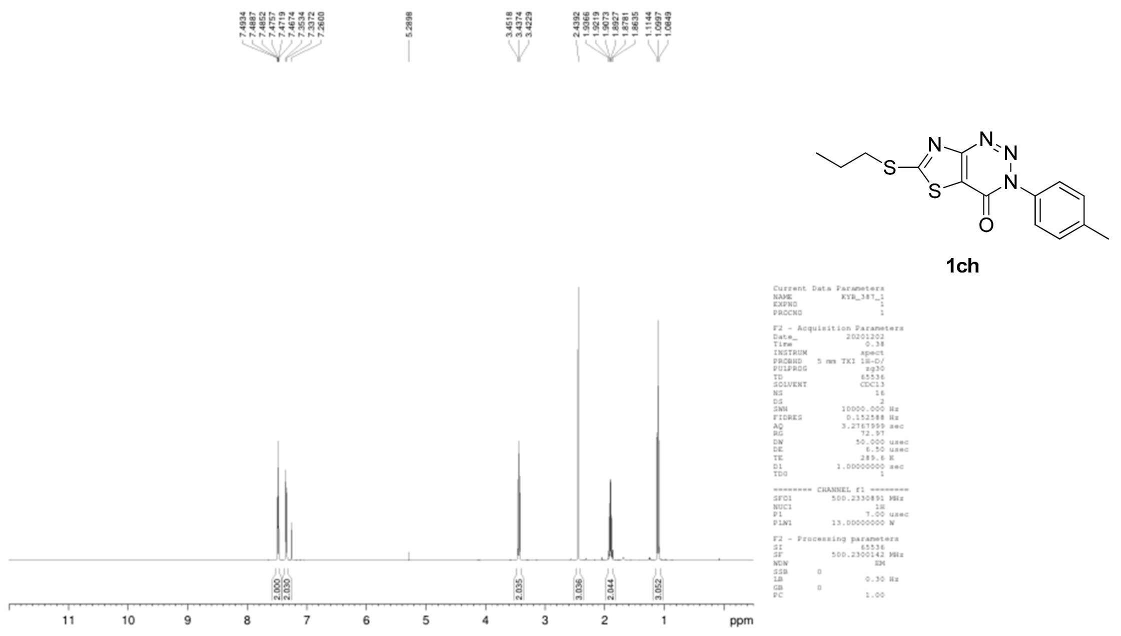
7.4934
7.4887
7.4852
7.4757
7.4719
7.4674
7.3534
7.3372
7.2600
5.2898
3.4518
3.4374
3.4229
2.4392
1.9366
1.9219
1.9073
1.8927
1.8781
1.8635
1.1144
1.0997
1.0849
1ch
2.000
2.030
2.035
3.036
2.044
3.052
11 10 9 8 7 6 5 4 3 2 1 ppm

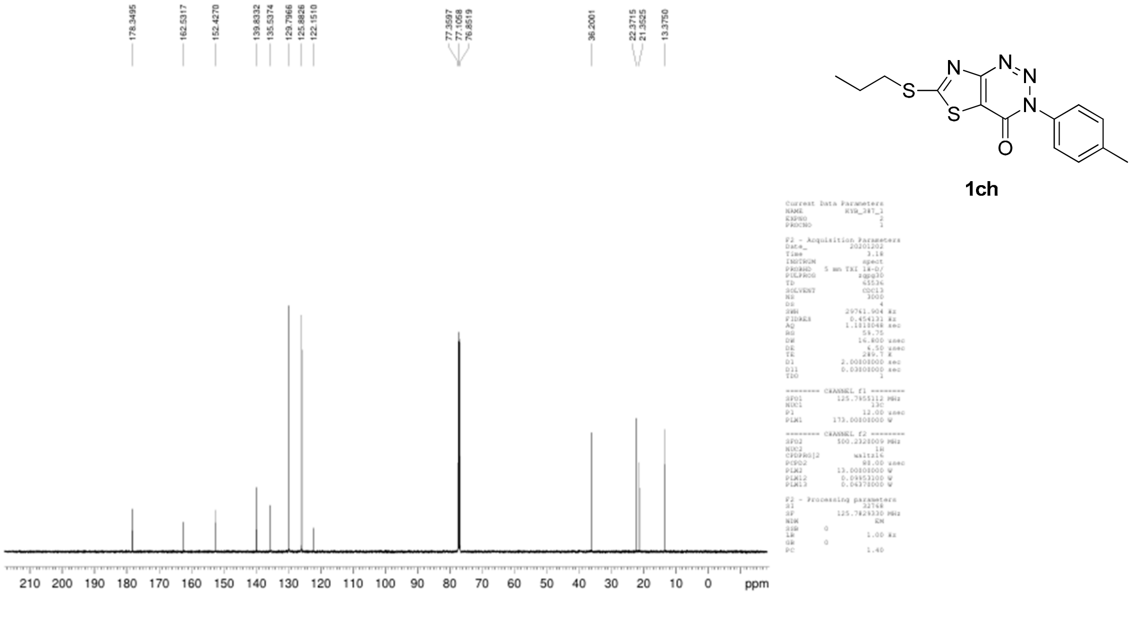
178.3495
162.5317
152.4270
139.8332
135.5374
129.7966
125.8826
122.1510
77.3597
77.1058
76.8519
36.2001
22.3715
21.3525
13.3750
1ch
210 200 190 180 170 160 150 140 130 120 110 100 90 80 70 60 50 40 30 20 10 0 ppm

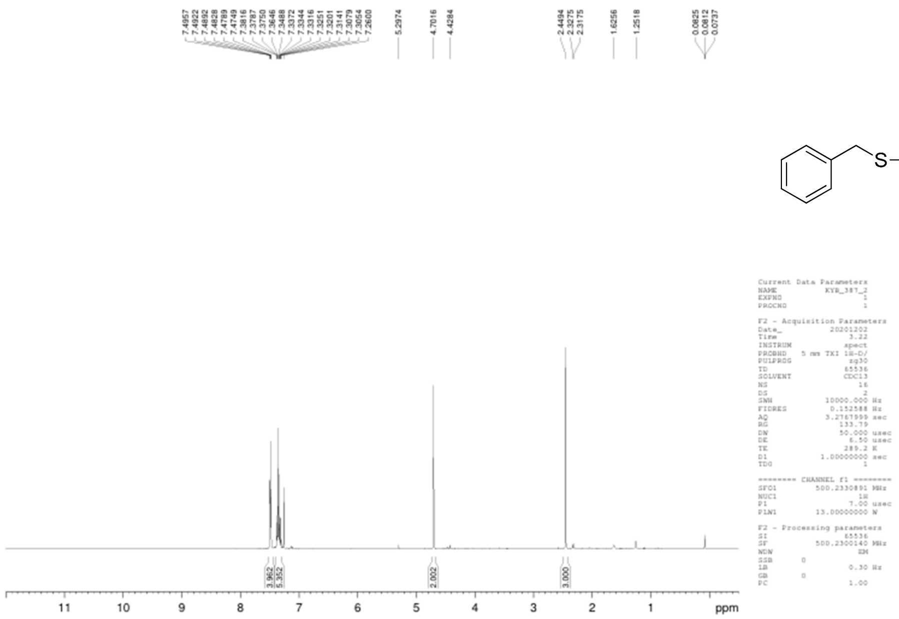
7.4957
7.4922
7.4892
7.4828
7.4789
7.4749
7.3816
7.3787
7.3750
7.3646
7.3488
7.3372
7.3344
7.3316
7.3251
7.3201
7.3141
7.3079
7.3054
7.2600
5.2974
4.7016
4.4284
2.4494
2.3275
2.3175
1.6256
1.2518
0.0825
0.0812
0.0737
3.962
5.352
2.002
3.000
ppm

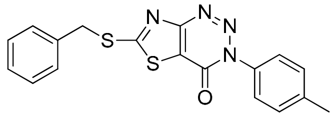
N
N
N
S
S
N
O

1ci

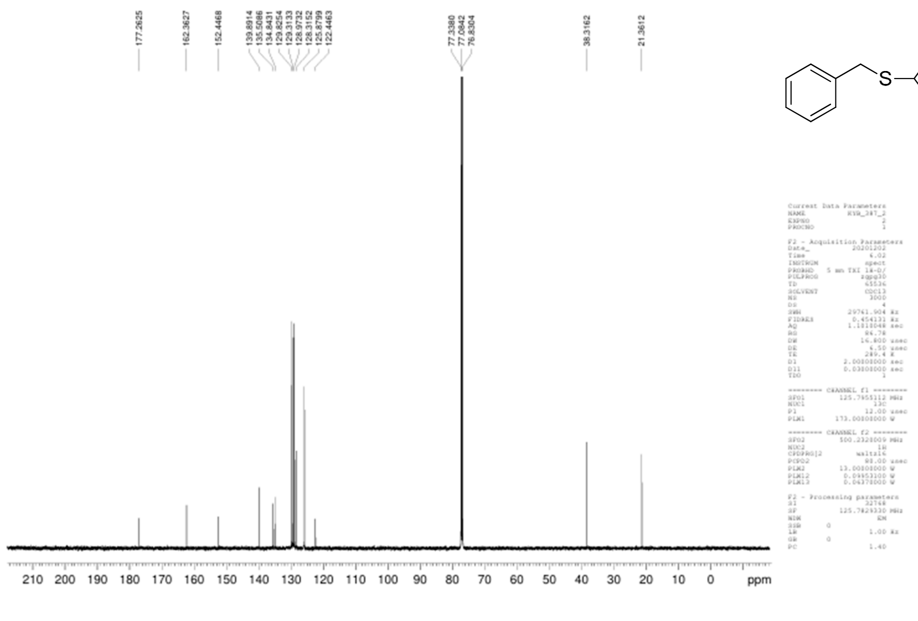
177.2625
162.3627
152.4468
139.8914
135.5086
134.8431
129.8254
129.3133
128.9732
128.3152
125.8799
122.4463
77.3380
77.0842
76.8304
38.3162
21.3612
ppm

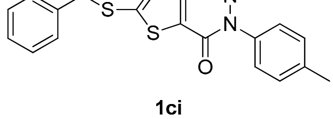
N
N
N
S
S
N
O

1ci

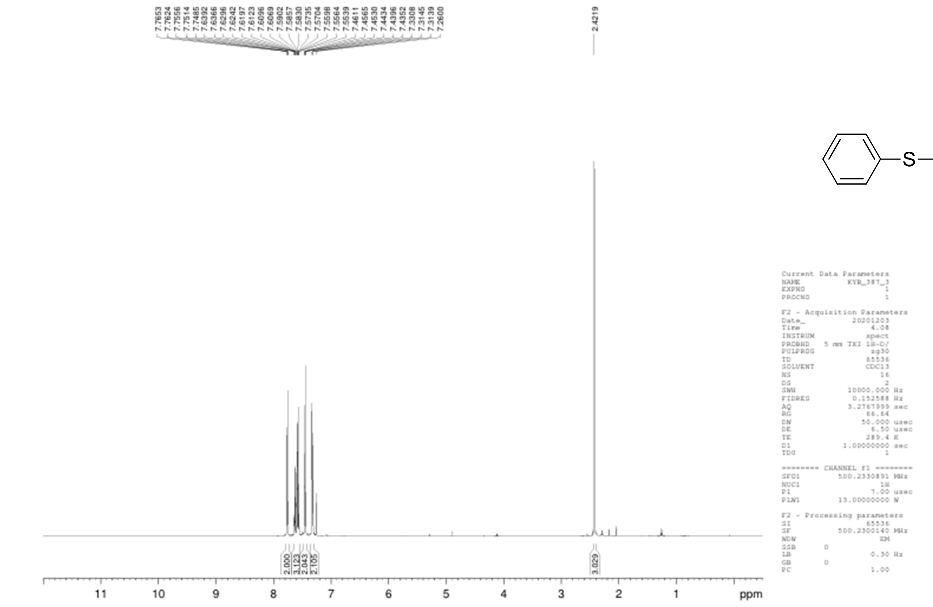
7.7653 7.7624 7.7556 7.7514 7.7485 7.6392 7.6366 7.6296 7.6242 7.6197 7.6123 7.6096 7.6069 7.5902 7.5857 7.5830 7.5735 7.5704 7.5598 7.5564 7.5539 7.4611 7.4565 7.4530 7.4434 7.4396 7.4352 7.3308 7.3145 7.3139 7.2600
2.4219
2.000 3.123 2.043 2.105
3.029
11 10 9 8 7 6 5 4 3 2 1 ppm

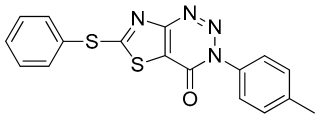
N
N
N
S
S
N
O

1cj

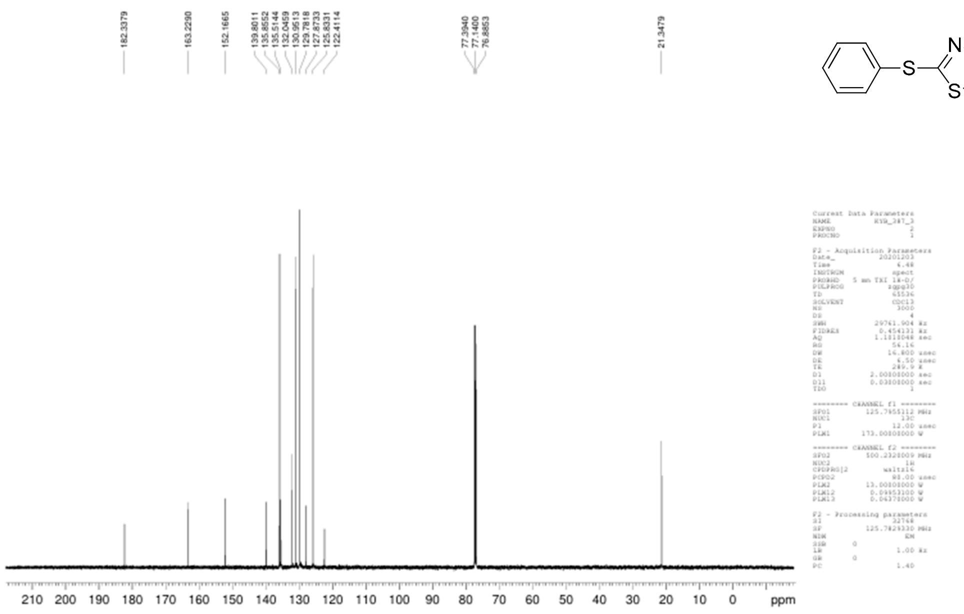
182.3379
163.2290
152.1665
139.8011 135.8552 135.5144 132.0459 130.9513 129.7818 127.8733 125.8331 122.4114
77.3940 77.1400 76.8853
21.3479
210 200 190 180 170 160 150 140 130 120 110 100 90 80 70 60 50 40 30 20 10 0 ppm

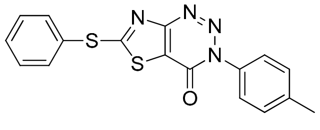
N
N
N
S
S
N
O

1cj

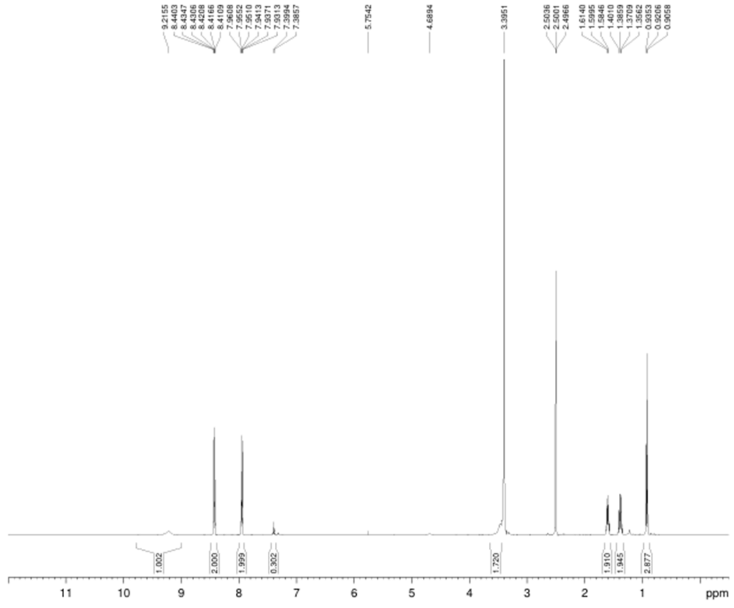

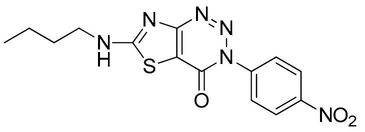

1da

```
Current Data Parameters
NAME       KYB_381_1_DMSO
EXPNO                   1
PROCNO                  1

F2 - Acquisition Parameters
Date_            20201207
Time                19.01
INSTRUM             spect
PROBHD   5 mm TXI 1H-D/
PULPROG              zg30
TD                  65536
SOLVENT              DMSO
NS                     16
DS                      2
SWH          10000.000 Hz
FIDRES        0.152588 Hz
AQ           3.2767999 sec
RG                  86.78
DW              50.000 usec
DE                6.50 usec
TE               290.5 K
D1          1.00000000 sec
TD0                     1

======== CHANNEL f1 ========
SFO1        500.2330891 MHz
NUC1                   1H
P1                7.00 usec
PLW1       13.00000000 W

F2 - Processing parameters
SI                  65536
SF          500.2300037 MHz
WDW                    EM
SSB      0
LB                0.30 Hz
GB       0
PC                   1.00
```

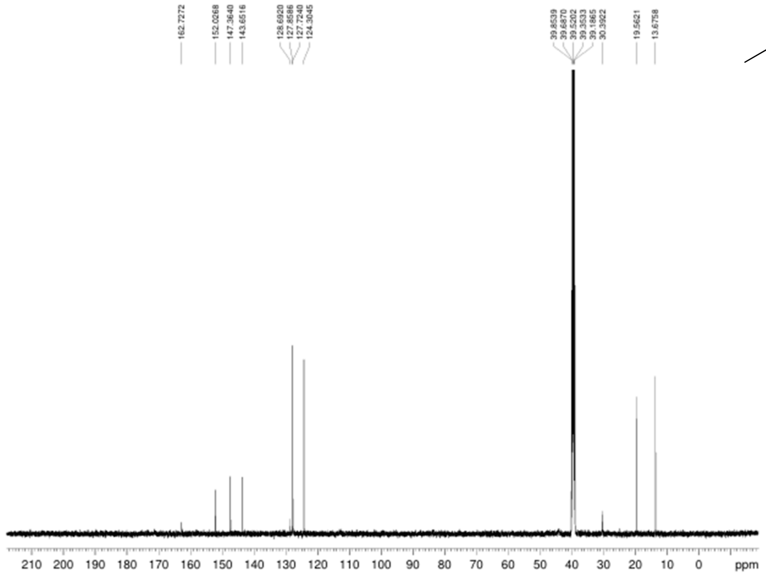

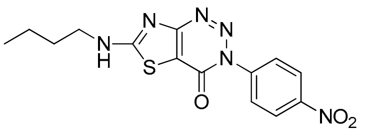

1da

```
Current Data Parameters
NAME       KYB_381_1_DMSO
EXPNO                   2
PROCNO                  1

F2 - Acquisition Parameters
Date_            20201207
Time                21.40
INSTRUM             spect
PROBHD   5 mm TXI 1H-D/
PULPROG            zgpg30
TD                  65536
SOLVENT              DMSO
NS                   3000
DS                      4
SWH          29761.904 Hz
FIDRES        0.454131 Hz
AQ           1.1010048 sec
RG                  92.36
DW              16.800 usec
DE                6.50 usec
TE               290.9 K
D1          2.00000000 sec
D11         0.03000000 sec
TD0                     1

======== CHANNEL f1 ========
SFO1        125.7955112 MHz
NUC1                  13C
P1               12.00 usec
PLW1      173.00000000 W

======== CHANNEL f2 ========
SFO2        500.2320009 MHz
NUC2                   1H
CPDPRG[2           waltz16
PCPD2            80.00 usec
PLW2       13.00000000 W
PLW12       0.09953100 W
PLW13       0.06378000 W

F2 - Processing parameters
SI                  32768
SF          125.7829823 MHz
WDW                    EM
SSB      0
LB                1.00 Hz
GB       0
PC                   1.40
```

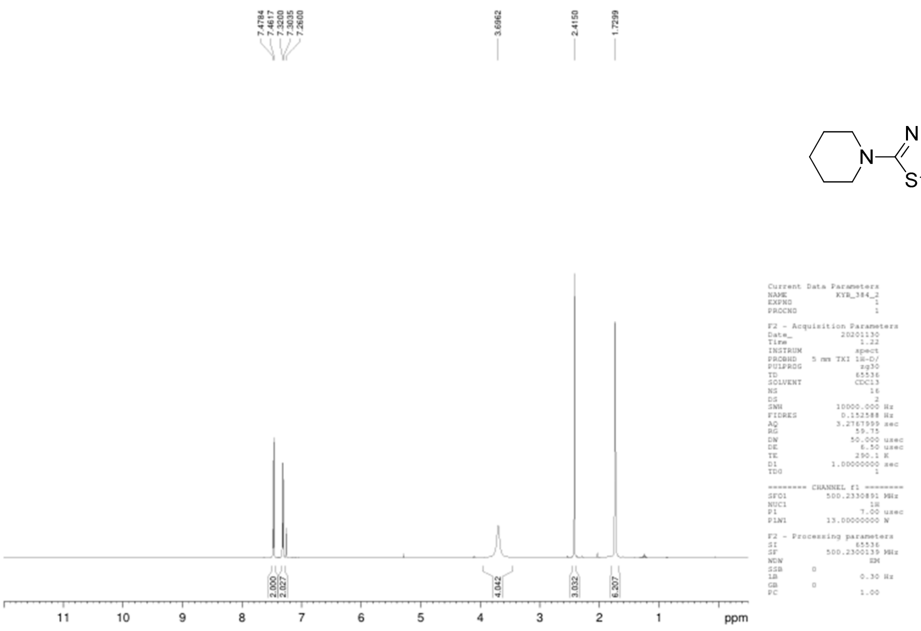

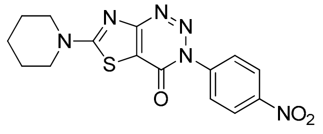

1db

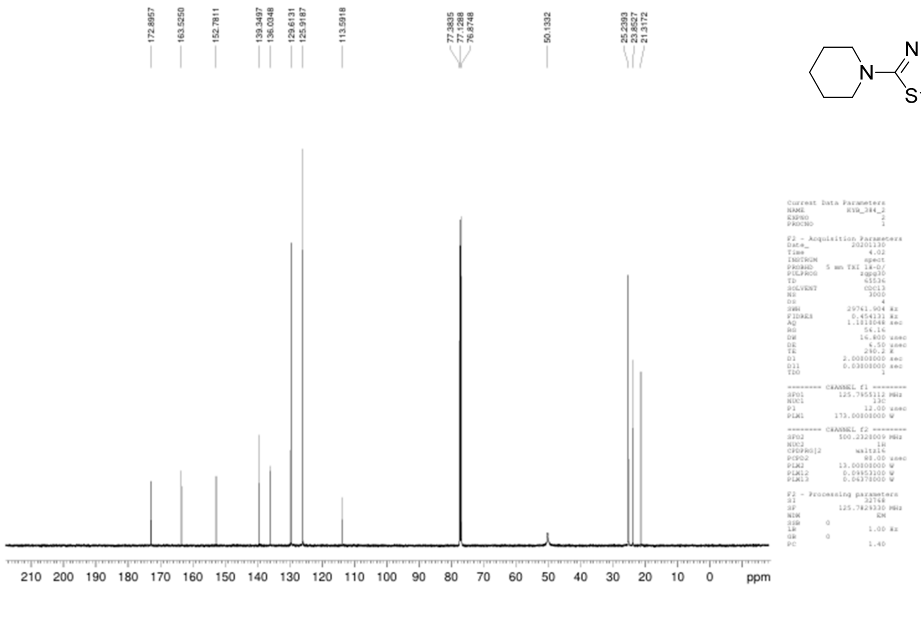

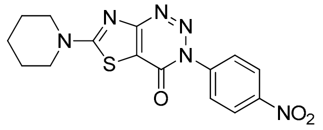

1db

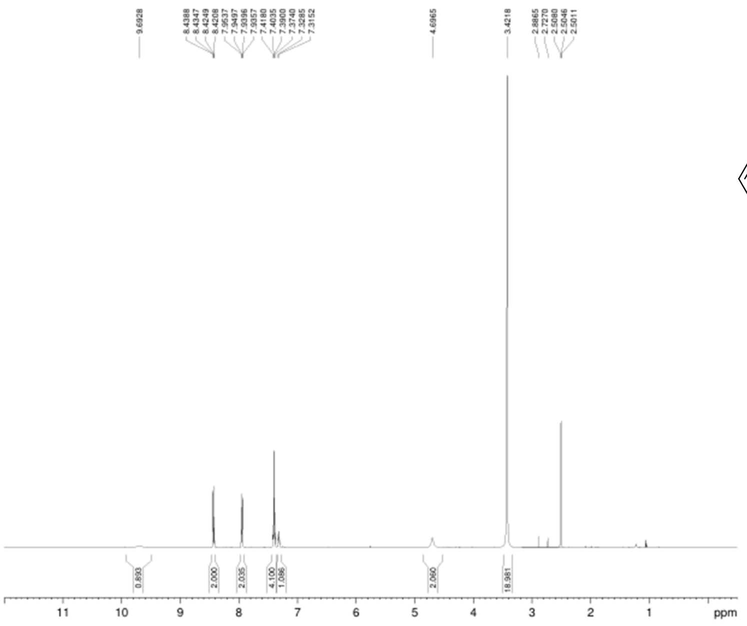

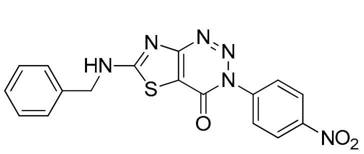

1dc

Current Data Parameters
NAME KYB_381_3_DMSO
EXPNO 1
PROCNO 1

F2 - Acquisition Parameters
Date_ 20201209
Time 3.54
INSTRUM spect
PROBHD 5 mm TXI 1H-D/
PULPROG zg30
TD 65536
SOLVENT DMSO
NS 16
DS 2
SWH 10000.000 Hz
FIDRES 0.152588 Hz
AQ 3.2767999 sec
RG 72.97
DW 50.000 usec
DE 6.50 usec
TE 287.6 K
D1 1.00000000 sec
TD0 1

======== CHANNEL f1 ========
SFO1 500.2330891 MHz
NUC1 1H
P1 7.00 usec
PLW1 13.00000000 W

F2 - Processing parameters
SI 65536
SF 500.2300000 MHz
WDW EM
SSB 0
LB 0.30 Hz
GB 0
PC 1.00

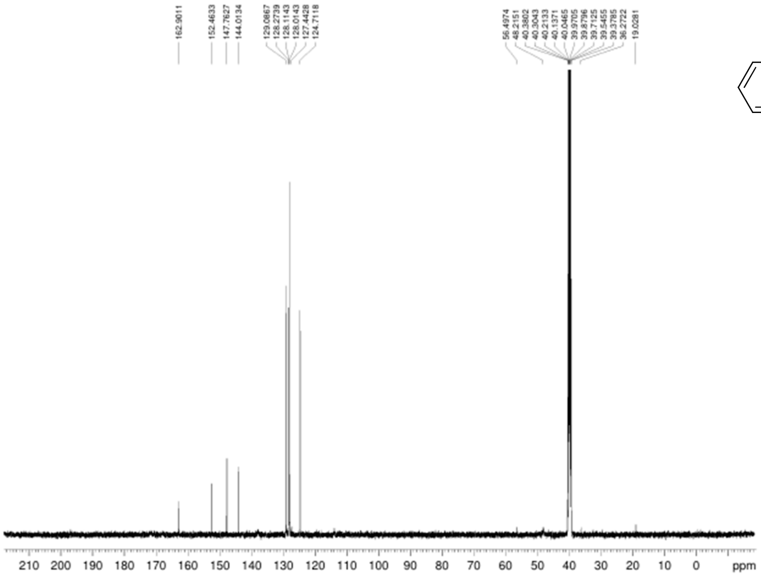

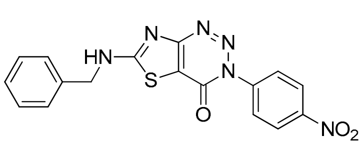

1dc

Current Data Parameters
NAME KYB_381_3_DMSO
EXPNO 2
PROCNO 1

F2 - Acquisition Parameters
Date_ 20201209
Time 6.45
INSTRUM spect
PROBHD 5 mm TXI 1H-D/
PULPROG zgpg30
TD 65536
SOLVENT DMSO
NS 3200
DS 4
SWH 29761.904 Hz
FIDRES 0.454131 Hz
AQ 1.1010048 sec
RG 59.75
DW 16.800 usec
DE 6.50 usec
TE 287.7 K
D1 2.00000000 sec
D11 0.03000000 sec
TD0 1

======== CHANNEL f1 ========
SFO1 125.7955112 MHz
NUC1 13C
P1 12.00 usec
PLW1 173.00000000 W

======== CHANNEL f2 ========
SFO2 500.2320009 MHz
NUC2 1H
CPDPRG[2 waltz16
PCPD2 80.00 usec
PLW2 13.00000000 W
PLW12 0.09953100 W
PLW13 0.06378000 W

F2 - Processing parameters
SI 32768
SF 125.7829330 MHz
WDW EM
SSB 0
LB 1.00 Hz
GB 0
PC 1.40

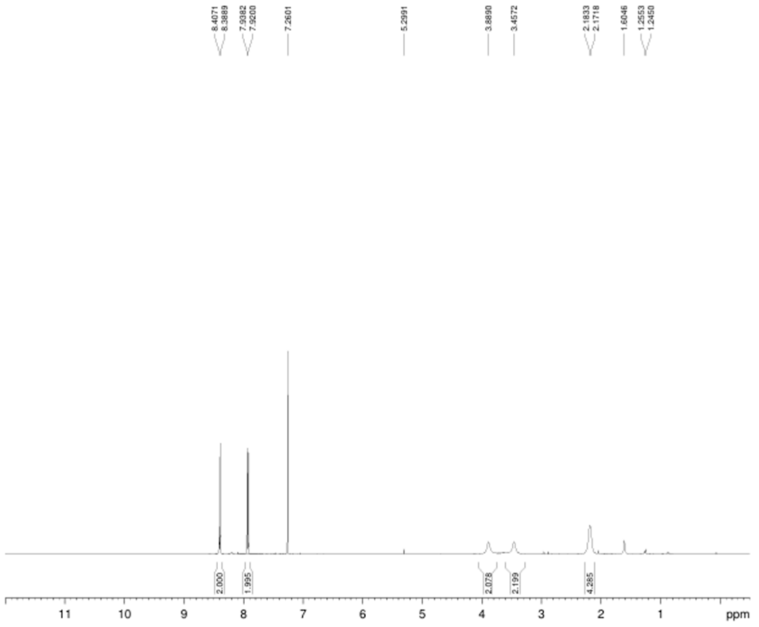

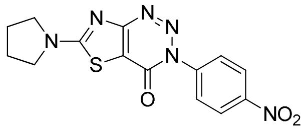

1dd

```
Current Data Parameters
NAME          KYR_381_4
EXPNO                 1
PROCNO                1

F2 - Acquisition Parameters
Date_          20201124
Time               7.19
INSTRUM           spect
PROBHD   5 mm TXI 1H-D/
PULPROG            zg30
TD                65536
SOLVENT           CDCl3
NS                   16
DS                    2
SWH           10000.000 Hz
FIDRES         0.152588 Hz
AQ            3.2767999 sec
RG               273.43
DW               50.000 usec
DE                 6.50 usec
TE                290.8 K
D1           1.00000000 sec
TD0                   1

======== CHANNEL f1 ========
SFO1        500.2330891 MHz
NUC1                 1H
P1                 7.00 usec
PLW1        13.00000000 W

F2 - Processing parameters
SI                65536
SF          500.2300146 MHz
WDW                  EM
SSB      0
LB                 0.30 Hz
GB       0
PC                 1.00
```

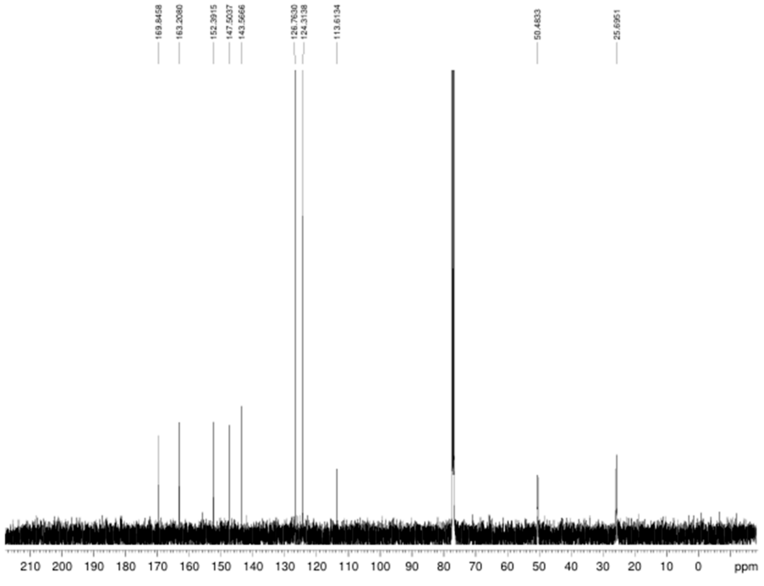

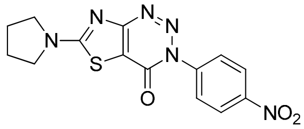

1dd

```
Current Data Parameters
NAME          KYR_381_4
EXPNO                 2
PROCNO                1

F2 - Acquisition Parameters
Date_          20201124
Time               9.59
INSTRUM           spect
PROBHD   5 mm TXI 1H-D/
PULPROG          zgpg30
TD                65536
SOLVENT           CDCl3
NS                 3000
DS                    4
SWH           29761.904 Hz
FIDRES         0.454131 Hz
AQ            1.1010048 sec
RG                92.36
DW               16.800 usec
DE                 6.50 usec
TE                291.7 K
D1           2.00000000 sec
D11          0.03000000 sec
TD0                   1

======== CHANNEL f1 ========
SFO1        125.7955112 MHz
NUC1                13C
P1                12.00 usec
PLW1       173.00000000 W

======== CHANNEL f2 ========
SFO2        500.2320009 MHz
NUC2                 1H
CPDPRG[2        waltz16
PCPD2             80.00 usec
PLW2        13.00000000 W
PLW12        0.09953100 W
PLW13        0.06378000 W

F2 - Processing parameters
SI                32768
SF          125.7829330 MHz
WDW                  EM
SSB      0
LB                 1.00 Hz
GB       0
PC                 1.40
```

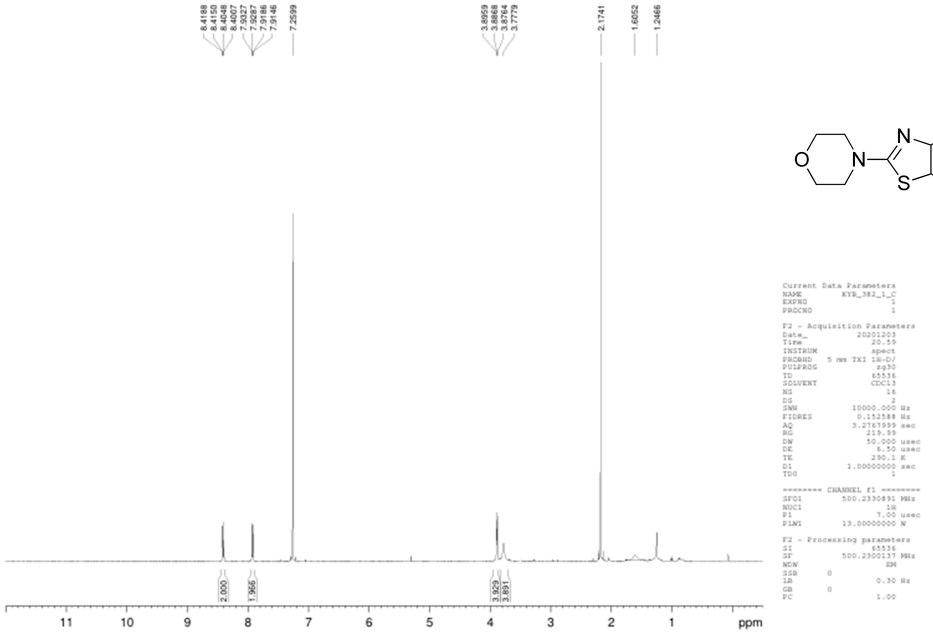

8.4188
8.4150
8.4048
8.4007
7.9327
7.9287
7.9186
7.9146
7.2599
3.8959
3.8868
3.8764
3.7779
2.1741
1.6052
1.2466
2.000
1.966
3.929
3.891
11 10 9 8 7 6 5 4 3 2 1 ppm
Current Data Parameters
NAME KYB_382_1_C
EXPNO 1
PROCNO 1
F2 - Acquisition Parameters
Date_ 20201203
Time 20.59
INSTRUM spect
PROBHD 5 mm TXI 1H-D/
PULPROG zg30
TD 65536
SOLVENT CDCl3
NS 16
DS 2
SWH 10000.000 Hz
FIDRES 0.152588 Hz
AQ 3.2767999 sec
RG 219.99
DW 50.000 usec
DE 6.50 usec
TE 290.1 K
D1 1.00000000 sec
TD0 1
======== CHANNEL f1 ========
SFO1 500.2330891 MHz
NUC1 1H
P1 7.00 usec
PLW1 13.00000000 W
F2 - Processing parameters
SI 65536
SF 500.2300137 MHz
WDW EM
SSB 0
LB 0.30 Hz
GB 0
PC 1.00

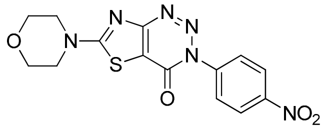

1de

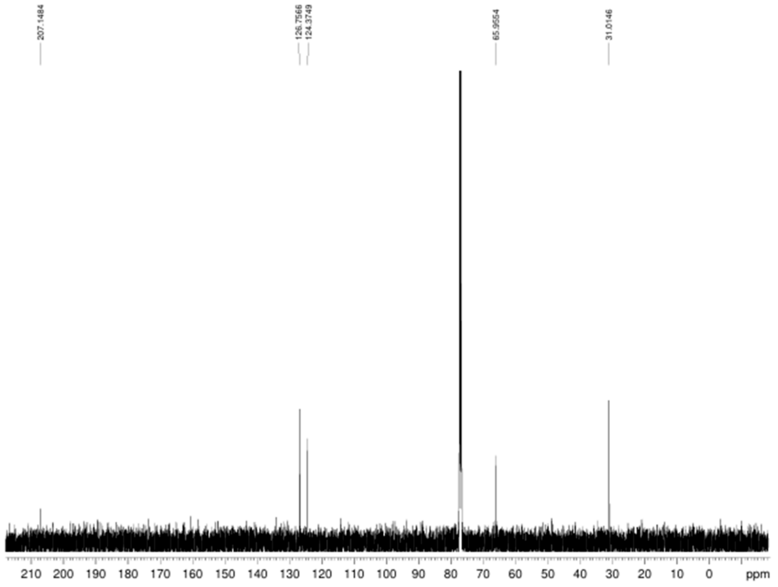

207.1484
126.7566
124.3749
65.9554
31.0146
210 200 190 180 170 160 150 140 130 120 110 100 90 80 70 60 50 40 30 20 10 0 ppm

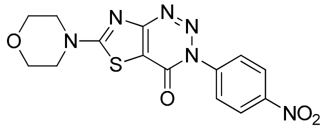

1de

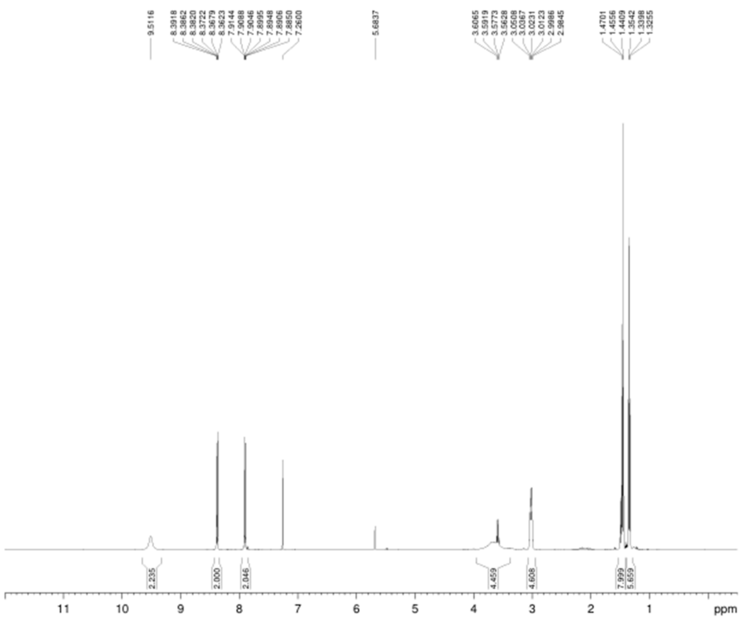

9.5116
8.3918
8.3862
8.3820
8.3722
8.3679
8.3623
7.9144
7.9088
7.9046
7.8995
7.8948
7.8906
7.8850
7.2600
5.6837
3.6065
3.5919
3.5773
3.5628
3.0508
3.0367
3.0231
3.0123
2.9986
2.9845
1.4701
1.4556
1.4409
1.3542
1.3398
1.3255
2.235
2.000
2.046
4.459
4.608
7.999
5.659
11
10
9
8
7
6
5
4
3
2
1
ppm
Current Data Parameters
NAME KYB_382_2
EXPNO 1
PROCNO 1
F2 - Acquisition Parameters
Date_ 20201127
Time 3.17
INSTRUM spect
PROBHD 5 mm TXI 1H-D/
PULPROG zg30
TD 65536
SOLVENT CDCl3
NS 16
DS 2
SWH 10000.000 Hz
FIDRES 0.152588 Hz
AQ 3.2767999 sec
RG 104.49
DW 50.000 usec
DE 6.50 usec
TE 293.3 K
D1 1.00000000 sec
TD0 1
======== CHANNEL f1 ========
SFO1 500.2330891 MHz
NUC1 1H
P1 7.00 usec
PLW1 13.00000000 W
F2 - Processing parameters
SI 65536
SF 500.2300147 MHz
WDW EM
SSB 0
LB 0.30 Hz
GB 0
PC 1.00

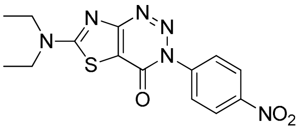

N
N
N
N
S
O
NO2

1df

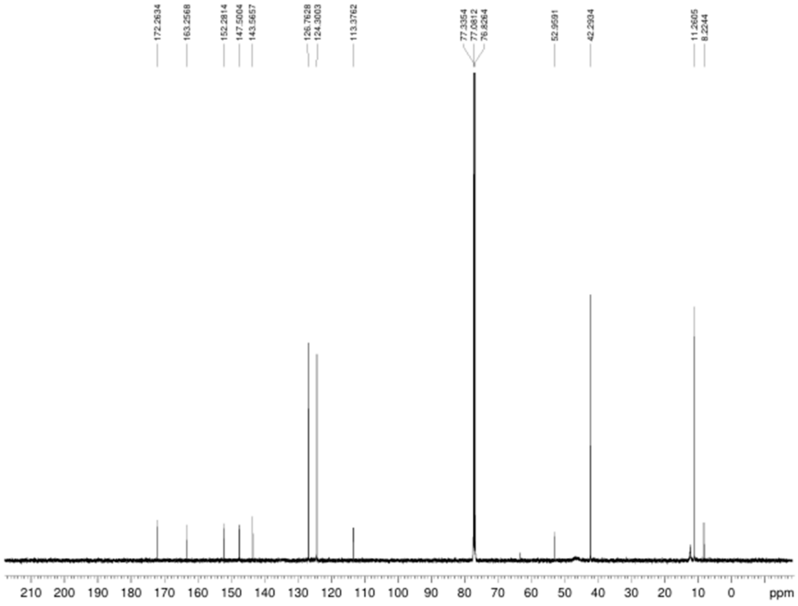

172.2634
163.2568
152.2814
147.5004
143.5657
126.7628
124.3003
113.3762
77.3354
77.0812
76.8264
52.9591
42.2934
11.2605
8.2244
210
200
190
180
170
160
150
140
130
120
110
100
90
80
70
60
50
40
30
20
10
0
ppm
Current Data Parameters
NAME KYB_382_2
EXPNO 2
PROCNO 1
F2 - Acquisition Parameters
Date_ 20201127
Time 6.23
INSTRUM spect
PROBHD 5 mm TXI 1H-D/
PULPROG zgpg30
TD 65536
SOLVENT CDCl3
NS 3500
DS 4
SWH 29761.904 Hz
FIDRES 0.454131 Hz
AQ 1.1010048 sec
RG 59.75
DW 16.800 usec
DE 6.50 usec
TE 294.0 K
D1 2.00000000 sec
D11 0.03000000 sec
TD0 1
======== CHANNEL f1 ========
SFO1 125.7955112 MHz
NUC1 13C
P1 12.00 usec
PLW1 173.00000000 W
======== CHANNEL f2 ========
SFO2 500.2320009 MHz
NUC2 1H
CPDPRG[2 waltz16
PCPD2 80.00 usec
PLW2 13.00000000 W
PLW12 0.09953100 W
PLW13 0.06378000 W
F2 - Processing parameters
SI 32768
SF 125.7829330 MHz
WDW EM
SSB 0
LB 1.00 Hz
GB 0
PC 1.40

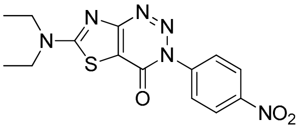

N
N
N
N
S
O
NO2

1df

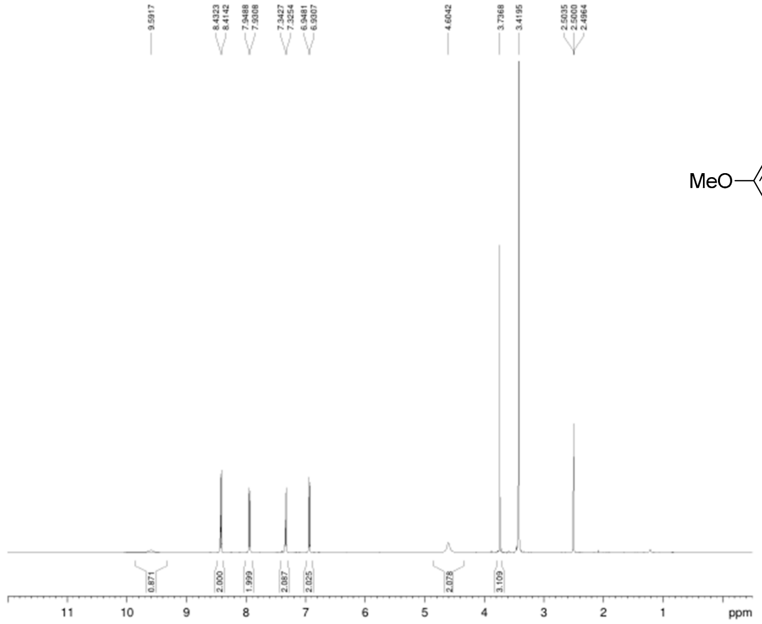

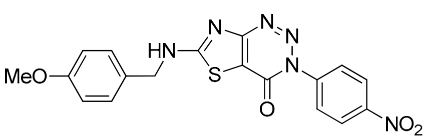

1dg

```
Current Data Parameters
NAME      KYB_382_3_DMSO
EXPNO                  1
PROCNO                 1

F2 - Acquisition Parameters
Date_           20201209
Time                6.49
INSTRUM            spect
PROBHD    5 mm TXI 1H-D/
PULPROG             zg30
TD                 65536
SOLVENT             DMSO
NS                    16
DS                     2
SWH            10000.000 Hz
FIDRES          0.152588 Hz
AQ             3.2767999 sec
RG                 66.64
DW                50.000 usec
DE                  6.50 usec
TE                 287.1 K
D1            1.00000000 sec
TD0                    1

======== CHANNEL f1 ========
SFO1         500.2330891 MHz
NUC1                  1H
P1                  7.00 usec
PLW1         13.00000000 W

F2 - Processing parameters
SI                 65536
SF           500.2300014 MHz
WDW                   EM
SSB      0
LB                  0.30 Hz
GB       0
PC                  1.00
```

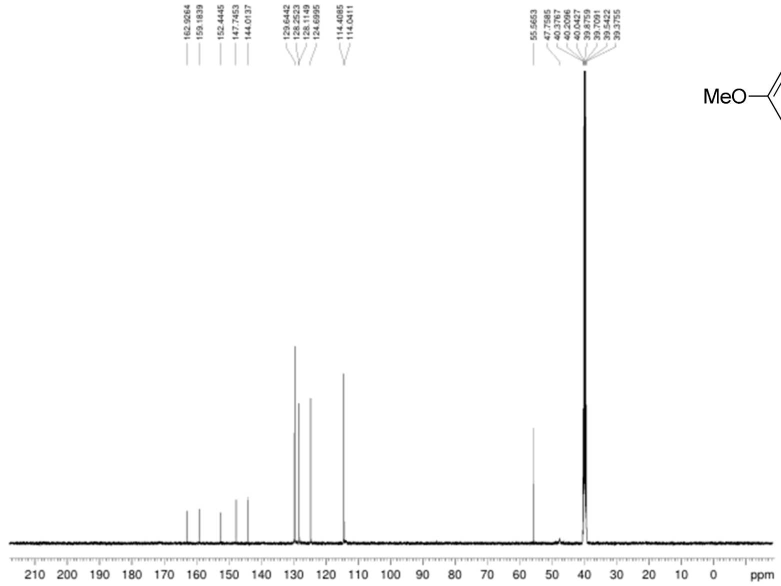

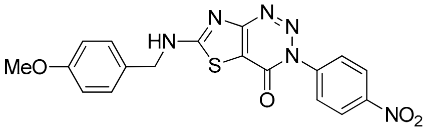

1dg

```
Current Data Parameters
NAME      KYB_382_3_DMSO
EXPNO                  2
PROCNO                 1

F2 - Acquisition Parameters
Date_           20201209
Time                9.39
INSTRUM            spect
PROBHD    5 mm TXI 1H-D/
PULPROG          zgpg30
TD                 65536
SOLVENT             DMSO
NS                  3200
DS                     4
SWH            29761.904 Hz
FIDRES          0.454131 Hz
AQ             1.1010048 sec
RG                 92.36
DW                16.800 usec
DE                  6.50 usec
TE                 288.0 K
D1            2.00000000 sec
D11           0.03000000 sec
TD0                    1

======== CHANNEL f1 ========
SFO1         125.7955112 MHz
NUC1                 13C
P1                 12.00 usec
PLW1        173.00000000 W

======== CHANNEL f2 ========
SFO2         500.2320009 MHz
NUC2                  1H
CPDPRG[2          waltz16
PCPD2              80.00 usec
PLW2         13.00000000 W
PLW12         0.09953100 W
PLW13         0.06378000 W

F2 - Processing parameters
SI                 32768
SF           125.7829330 MHz
WDW                   EM
SSB      0
LB                  1.00 Hz
GB       0
PC                  1.40
```

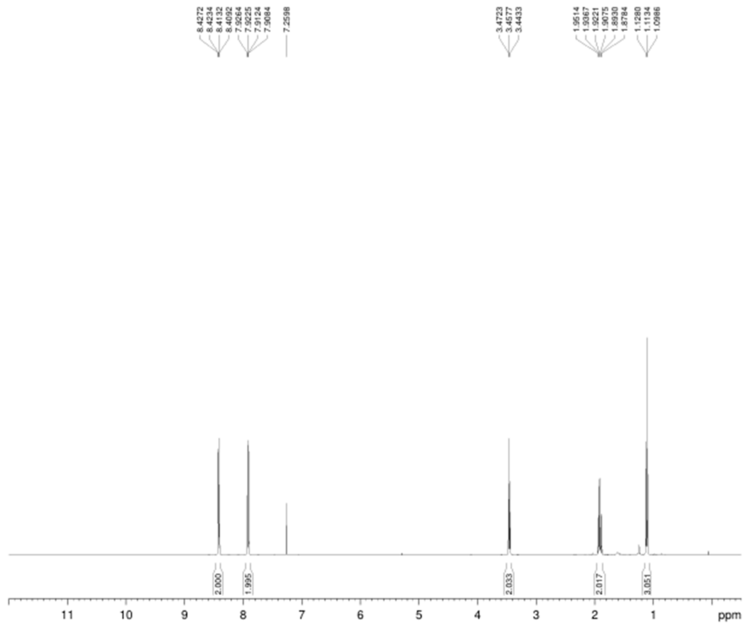
8.4272
8.4234
8.4132
8.4092
7.9264
7.9225
7.9124
7.9084
7.2598
3.4723
3.4577
3.4433
1.9514
1.9367
1.9221
1.9075
1.8930
1.8784
1.1280
1.1134
1.0986
2.000
1.995
2.033
2.017
3.051
11
10
9
8
7
6
5
4
3
2
1
ppm

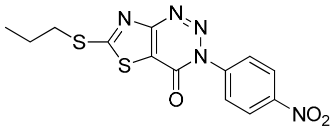
N
N
N
S
N
S
O
NO2

1dh

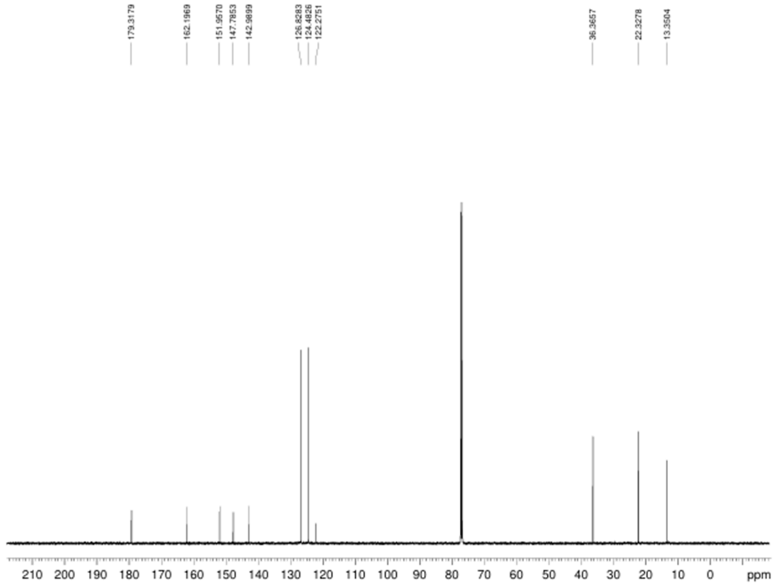
179.3179
162.1969
151.9570
147.7853
142.9899
126.8283
124.4826
122.2751
36.3657
22.3278
13.3504
210
200
190
180
170
160
150
140
130
120
110
100
90
80
70
60
50
40
30
20
10
0
ppm

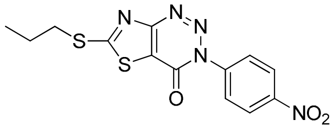
N
N
N
S
N
S
O
NO2

1dh

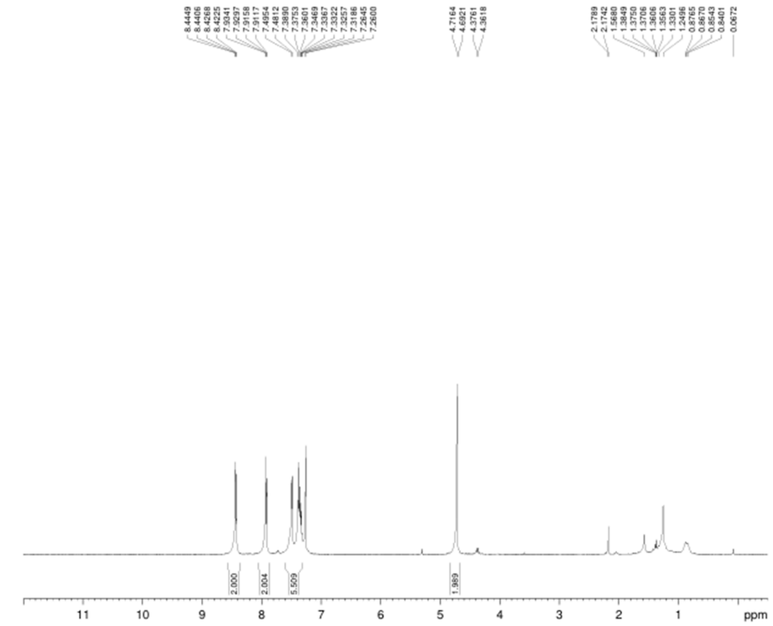
8.4449 8.4406 8.4268 8.4225 7.9341 7.9297 7.9158 7.9117 7.4954 7.4812 7.3890 7.3753 7.3601 7.3469 7.3367 7.3322 7.3257 7.3186 7.2645 7.2600
4.7164 4.6921 4.3761 4.3618
2.1789 2.1742 1.5680 1.3849 1.3750 1.3706 1.3606 1.3563 1.3301 1.2496 0.8765 0.8670 0.8543 0.8401 0.0672
2.000
2.004
5.509
1.989
11 10 9 8 7 6 5 4 3 2 1 ppm

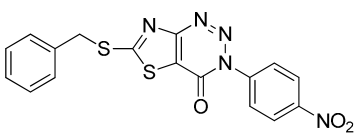
N
N
N
S
S
N
O
NO_2

1di

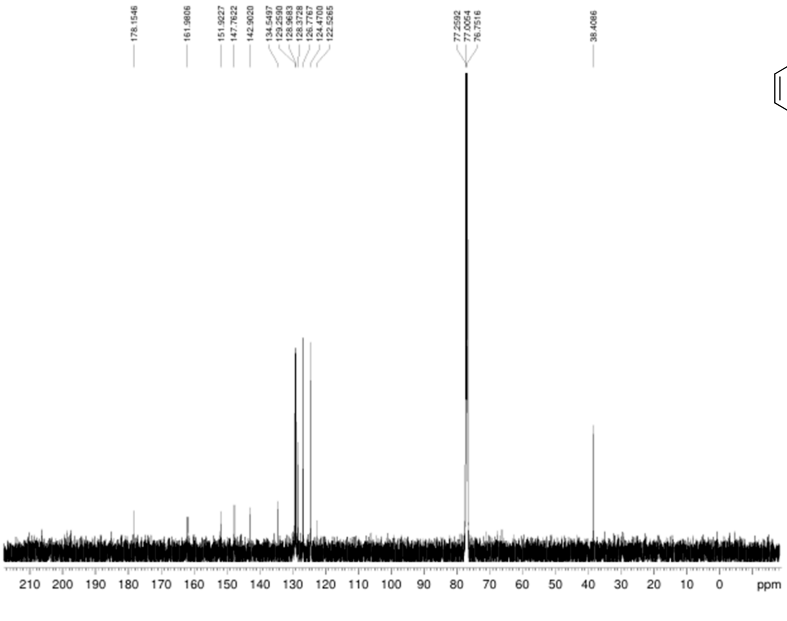
178.1546
161.9806
151.9227
147.7622
142.9020
134.5497
129.2590
128.9683
128.3728
126.7767
124.4700
122.5265
77.2592
77.0054
76.7516
38.4086
210 200 190 180 170 160 150 140 130 120 110 100 90 80 70 60 50 40 30 20 10 0 ppm

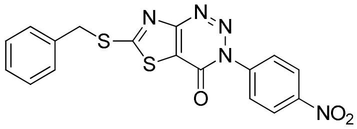
N
N
N
S
S
N
O
NO_2

1di

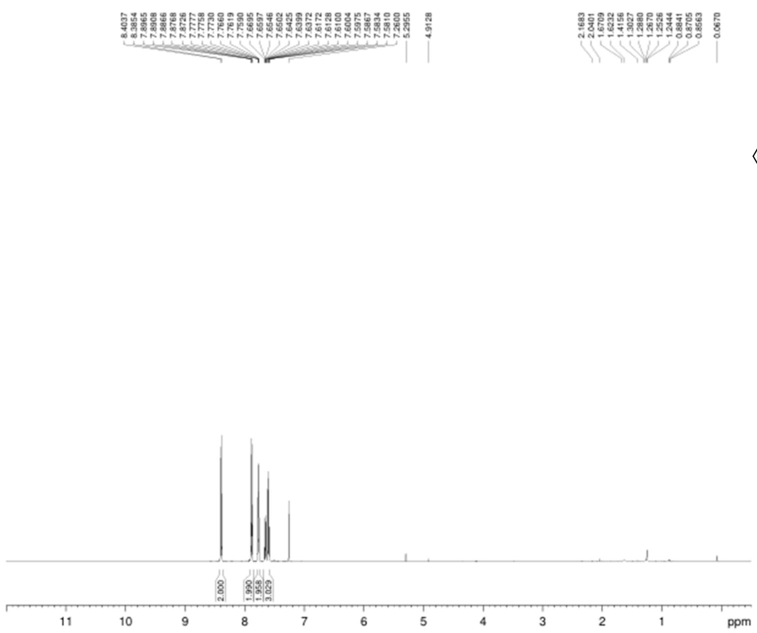

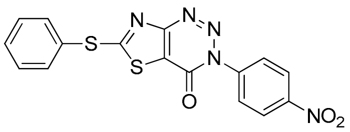

1dj

```
Current Data Parameters
NAME          KYR_383_3
EXPNO                 1
PROCNO                1

F2 - Acquisition Parameters
Date_          20201124
Time              21.51
INSTRUM           spect
PROBHD   5 mm TXI 1H-D/
PULPROG            zg30
TD                65536
SOLVENT           CDCl3
NS                   16
DS                    2
SWH         10000.000 Hz
FIDRES       0.152588 Hz
AQ          3.2767999 sec
RG             192.71
DW             50.000 usec
DE               6.50 usec
TE          0 K
D1         1.00000000 sec
TD0                   1

======== CHANNEL f1 ========
SFO1      500.2330891 MHz
NUC1                 1H
P1               7.00 usec
PLW1      13.00000000 W

F2 - Processing parameters
SI                65536
SF        500.2300146 MHz
WDW                  EM
SSB         0
LB               0.30 Hz
GB          0
PC               1.00
```

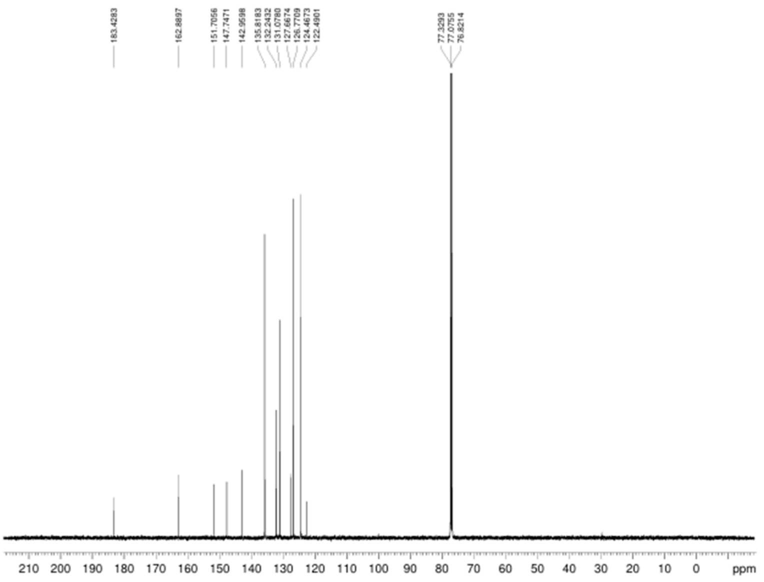

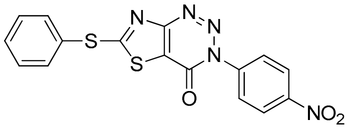

1dj

```
Current Data Parameters
NAME          KYR_383_3
EXPNO                 2
PROCNO                1

F2 - Acquisition Parameters
Date_          20201125
Time               8.30
INSTRUM           spect
PROBHD   5 mm TXI 1H-D/
PULPROG          zgpg30
TD                65536
SOLVENT           CDCl3
NS                 3000
DS                    4
SWH         29761.904 Hz
FIDRES       0.454131 Hz
AQ          1.1010048 sec
RG              59.75
DW             16.800 usec
DE               6.50 usec
TE          0 K
D1         2.00000000 sec
D11        0.03000000 sec
TD0                   1

======== CHANNEL f1 ========
SFO1      125.7955112 MHz
NUC1                13C
P1              12.00 usec
PLW1     173.00000000 W

======== CHANNEL f2 ========
SFO2      500.2320009 MHz
NUC2                 1H
CPDPRG[2        waltz16
PCPD2           80.00 usec
PLW2      13.00000000 W
PLW12      0.09953100 W
PLW13      0.06378000 W

F2 - Processing parameters
SI                32768
SF        125.7829330 MHz
WDW                  EM
SSB         0
LB               1.00 Hz
GB          0
PC               1.40
```
